# Supplementary material for: Conformational Preferences and Antiproliferative Activity of Peptidomimetics Containing Methyl 1′-Aminoferrocene-1-carboxylate and Turn-Forming Homo- and Heterochiral Pro-Ala Motifs
Source: Int J Mol Sci. 2021 Dec 16;22(24):13532. doi: 10.3390/ijms222413532 (PMC8705031; doi:10.3390/ijms222413532)
Supplement: Supplementary file 1 [file ijms-22-13532-s001.zip › ijms-1501578-supplementary.pdf]

Conformational preferences and antiproliferative activity of peptidomimetics containing methyl 1'-aminoferrocene-1-carboxylate and turn forming homo- and heterochiral Pro-Ala motifs

Monika Kovačević, Mojca Čakić Semenčić, Kristina Radošević, Krešimir Molčanov, Sunčica Roca, Lucija Šimunović, Ivan Kodrin and Lidija Barišić

Supplementary Material

Content:

DFT data

MS, NMR and IR data

Boc-D-Pro-L-Ala-NH-Fn-COOMe **(2)**

Ac-D-Pro-L-Ala-NH-Fn-COOMe **(3)**

Boc-L-Pro-L-Ala-NH-Fn-COOMe **(4)**

Ac-L-Pro-L-Ala-NH-Fn-COOMe **(5)**

The influence of increased temperature on the *cis*/*trans* signals coalescence in peptides **2-5**

The influence of DMSO on *cis-trans* isomerization of a proline imide bond in peptides **2-5**

X-ray crystal structure analysis

Biological evaluation

DFT data

Table S1. Relative energies (reported energies refer to standard Gibbs free energies at 298 K in kJ mol^{-1}) of the most stable conformers of compounds **2–5**. Optimizations performed at the B3LYP-D3/6-311+G(d,p), LanL2DZ for Fe, level of theory, SMD model for solvent effects. Stereochemical descriptors and helicity determined from the value of pseudotorsion angles, intramolecular hydrogen bond patterns (IHB) labelled as in Figure 5, X–Y distances (in Å) of the selected X–H···Y hydrogen bonds connecting the *n*-membered rings.

type	stereochemical descriptors	Δ*E* / kJ mol^{-1}	ω / ° pseudotorsion angle	IHBs pattern	$NH_{Fc}\cdots O{=}C_{Boc/Ac}$ 10-membered	$NH_{Ala}\cdots O{=}C_{COOMe}$ 9-membered
**2-1**	*P*-1,2'	0.00	+43.2	**A**	2.95	2.98
**2-2**	*P*-1,2'	1.41	+43.2	**A**	2.97	2.98
**2-3**	*M*-1,1'	1.79	–35.1	**A**	2.91	2.89
**3-1**	*P*-1,2'	0.00	+41.2	**A**	2.92	2.96
**3-2**	*M*-1,1'	0.06	–32.7	**A**	2.90	2.93
**3-3**	*M*-1,1'	0.87	–31.8	**A**	2.96	2.90
**3-4**	*P*-1,2'	3.49	+40.8	**A**	2.96	2.97
**4-1**	*M*-1,1'	0.00	–28.4	**B**		2.93
**4-2**	*M*-1,2'	0.71	–74.1	**C**	2.92	
**4-3**	*M*-1,1'	2.38	–24.2	**C**	2.98	
**4-4**	*M*-1,2'	2.65	–79.0	**C**	2.88	
**4-5**	*M*-1,1'	3.98	–27.7	**B**		2.92
**5-1**	*M*-1,1'	0.00	–20.5	**A**	3.16	3.21
**5-2**	*M*-1,1'	4.60	–24.3	**B**		2.93
**5-3**	*M*-1,1'	5.98	–27.9	**B**		2.93

Boc-D-Pro-L-Ala-NH-Fn-COOMe (2)

Ion type	Calc. mass	Measured mass	Mass error / ppm	Mol. Formula	Int. CAL
M+	527.1719	527.1708	2.1	$C_{25}H_{33}N_3O_6Fe$	azitromicin

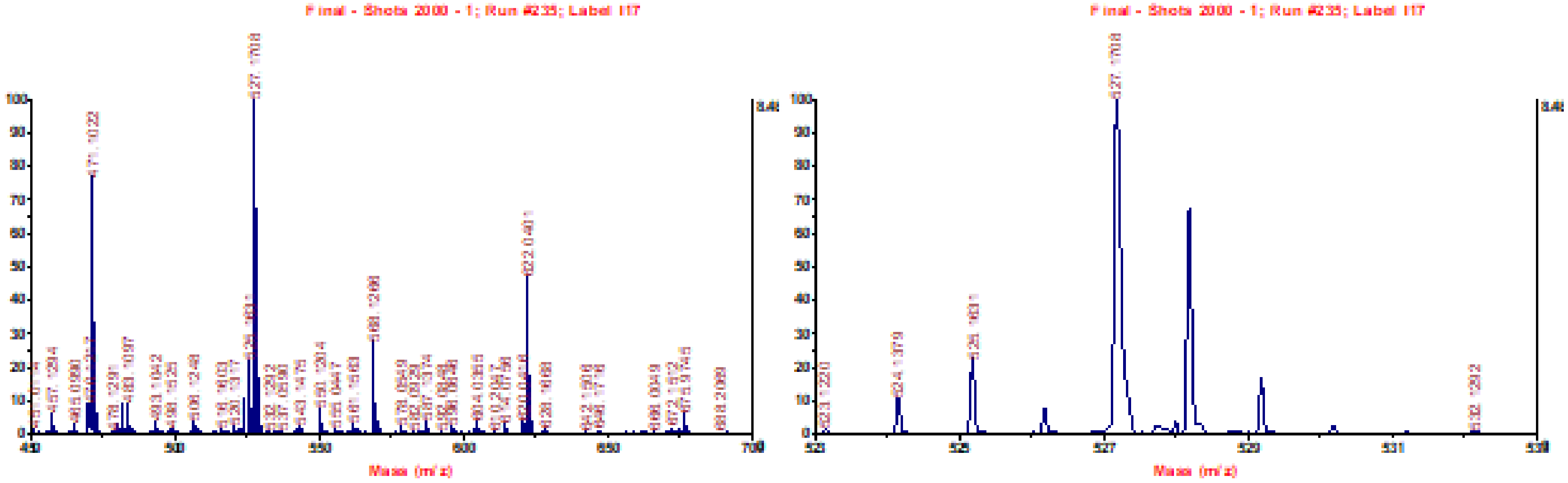

Figure S2. HRMS spectrum of compound **2.**

Qualitative Analysis Report

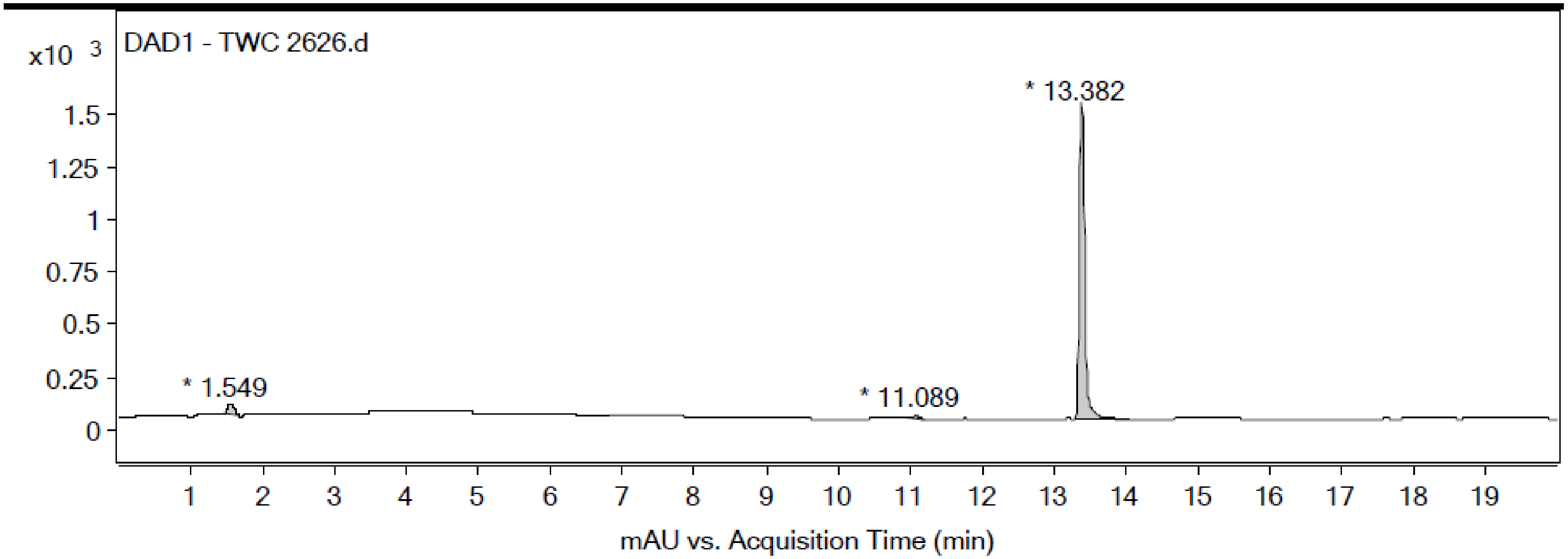

Integration Peak List

Peak	Start	RT	End	Height	Area	Area %
1	1.462	1.549	1.656	45.16	305.73	3.78
2	10.916	11.089	11.189	16.1	81.62	1.01
3	13.296	13.382	14.062	1513.94	8085.83	100

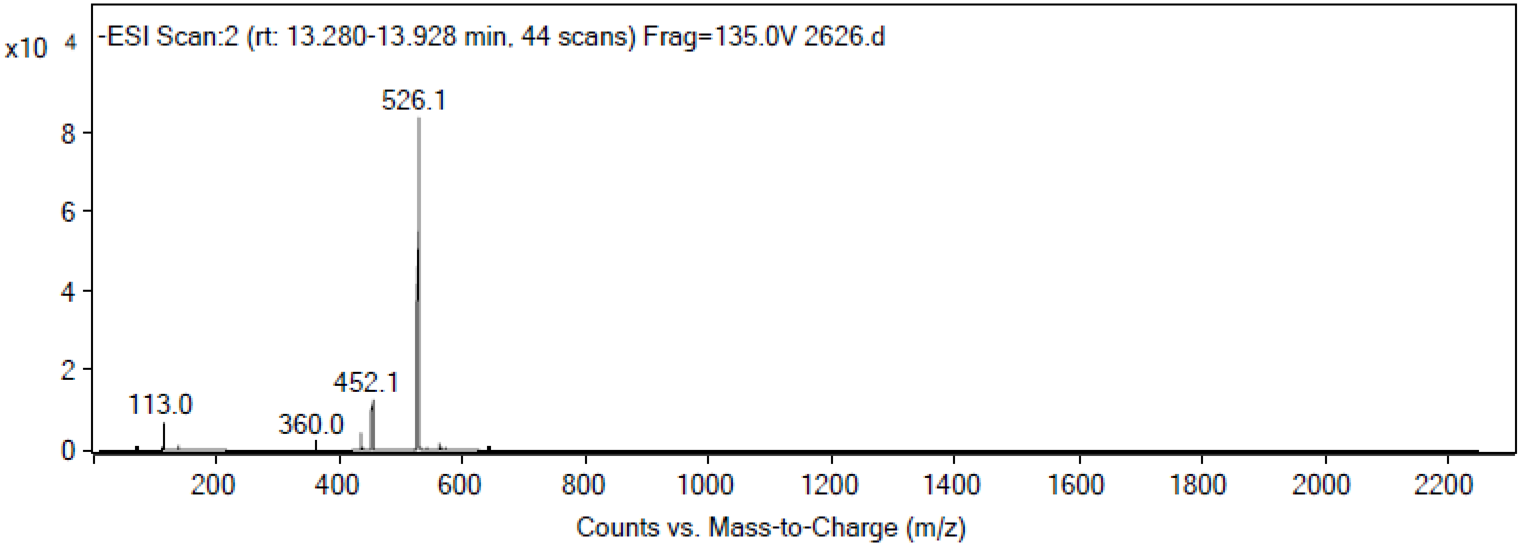

Peak List

m/z	z	Abund
113		6729.59
360		1854.58
434.1		3931.85
452.1	1	12571.55
453.1	1	3524.29
524.1		4976.46
526.1	1	83682.7
527.2	1	25827.74
528.1	1	5049.3
562.1		1647.65

Figure S3. HPLC-ESI spectra of compound **2.**

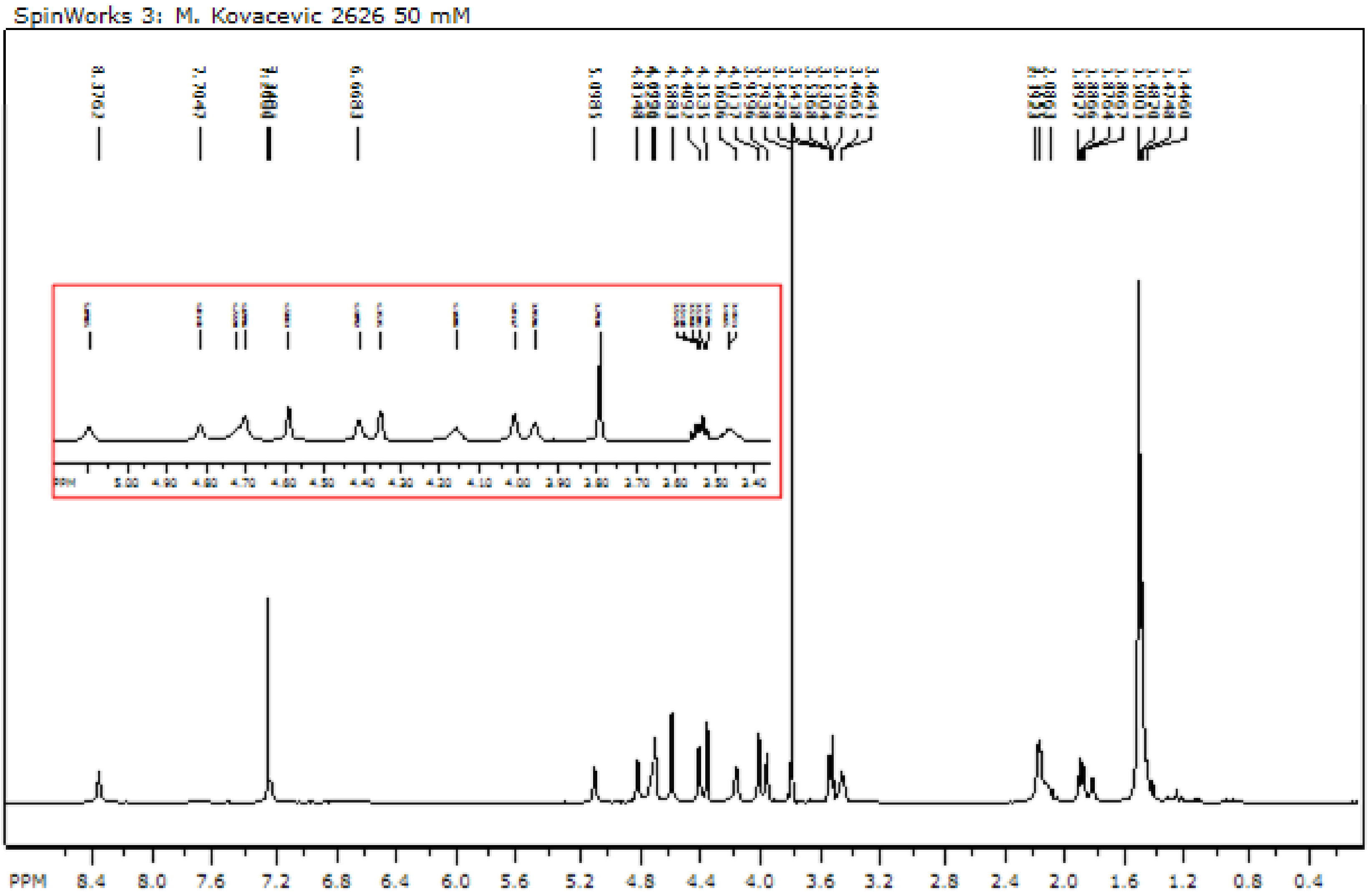

Figure S4. ^{1}H NMR spectrum of compound 2 ($c = 5 \times 10^{-2}$ M).

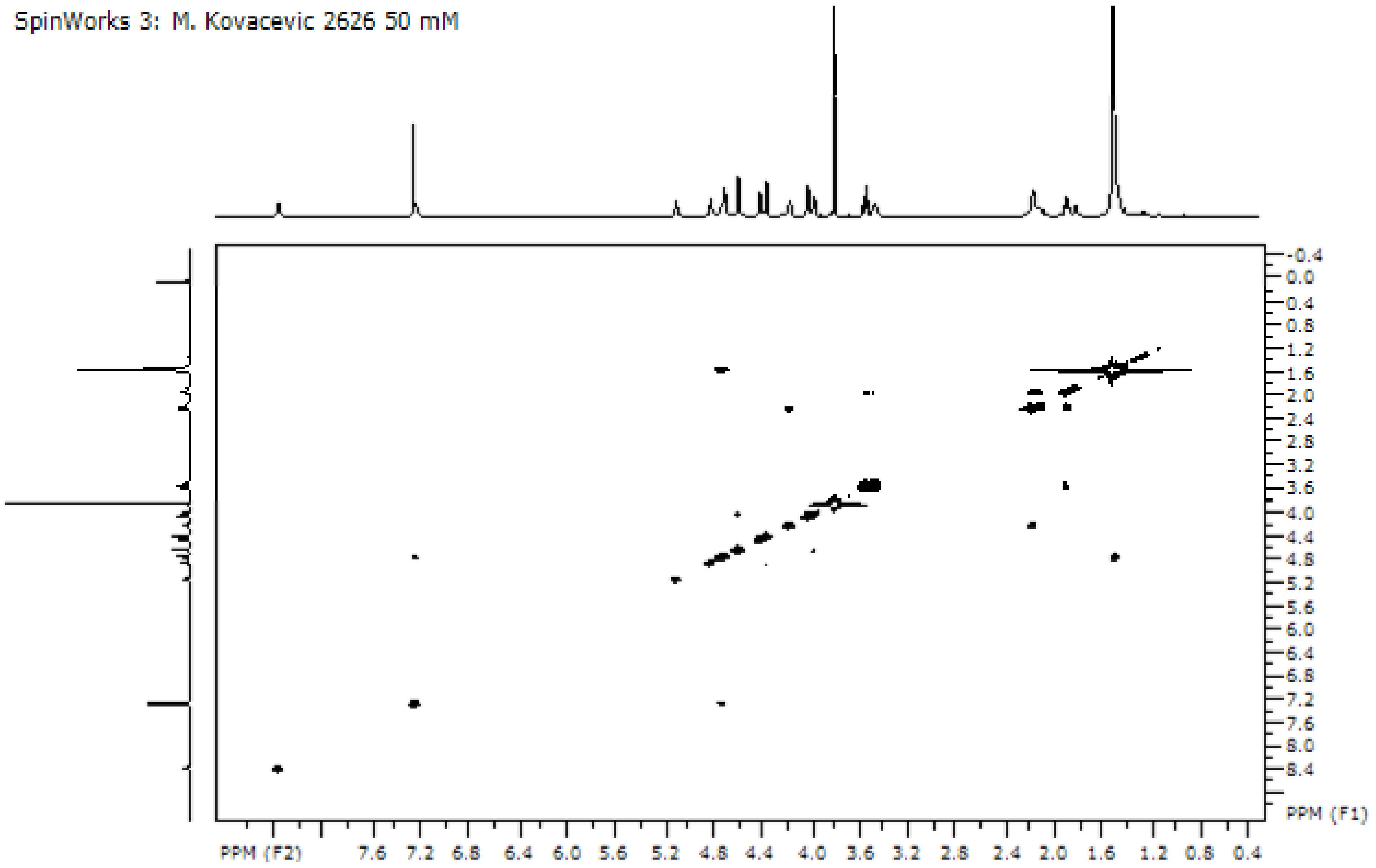

Figure S5. ^{1}H-^{1}H COSY NMR spectrum of compound 2 ($c = 5 \times 10^{-2}$ M).

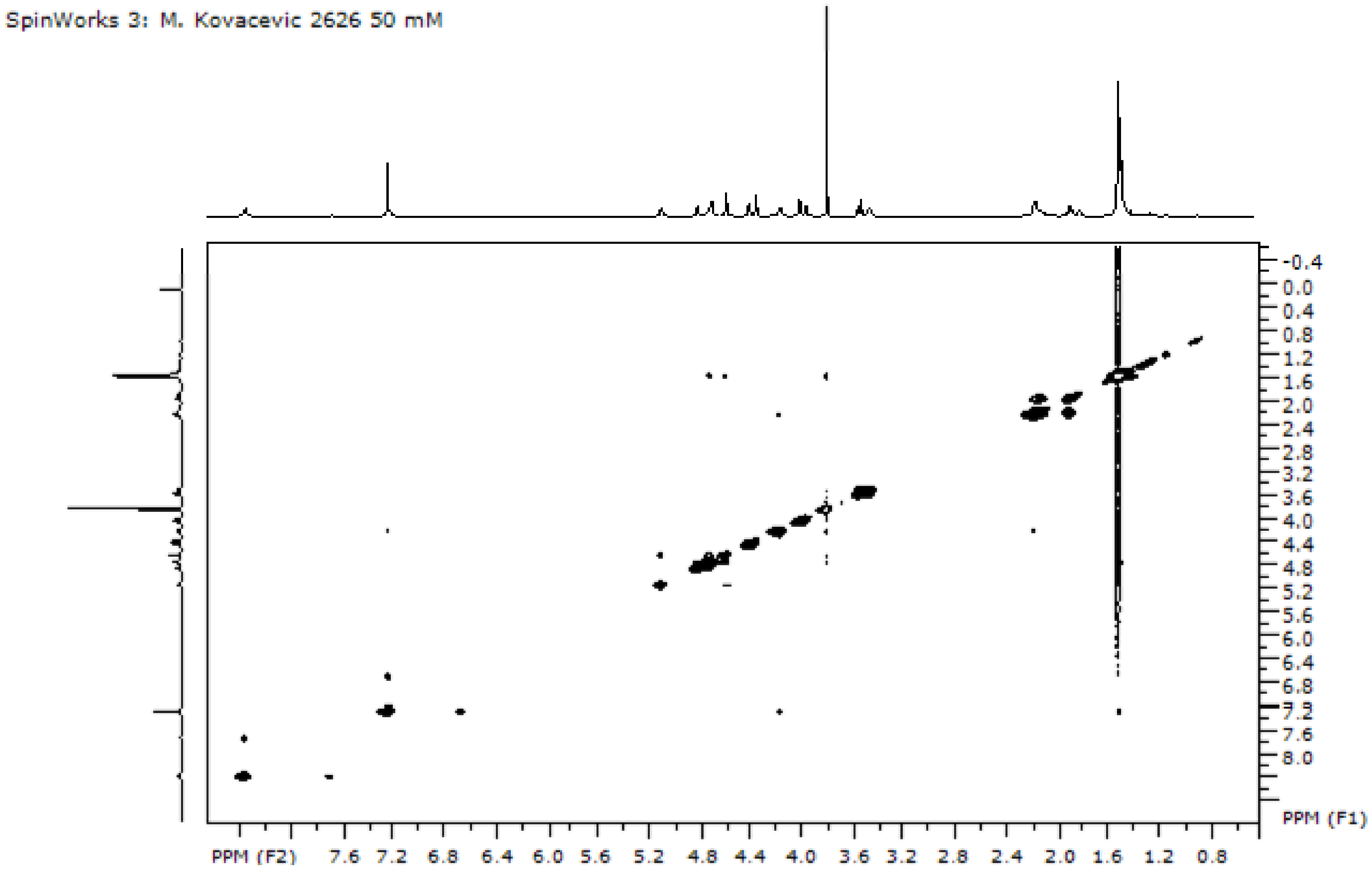

Figure S6. ^{1}H-^{1}H NOESY NMR spectrum of compound 2 ($c = 5 \times 10^{-2}$ M).

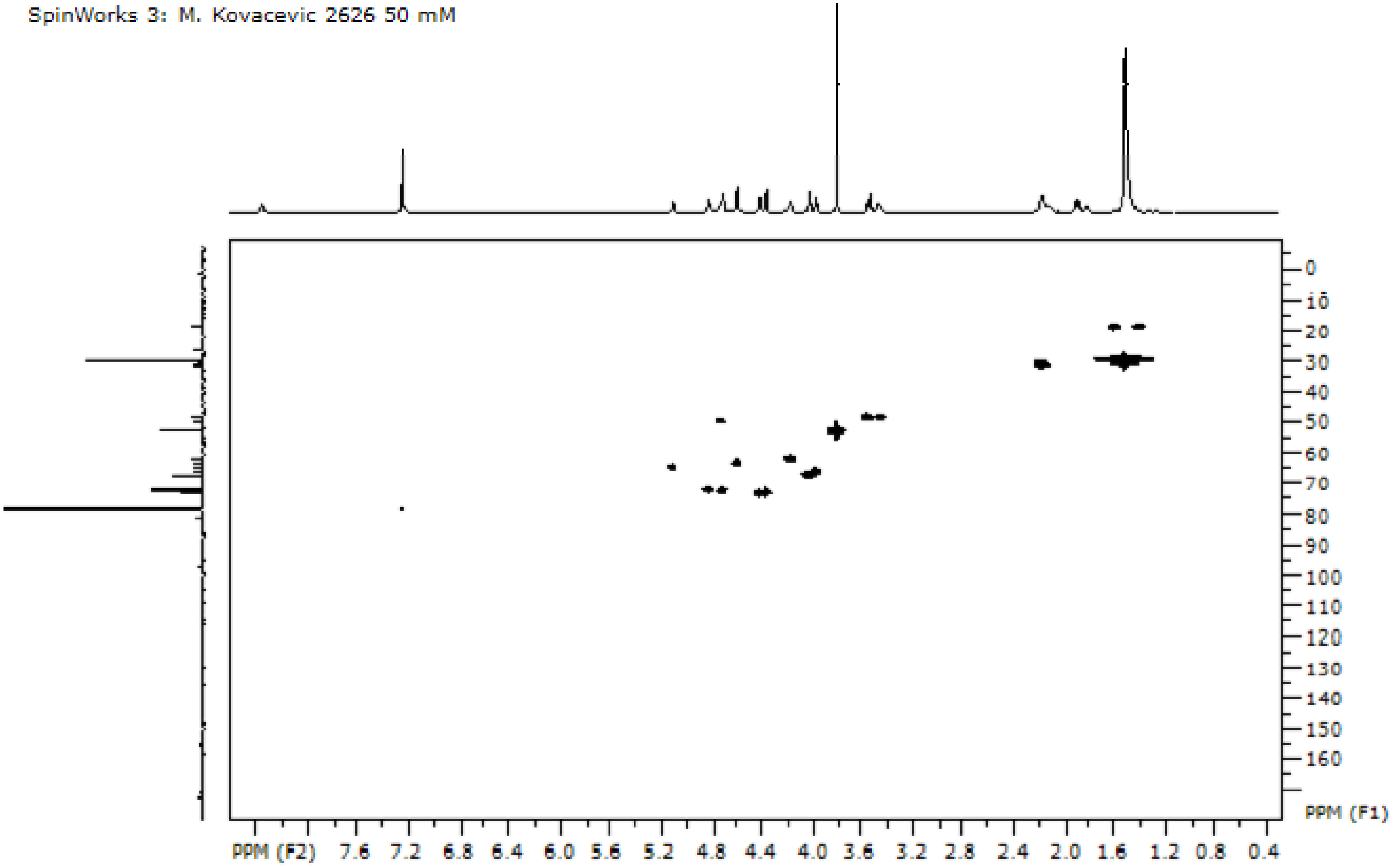

Figure S7. ^{1}H-^{13}C HMQC spectrum of compound 2 ($c = 5 \times 10^{-2}$ M).

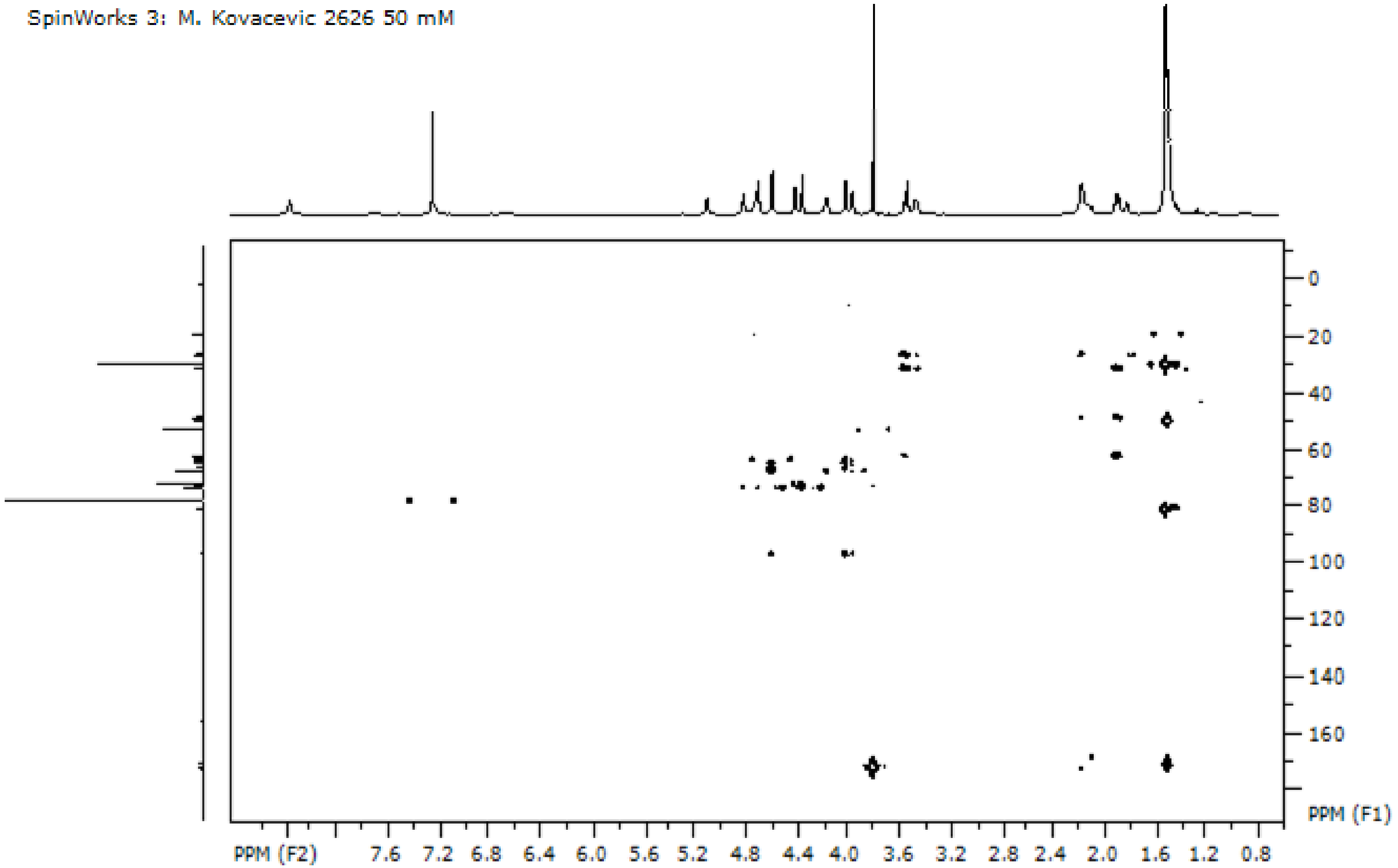

Figure S8. ^{1}H-^{13}C HMBC spectrum of compound 2 ($c = 5 \times 10^{-2}$ M).

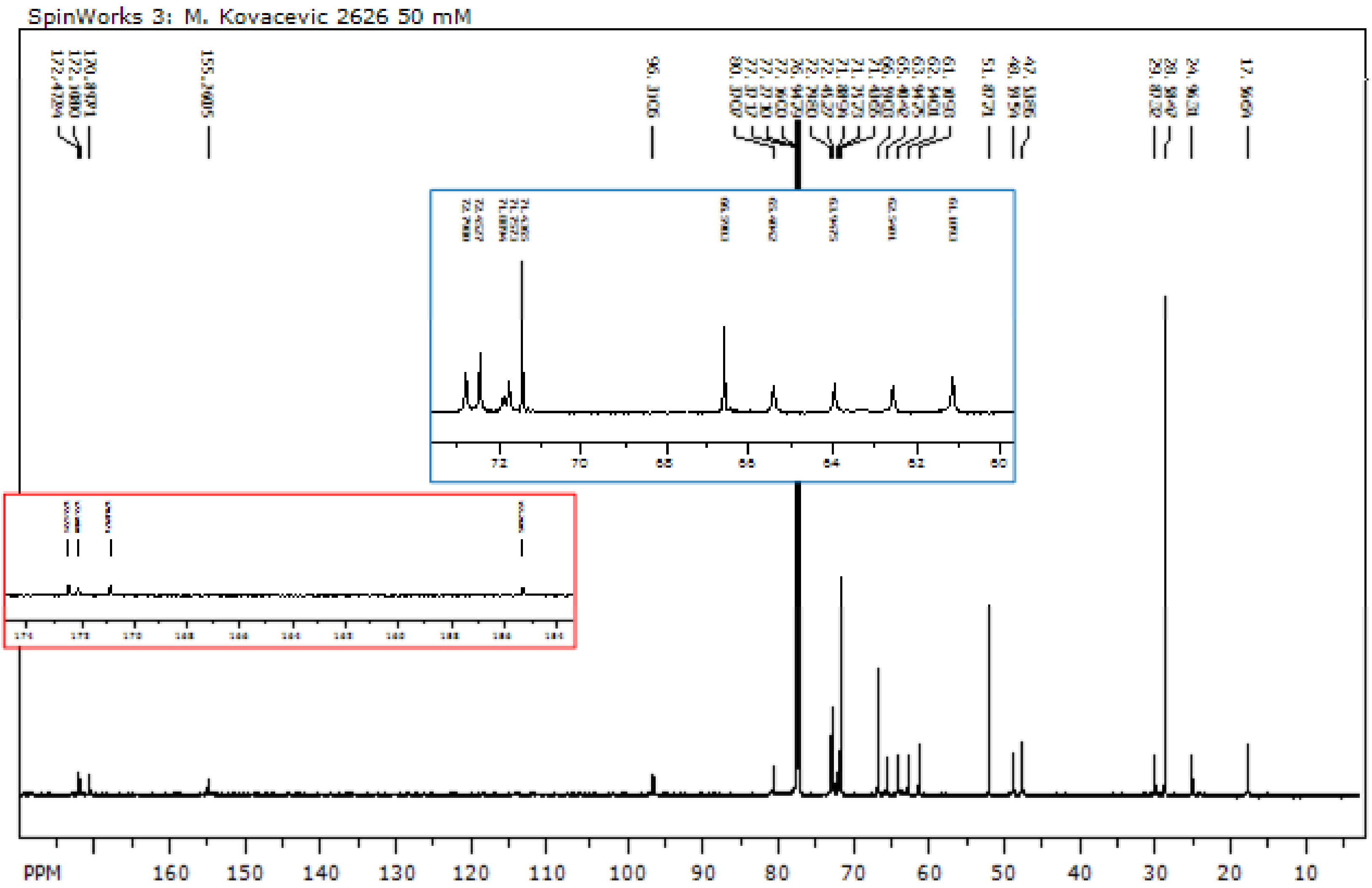

Figure S9. $^{13}C\{^{1}H\}$ NMR spectrum of compound 2 ($c = 5 \times 10^{-2}$ M).

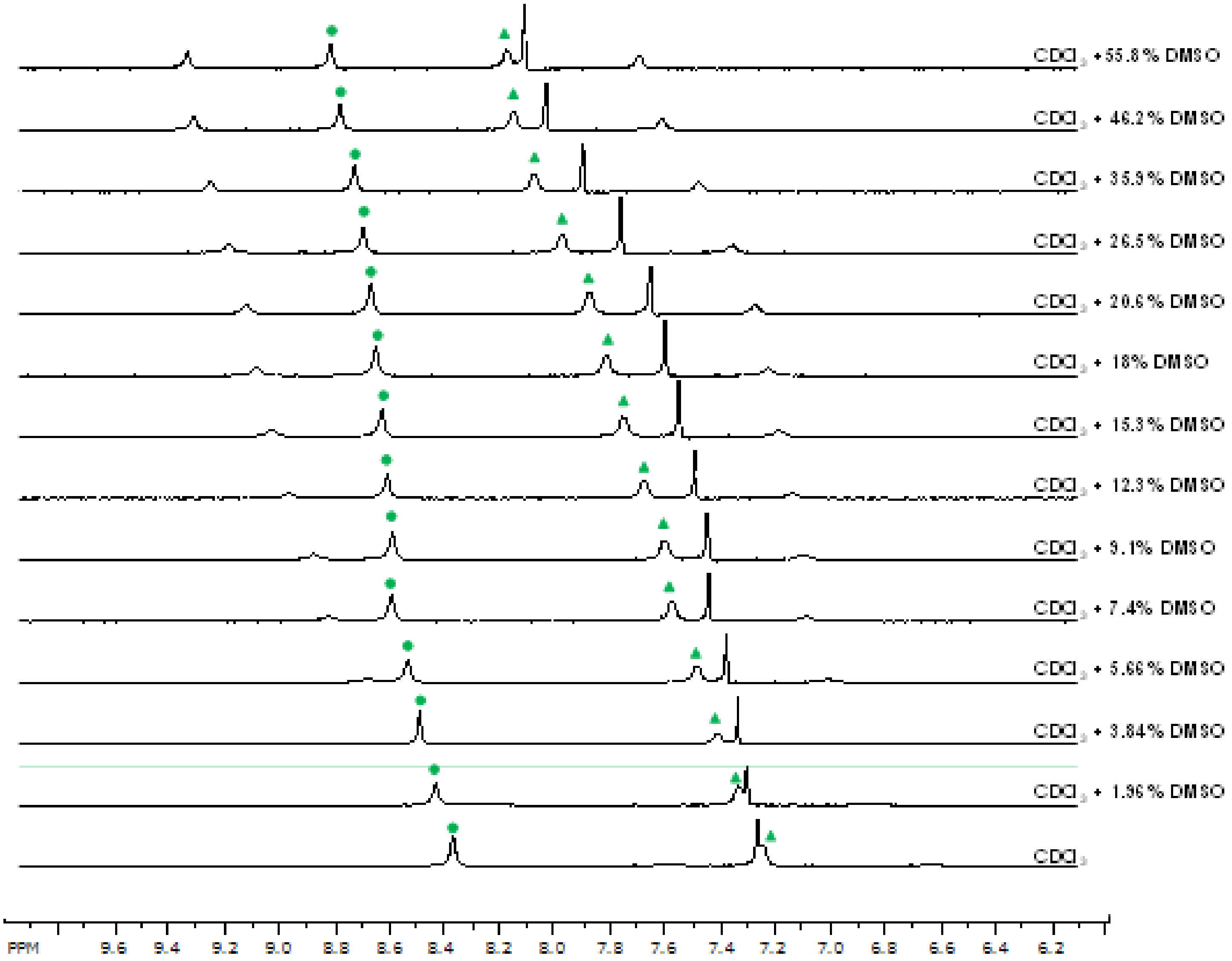

Figure S10. Solvent dependence of NH chemical shifts of compound 2 at varying concentrations of DMSO in $CDCl_3$ (c = 2.5 × 10-2 M).

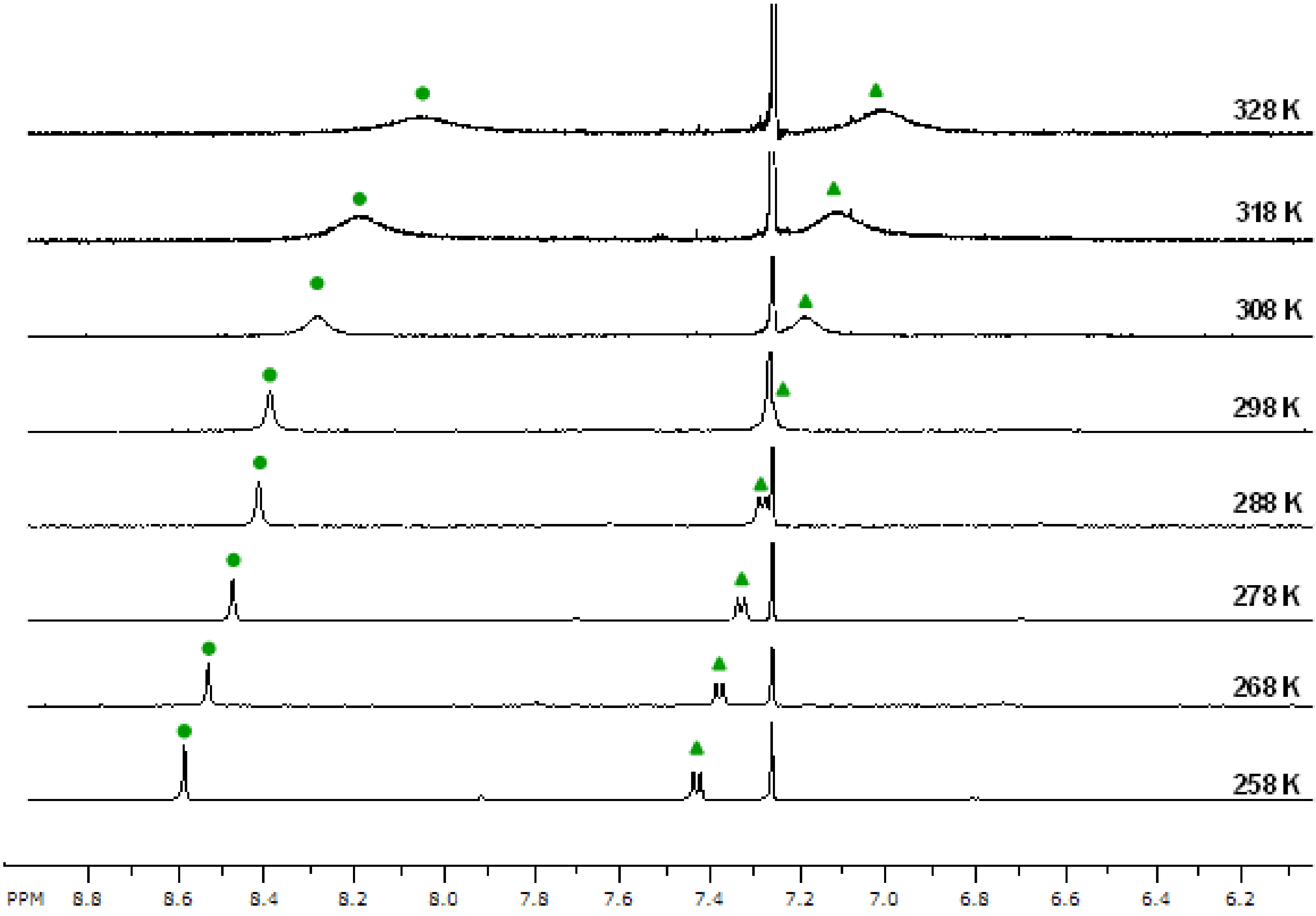

Figure S11. Temperature-dependent NH chemical shifts of compound 2 ($c = 1 \times 10^{-2}$ M).

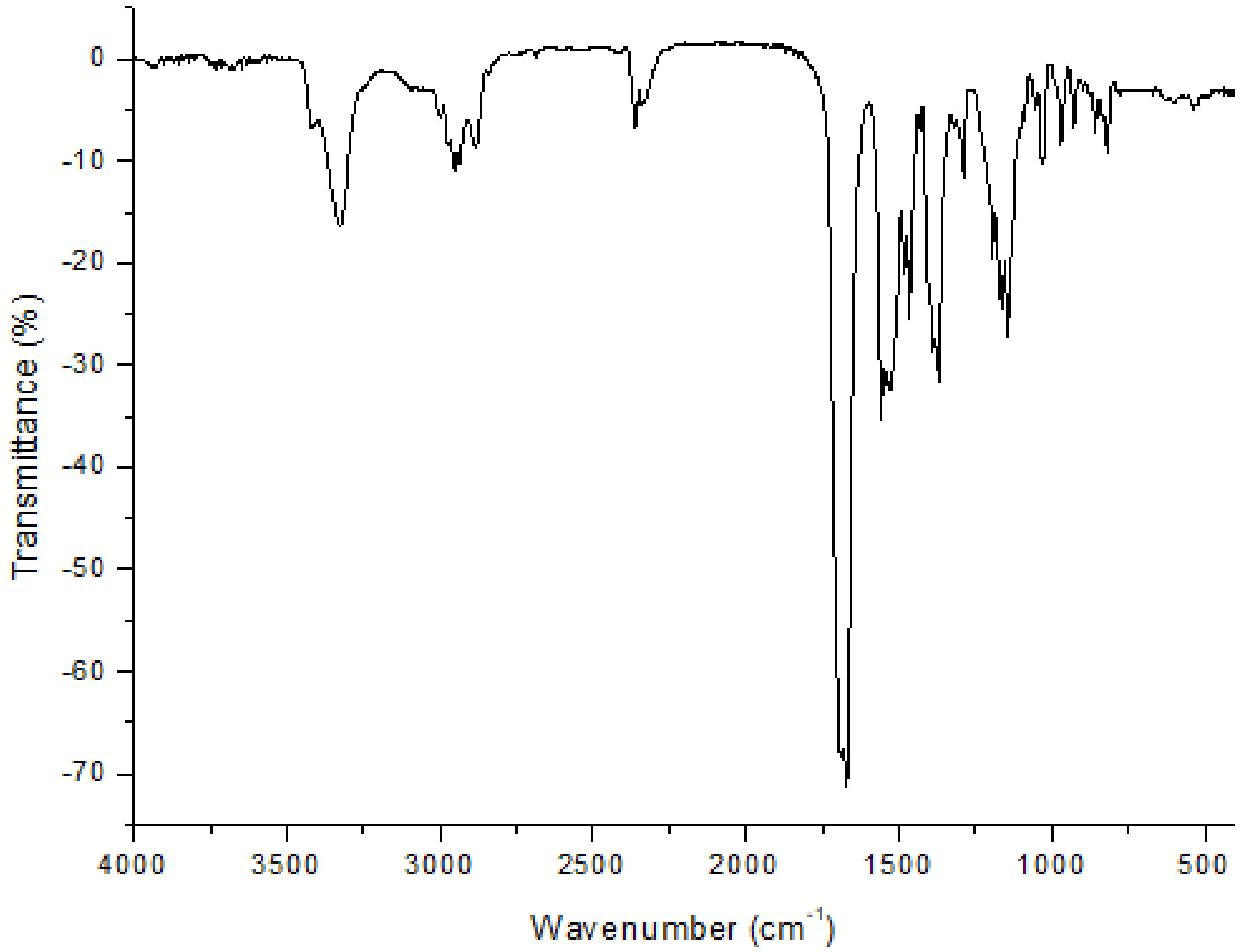

Figure S12. IR spectrum of compound 2 ($c = 5 \times 10^{-2}$ M) in DCM.

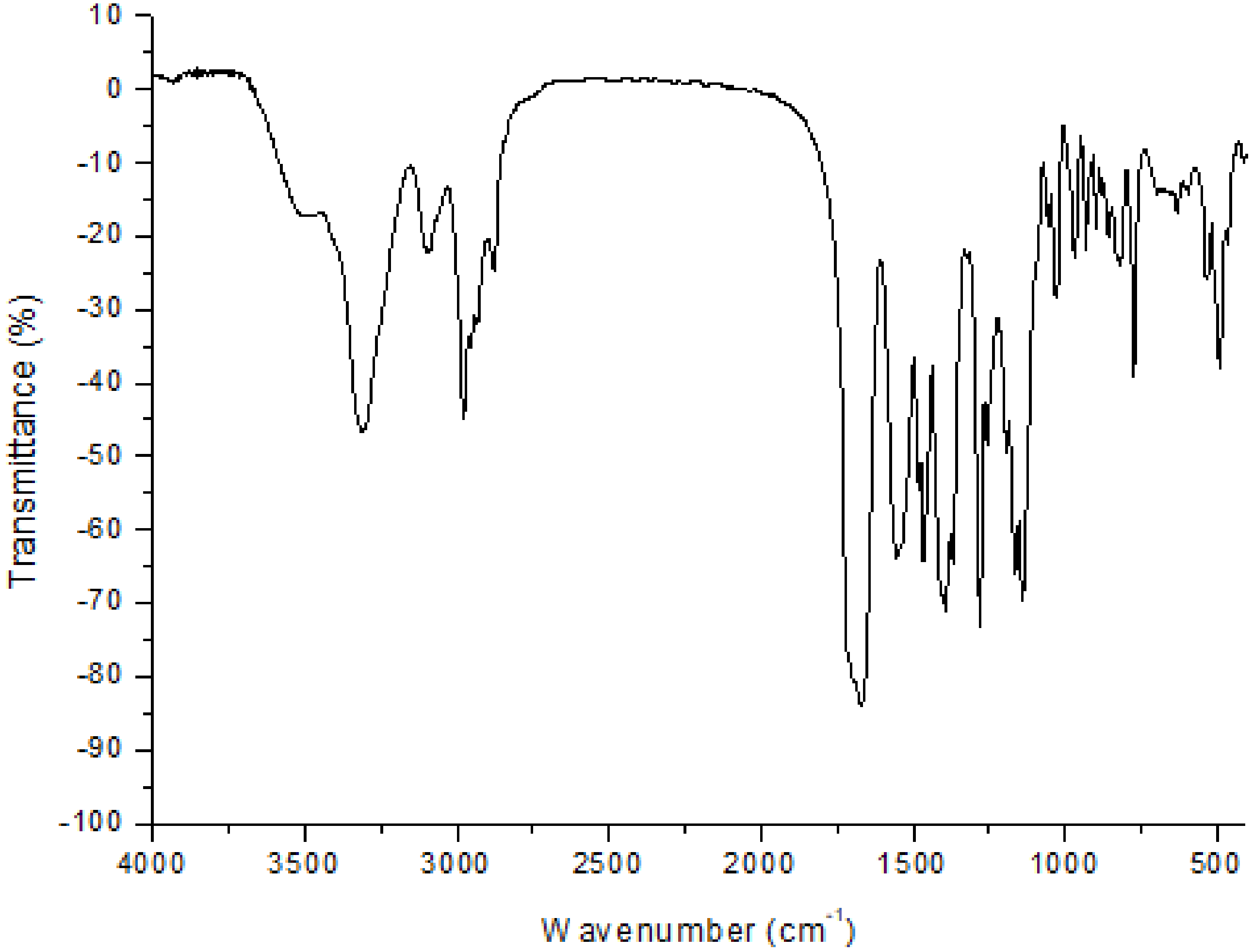

Figure S13. IR spectrum of compound 2 (2 mg) in KBr (200 mg).

Ac-D-Pro-L-Ala-NH-Fn-COOMe (3)

Ion type	Calc. mass	Measured mass	Mass error / ppm	Mol. Formula	Int. CAL
M+	469.1300	469.1280	4.5	$C_{22}H_{27}N_3O5Fe$	azitromicin

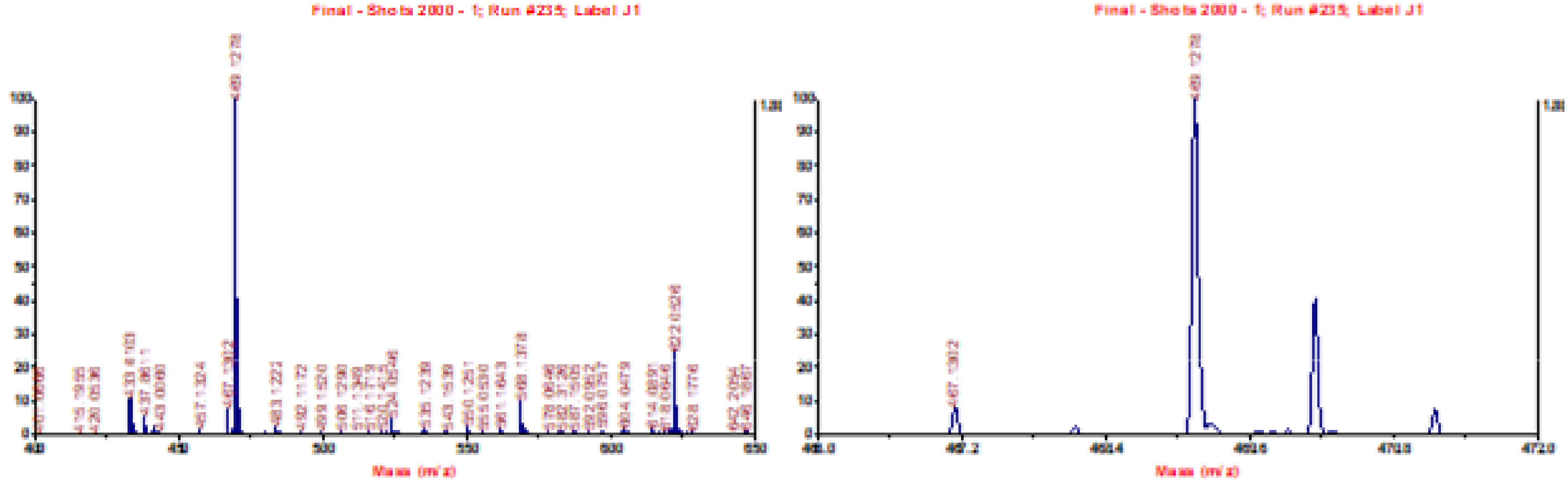

Figure S14. HRMS spectrum of compound 3.

Qualitative Analysis Report

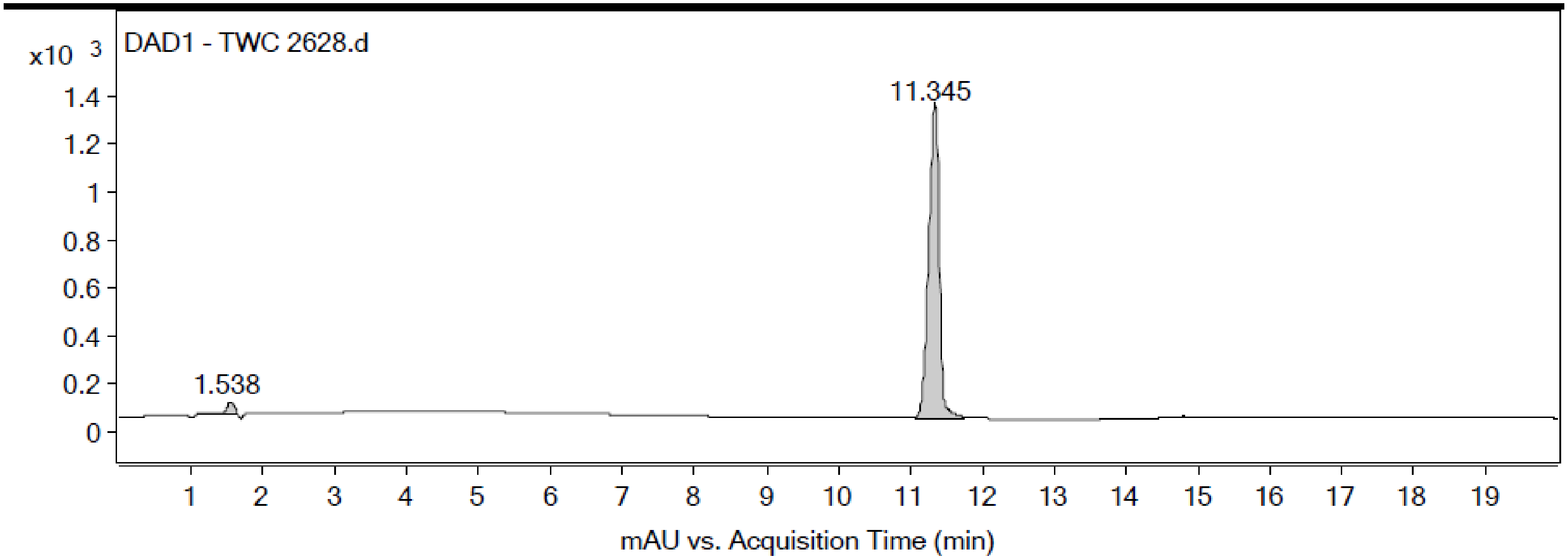

Integration Peak List

Peak	Start	RT	End	Height	Area	Area %
1	1.091	1.538	1.649	49.75	497.3	3.62
2	11.065	11.345	11.751	1322.47	13733.02	100

Spectrum Source	Fragmentor Voltage	Collision Energy	Ionization Mode
Peak (1) in "+/- TIC Scan"		0	ESI

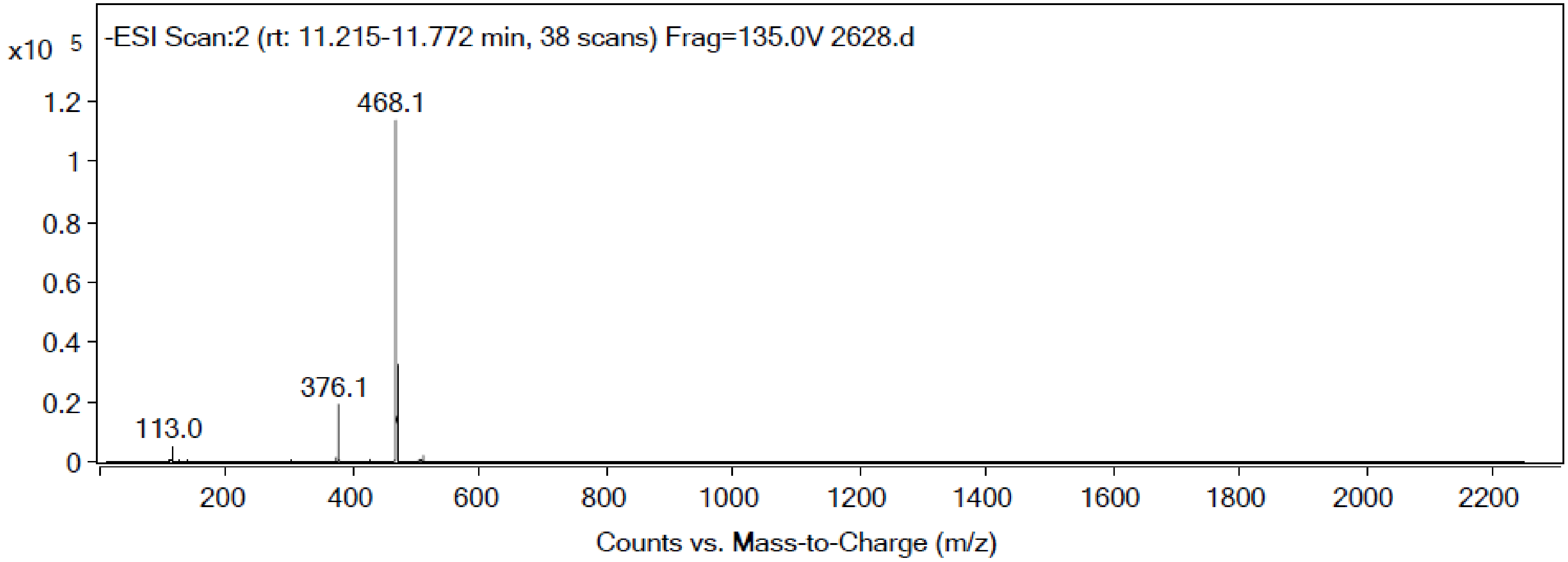

Peak List

m/z	z	Abund
113		5043.04
374		1191.55
376.1	1	19153.22
377	1	3753.97
466.1		7309.16
468.1	1	113881.13
469.1	1	32806.95
470.1	1	5590.01

Figure S15. HPLC-ESI spectra of compound **3.**

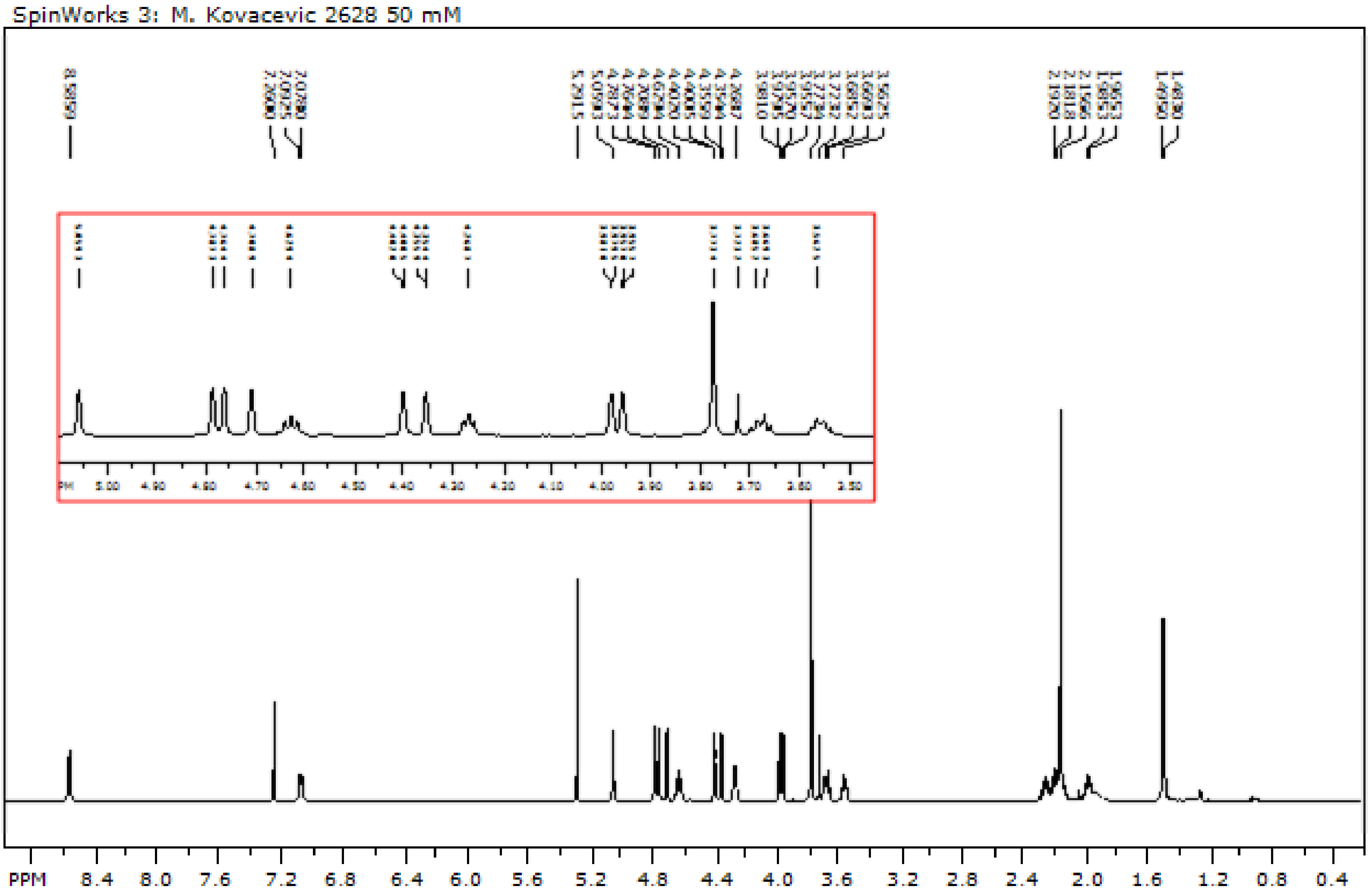

Figure S16. ^{1}H NMR spectrum of compound 3 ($c = 5 \times 10^{-2}$ M).

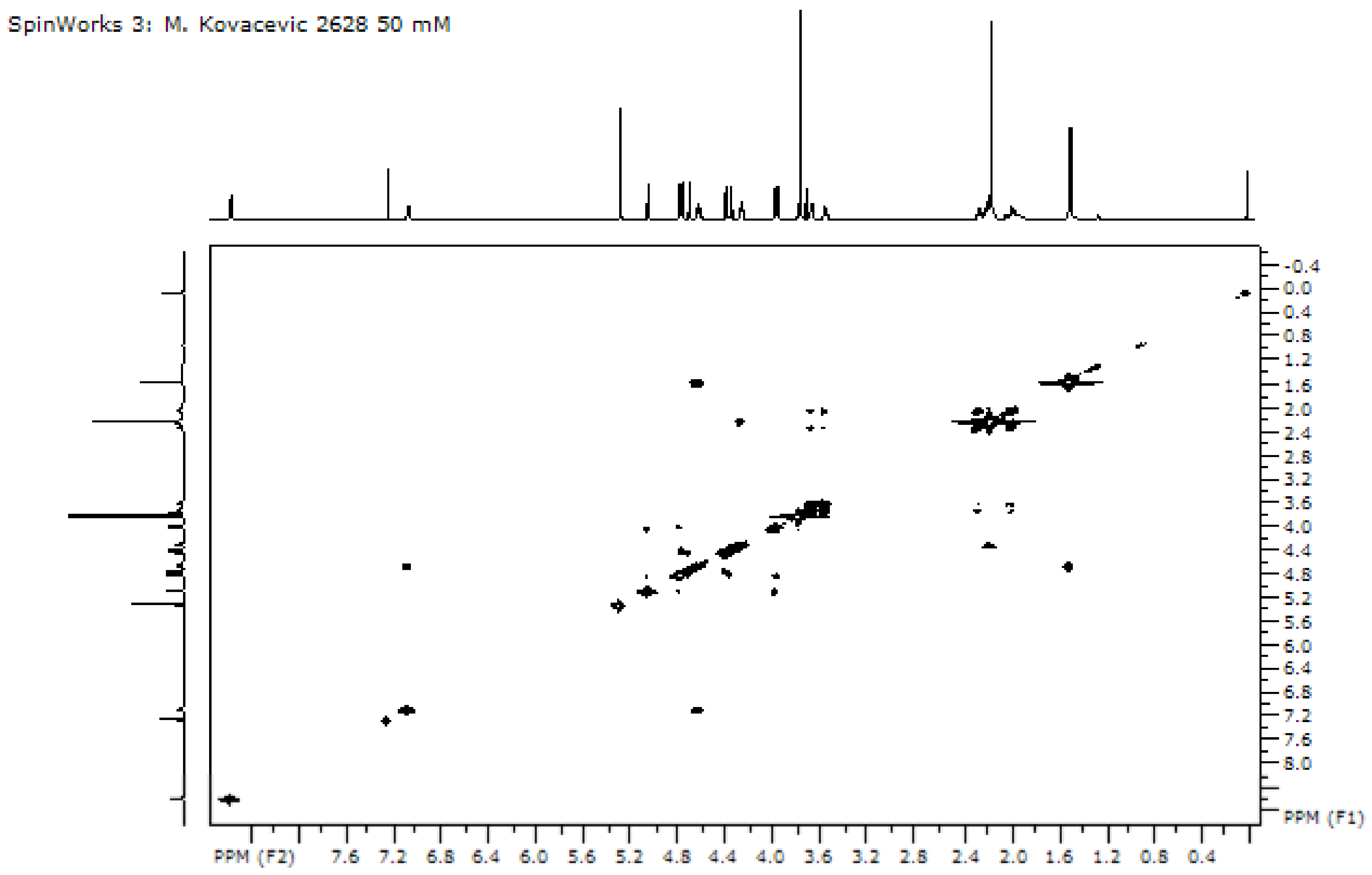

Figure S17. ^1H-^1H COSY NMR spectrum of compound 3 ($c = 5 \times 10^{-2}$ M).

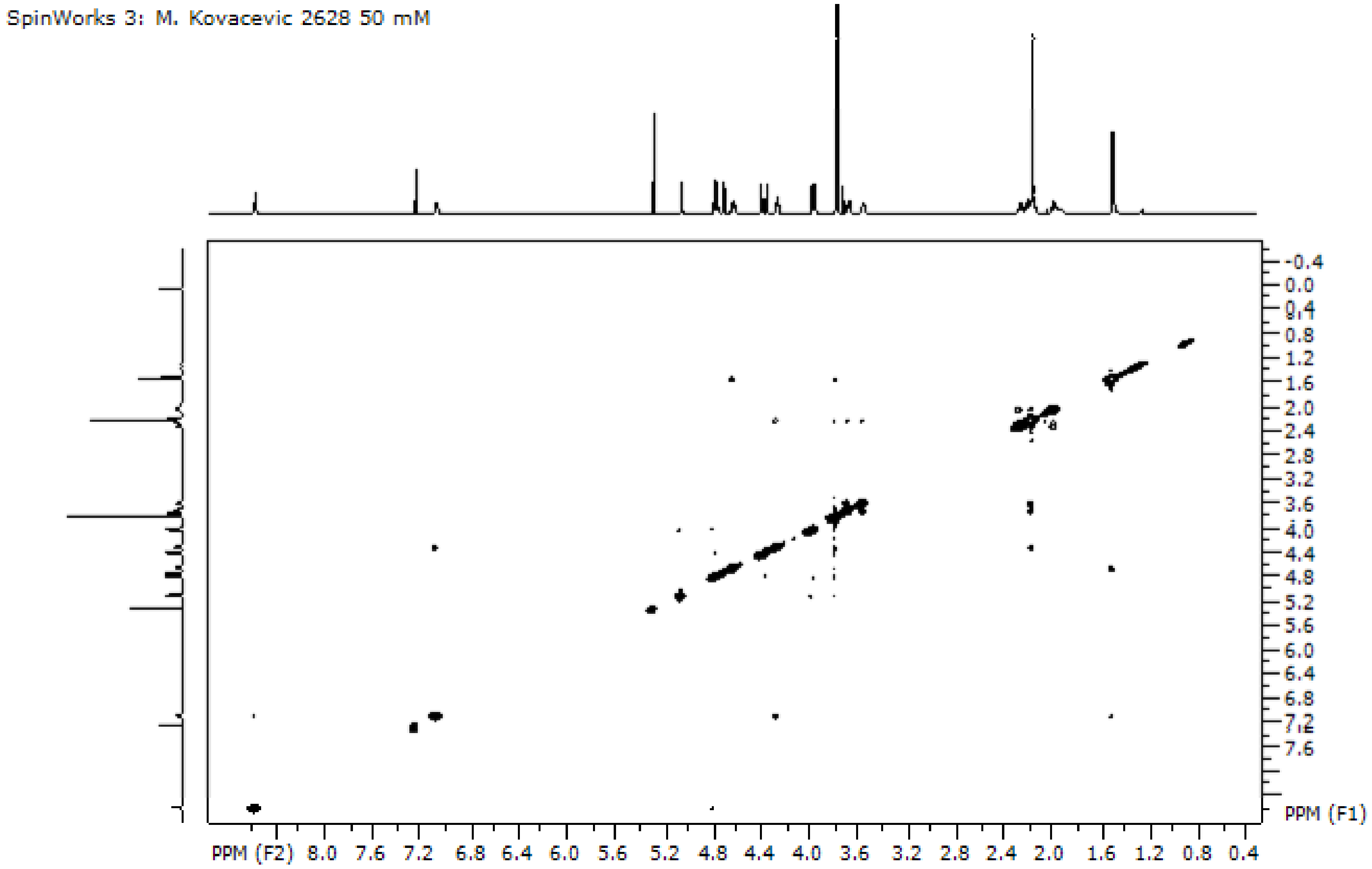

Figure S18. ^1H-^1H NOESY NMR spectrum of compound 3 ($c = 5 \times 10^{-2}$ M).

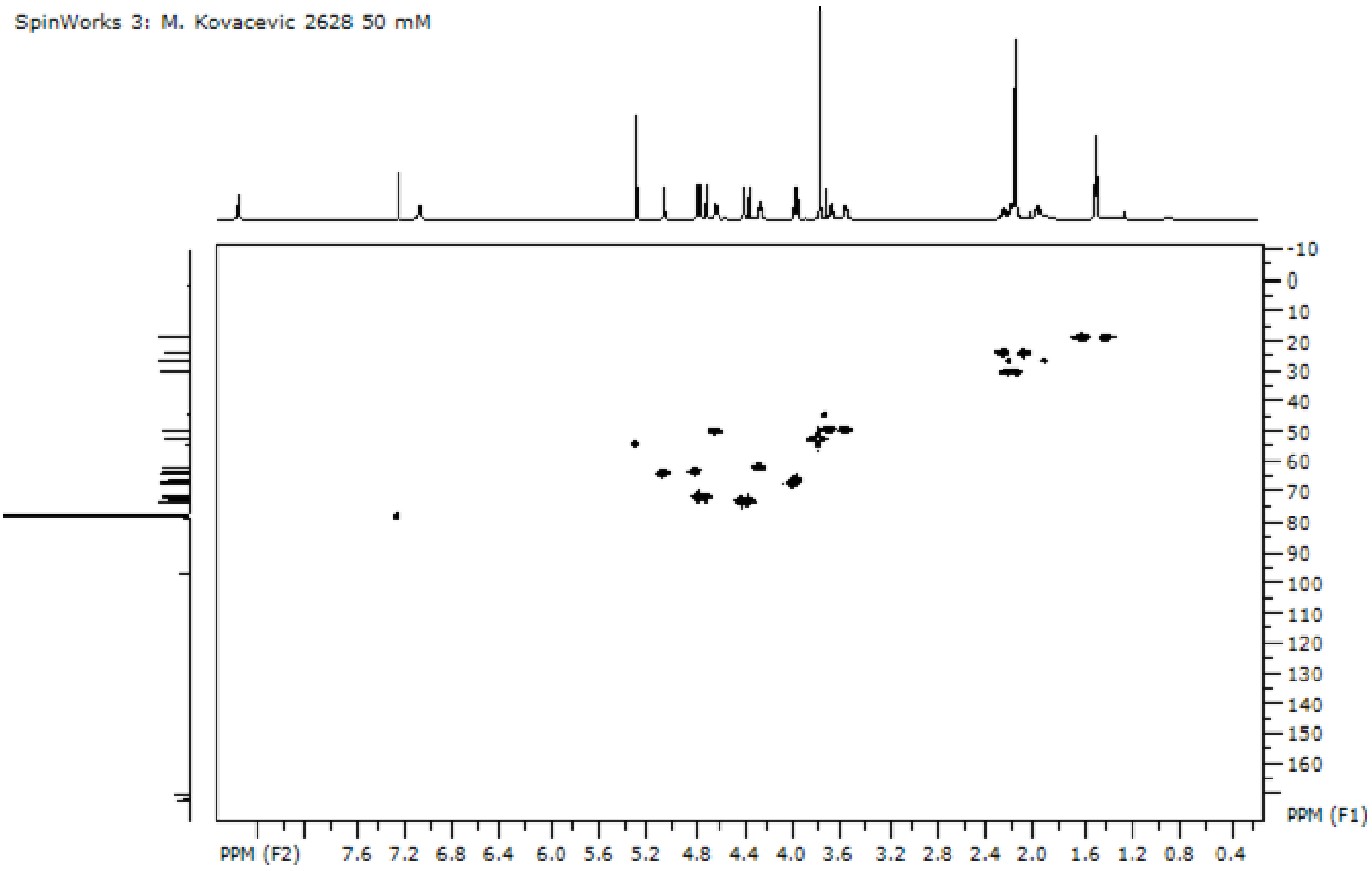

Figure S19. ^1H-^{13}C HMQC spectrum of compound 3 ($c = 5 \times 10^{-2}$ M).

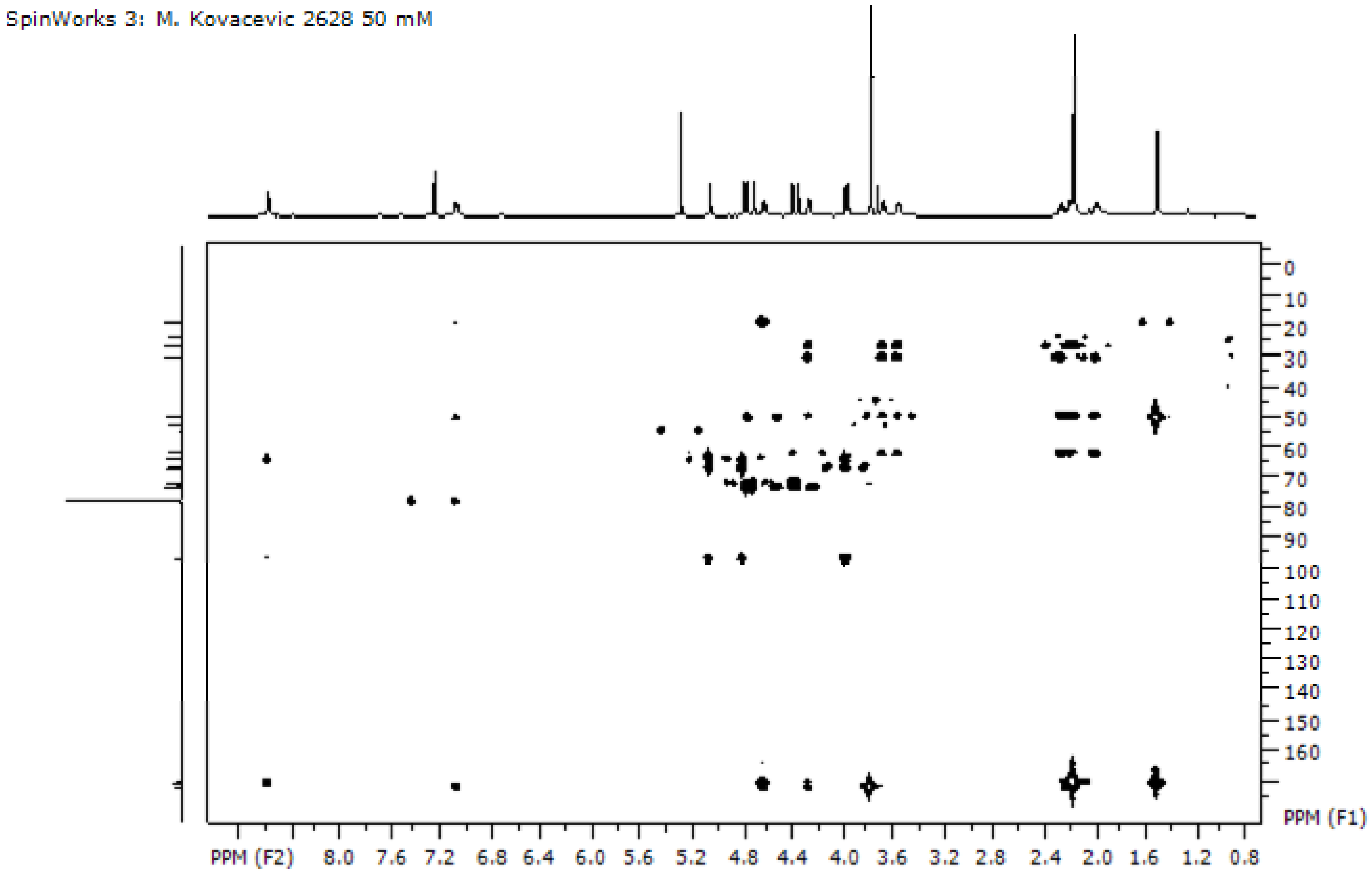

Figure S20. ^1H-^{13}C HMBC spectrum of compound 3 ($c = 5 \times 10^{-2}$ M).

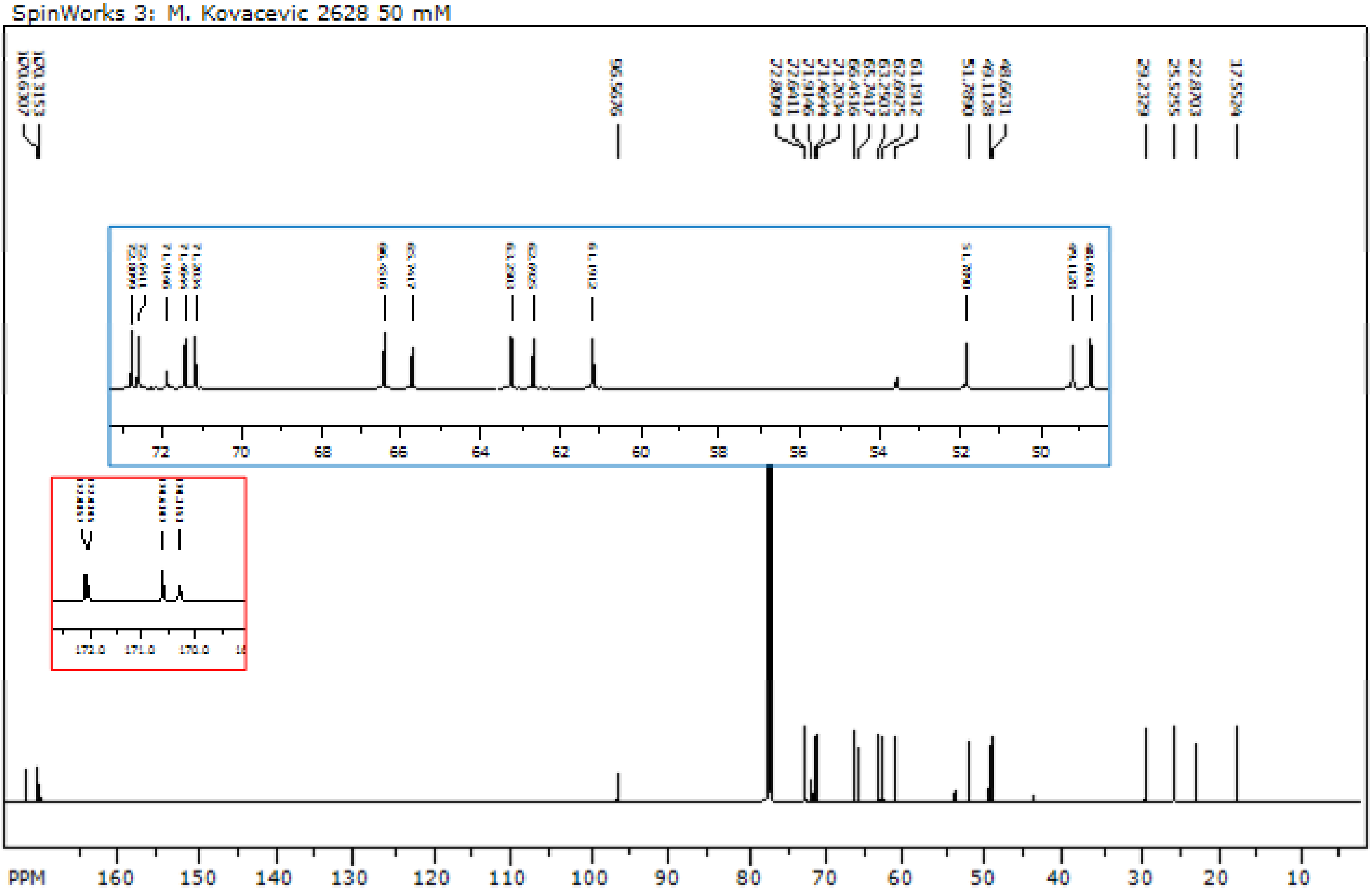

Figure S21. $^{13}C\{^1H\}$ NMR spectrum of compound 3 ($c = 5 \times 10^{-2}$ M).

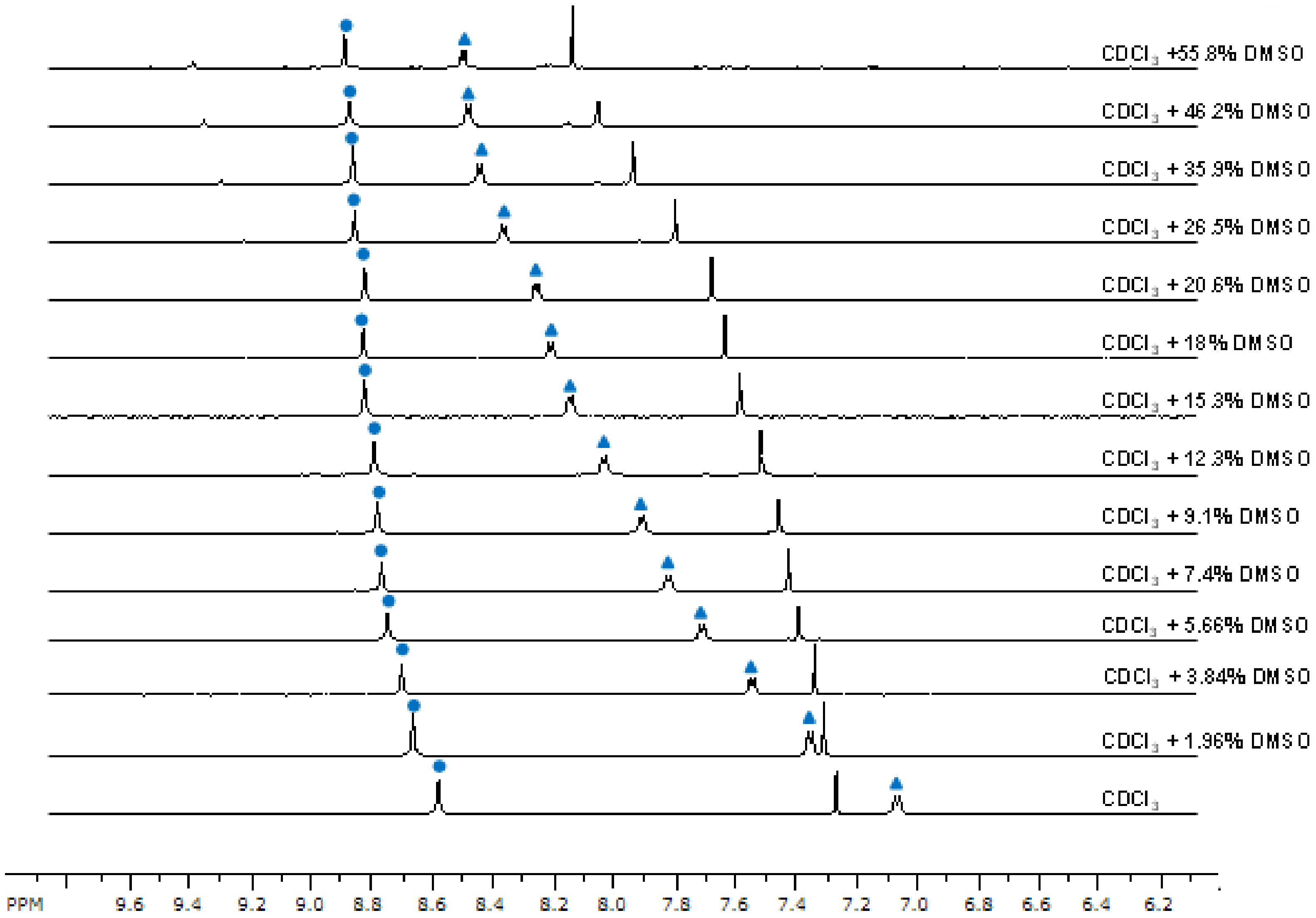

Figure S22. Solvent dependence of NH chemical shifts of compound 3 at varying concentrations of DMSO in $CDCl_3$ ($c = 2.5 \times 10^{-2}$ M).

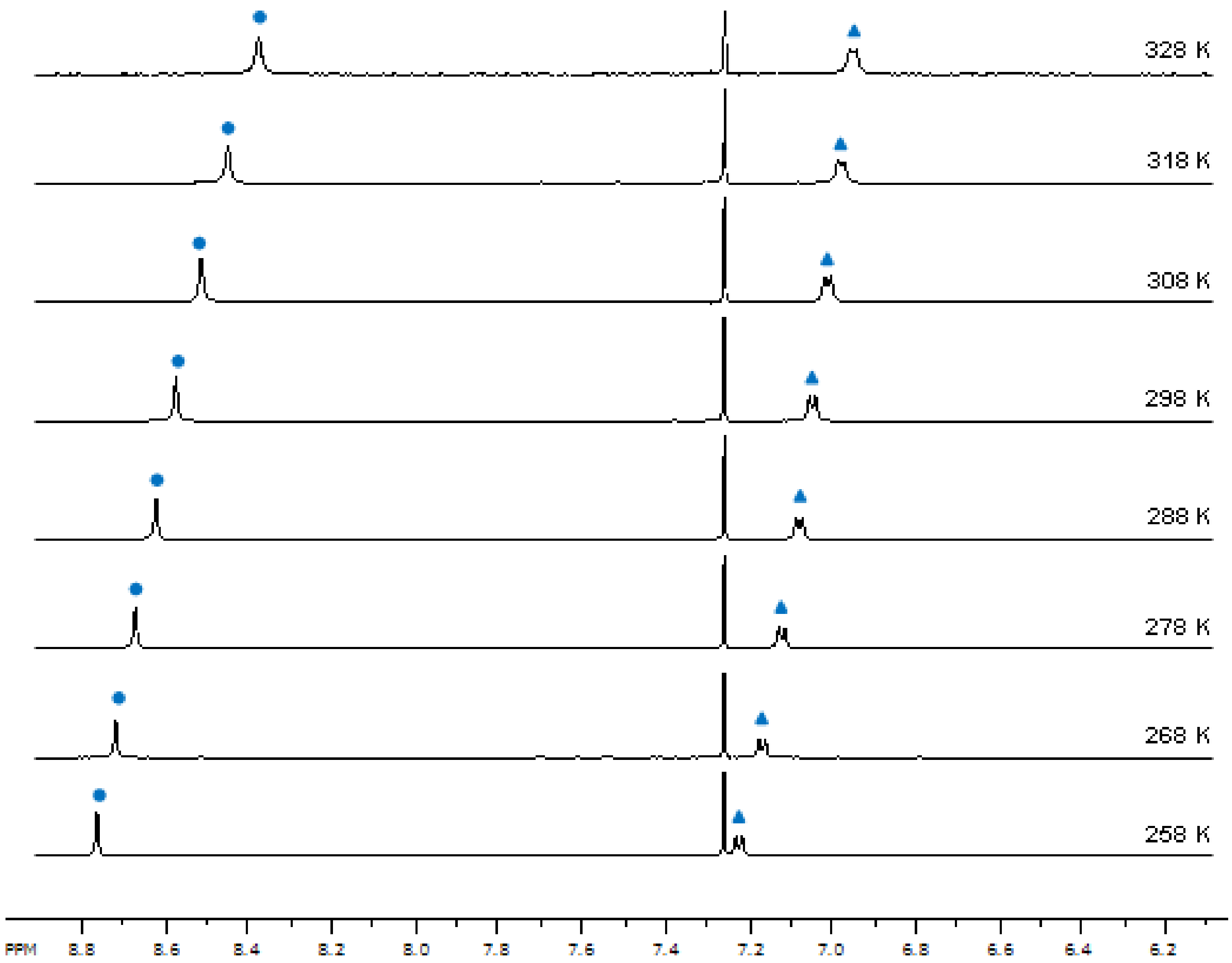

Figure S23. Temperature-dependent NH chemical shifts of compound 3 ($c = 1 \times 10^{-2}$ M).

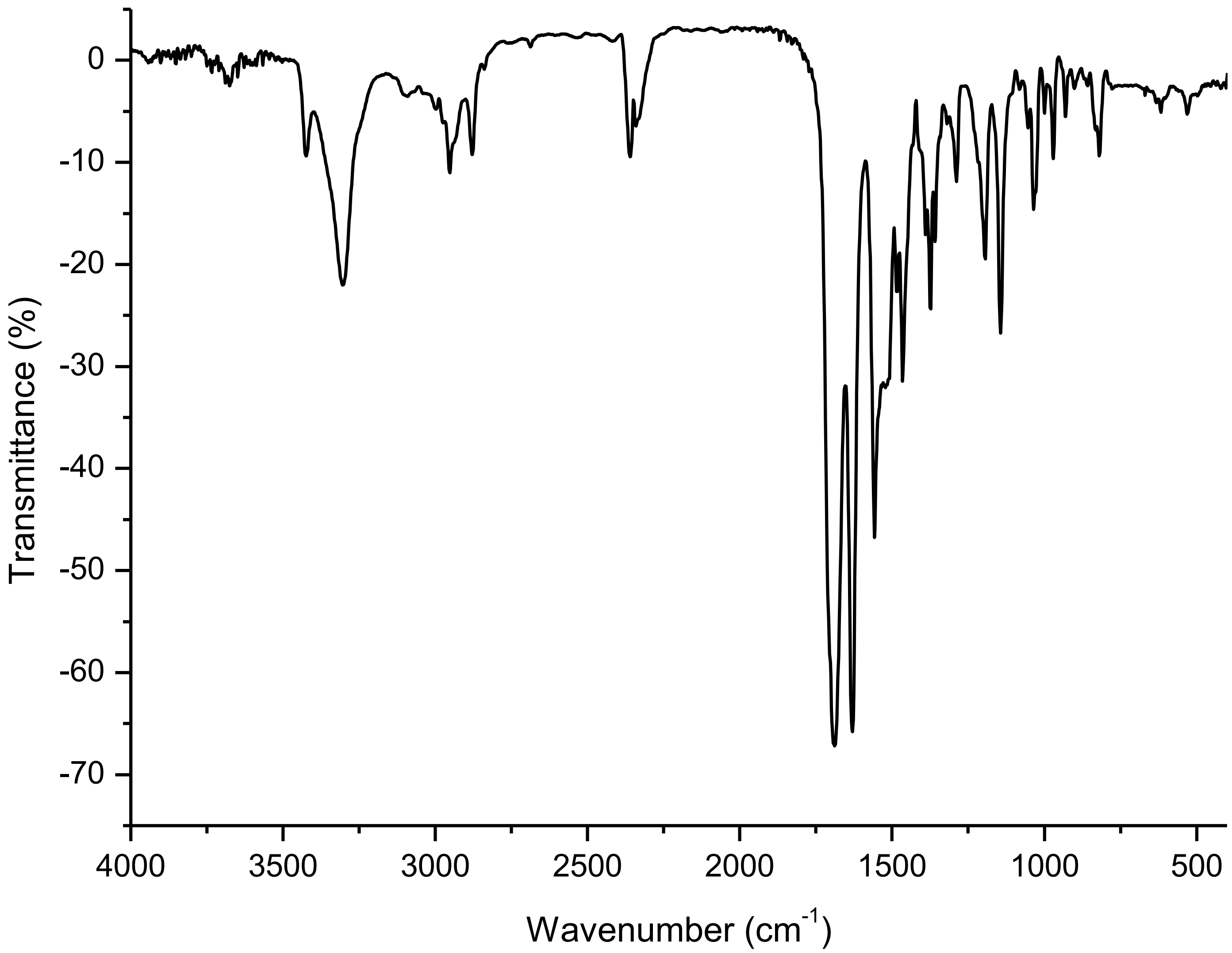

Figure S24. IR spectrum of compound **3** ($c = 5 \times 10^{-2}$ M) in DCM.

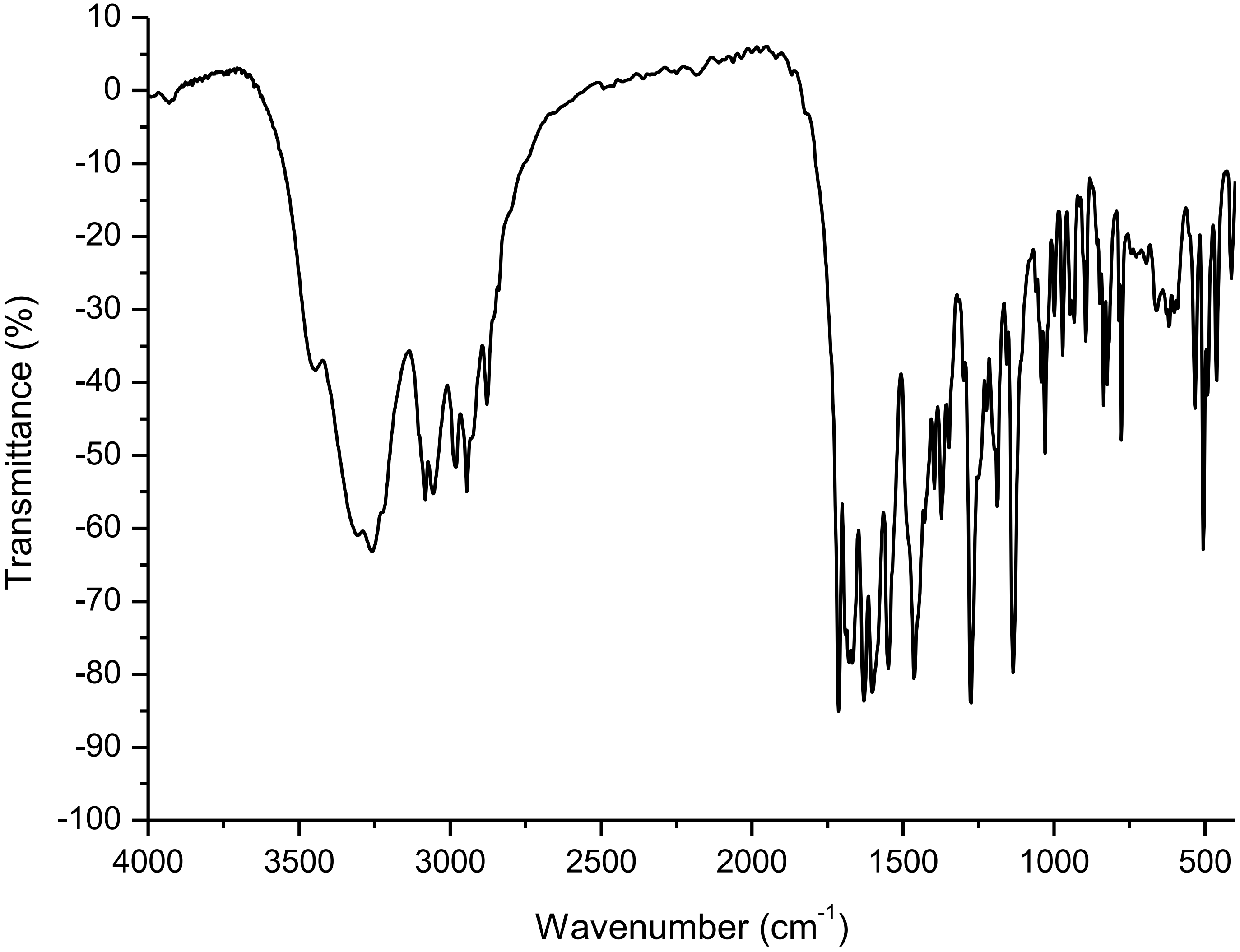

Figure S25. IR spectrum of compound **3** (2 mg) in KBr (200 mg).

Boc-L-Pro-L-Ala-NH-Fn-COOMe (4)

Ion type	Calc. mass	Measured mass	Mass error / ppm	Mol. Formula	Int. CAL
M+	527.1719	527.1729	1.9	$C_{25}H_{33}N_3O_6Fe$	azitromicin

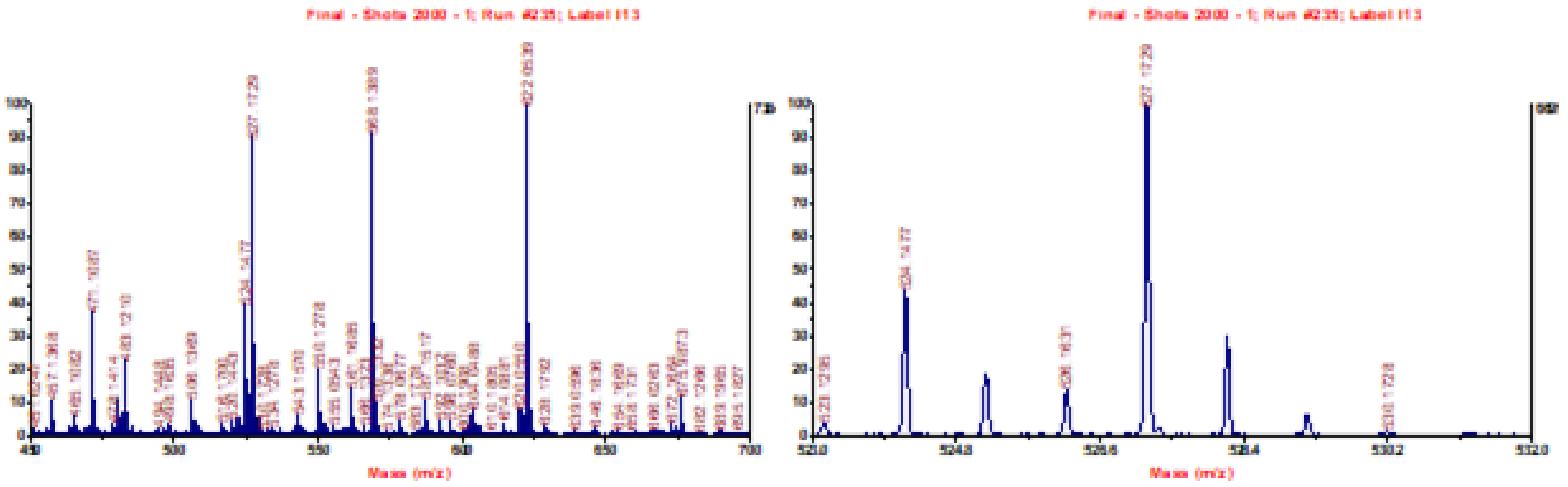

Figure S26. HRMS spectrum of compound **4.**

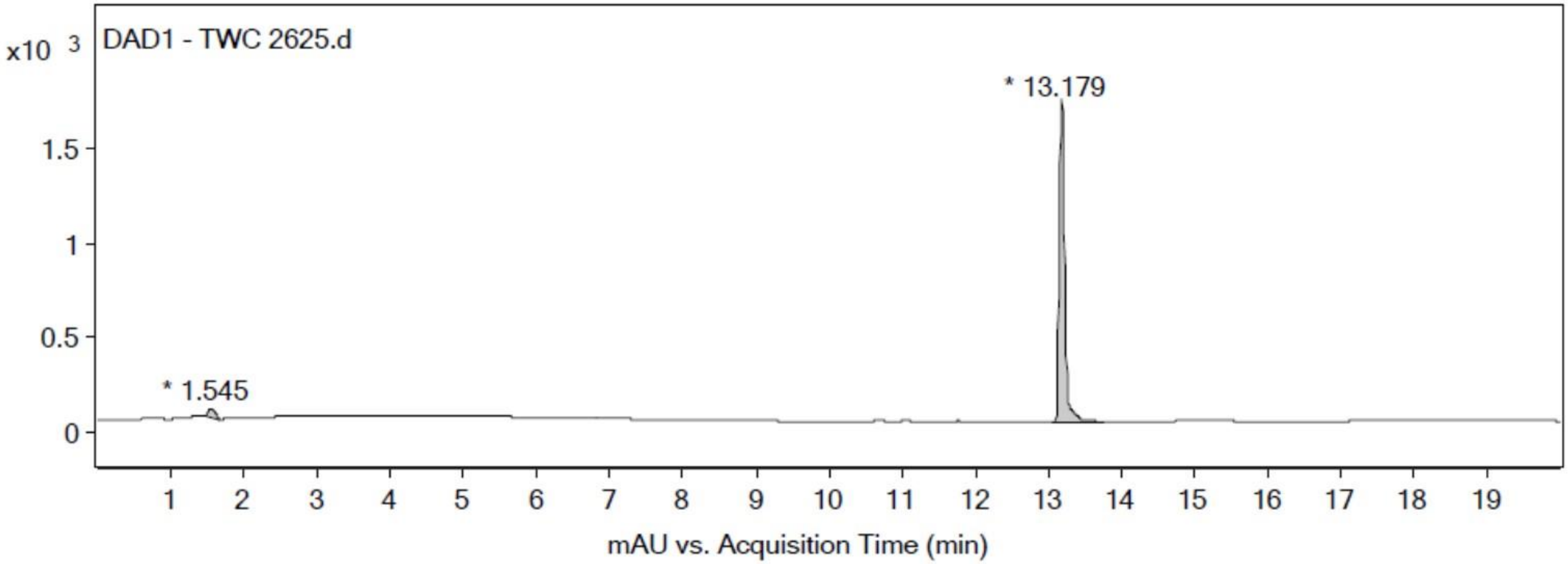

Integration Peak List

Peak	Start	RT	End	Height	Area	Area %
1	1.452	1.545	1.679	49.37	370.02	4.04
2	13.065	13.179	13.765	1702.41	9150.92	100

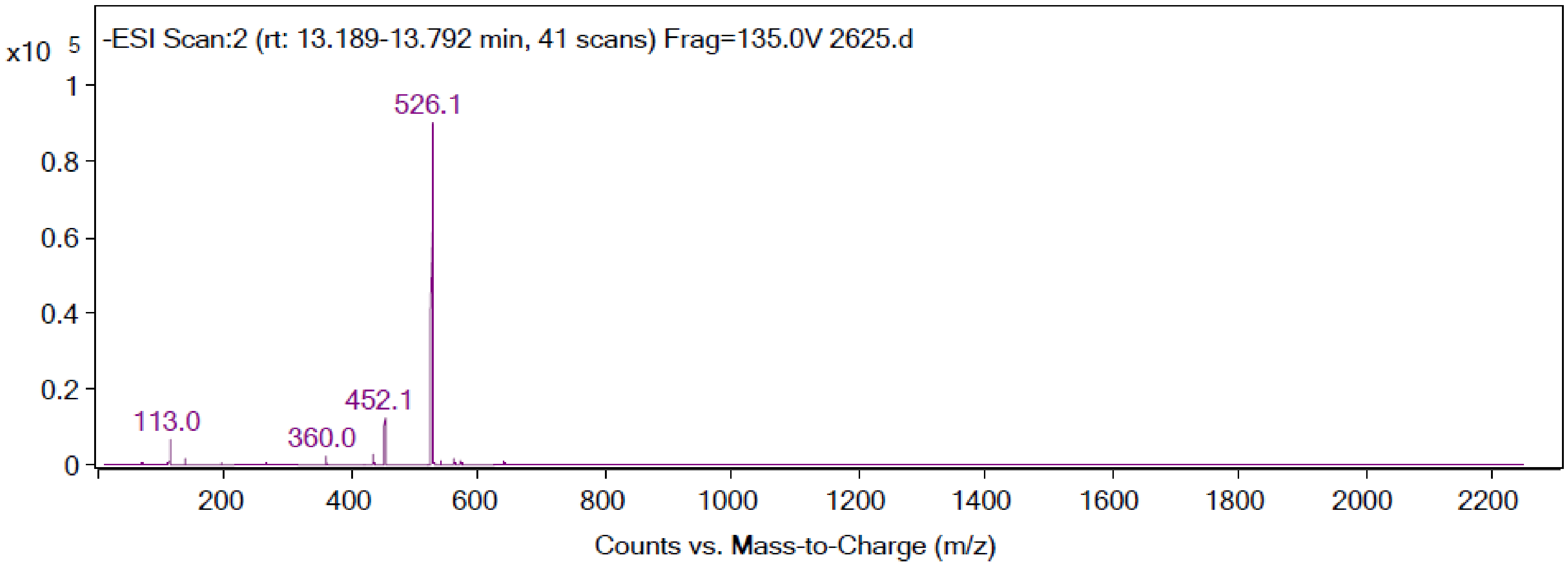

Peak List

m/z	z	Abund
113		6847.82
360		1990.32
434.1		2932.19
452.1	1	12574.58
453	1	3605.96
524.1		6158.28
526.1	1	90050.9
527.2	1	30406.55
528.1	1	6029.68
562		1833.22

Figure S27. HPLC-ESI spectra of compound **4.**

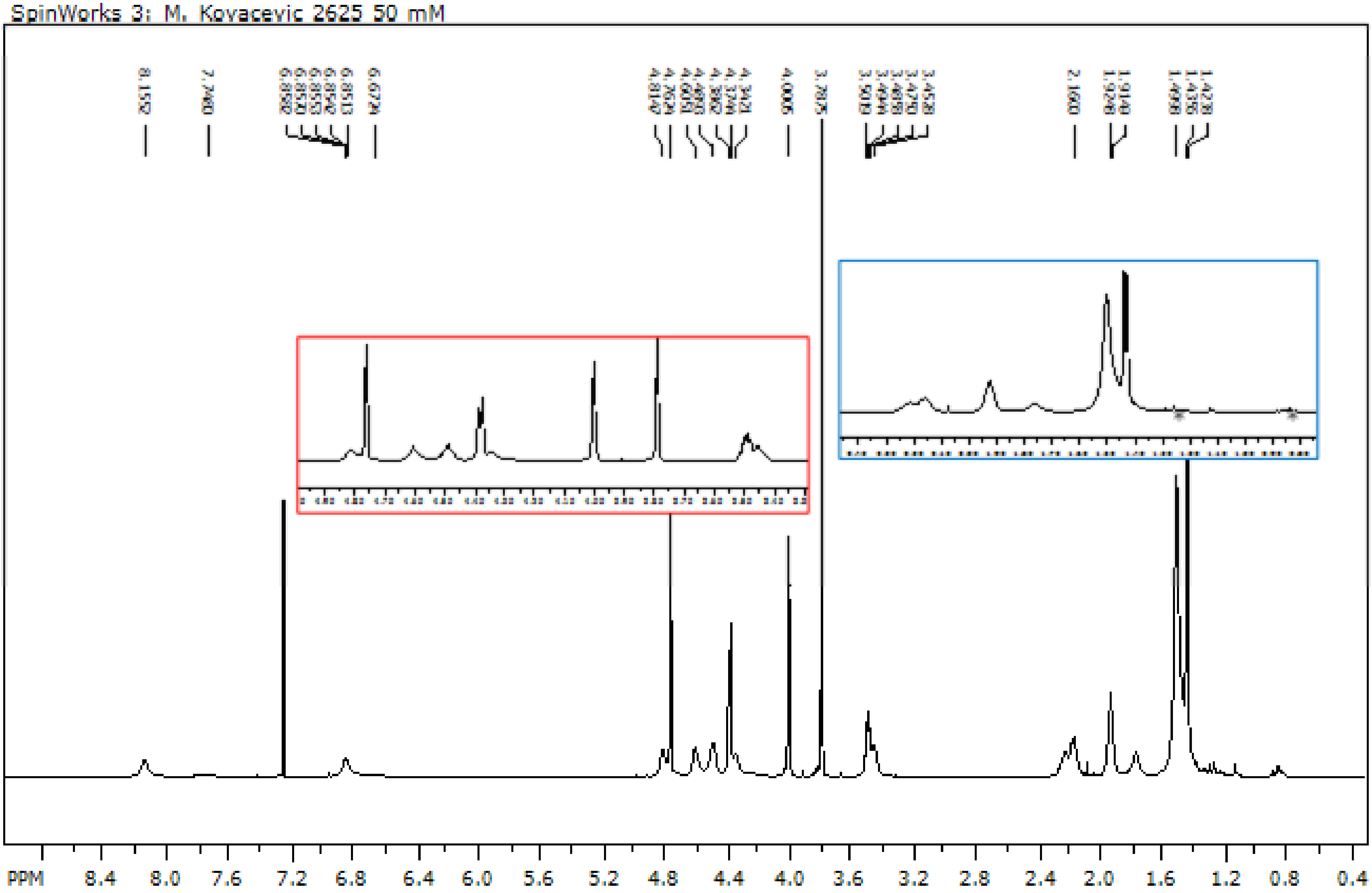

Figure S28. ¹H NMR spectrum of compound **4** ($c = 5 \times 10^{-2}$ M).

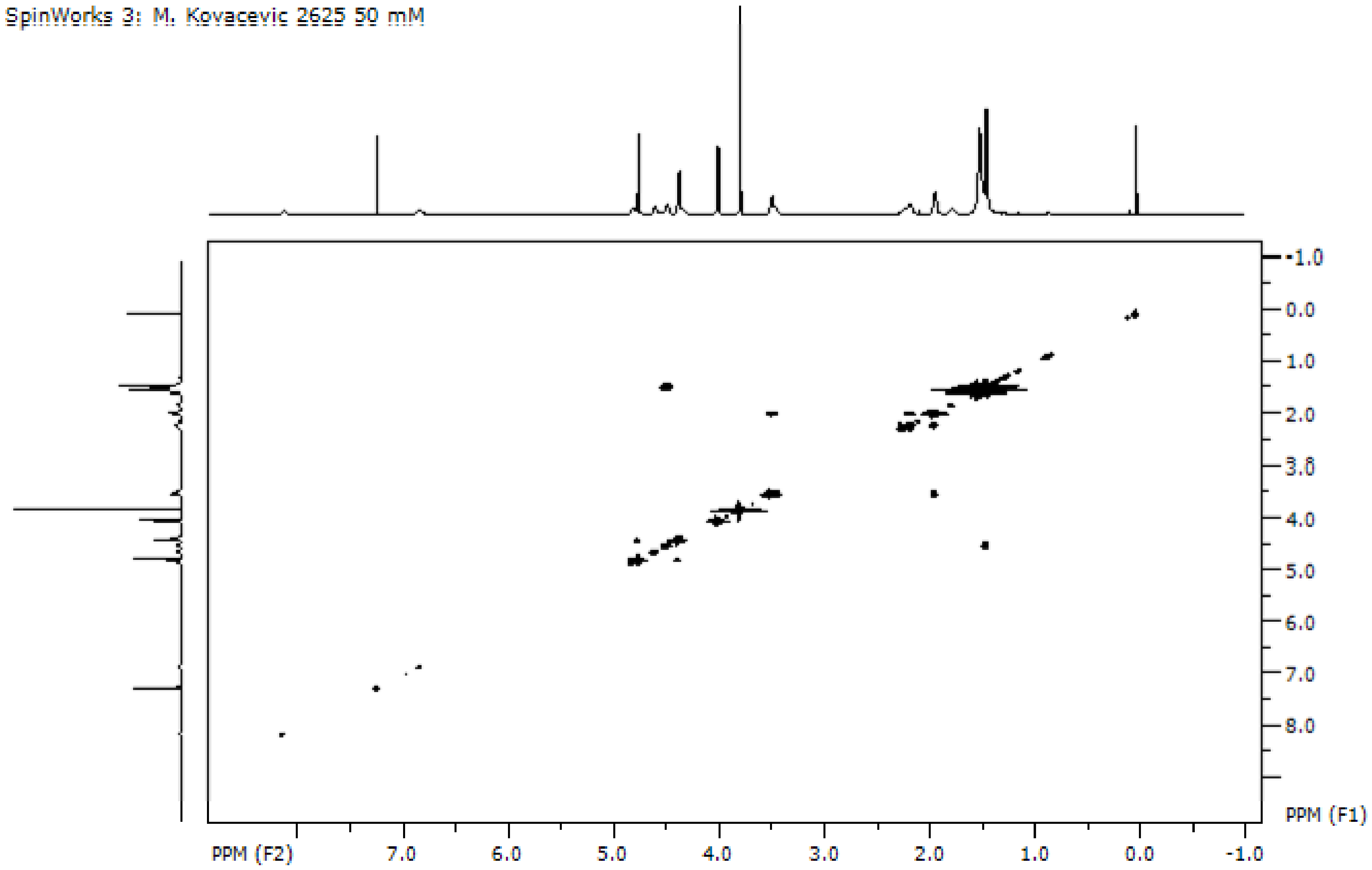

Figure S29. ^{1}H-^{1}H COSY NMR spectrum of compound **4** ($c = 5 \times 10^{-2}$ M).

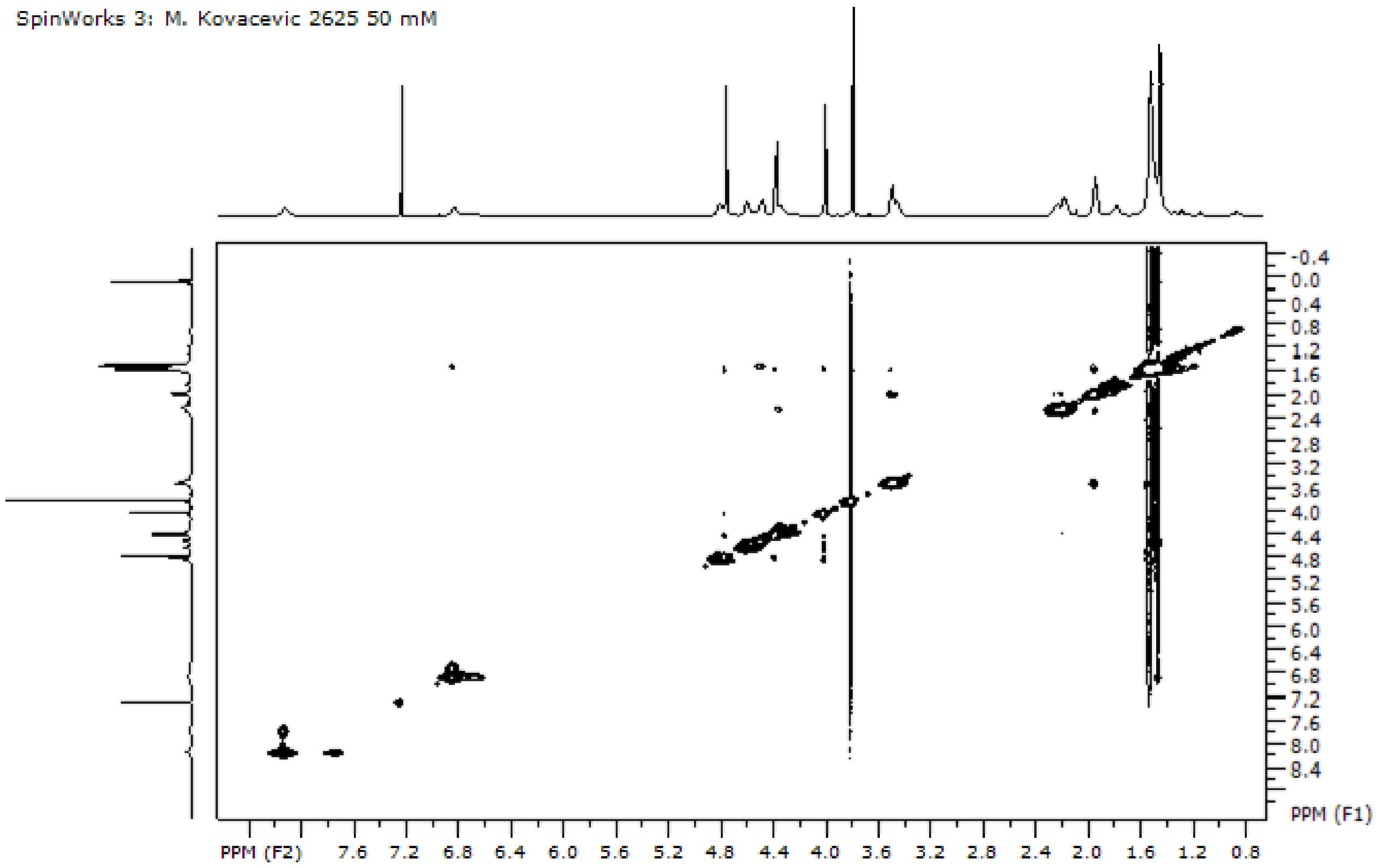

Figure S30. ^{1}H-^{1}H NOESY NMR spectrum of compound **4** ($c = 5 \times 10^{-2}$ M).

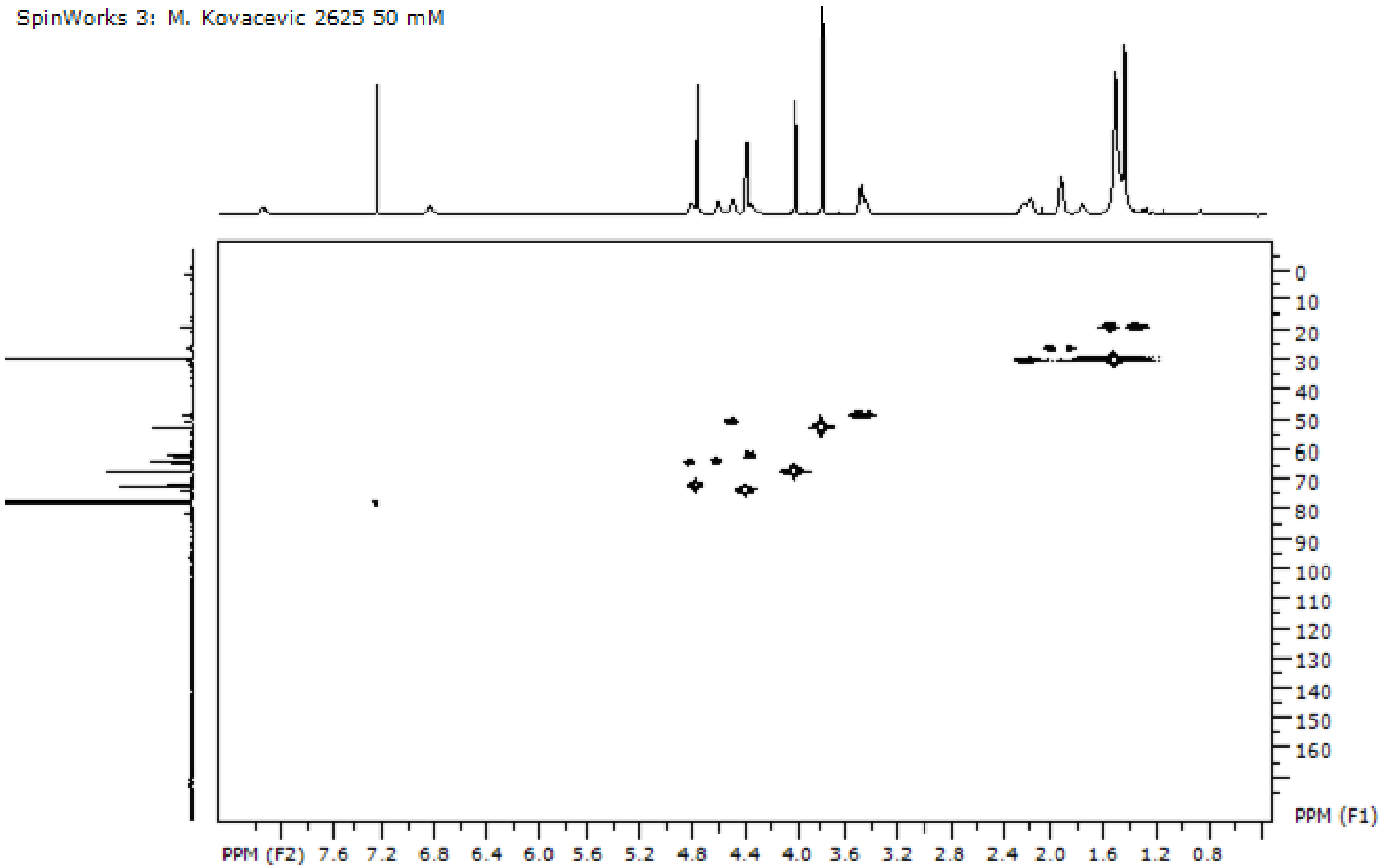

Figure S31. ^{1}H-^{13}C HMQC spectrum of compound 4 ($c = 5 \times 10^{-2}$ M).

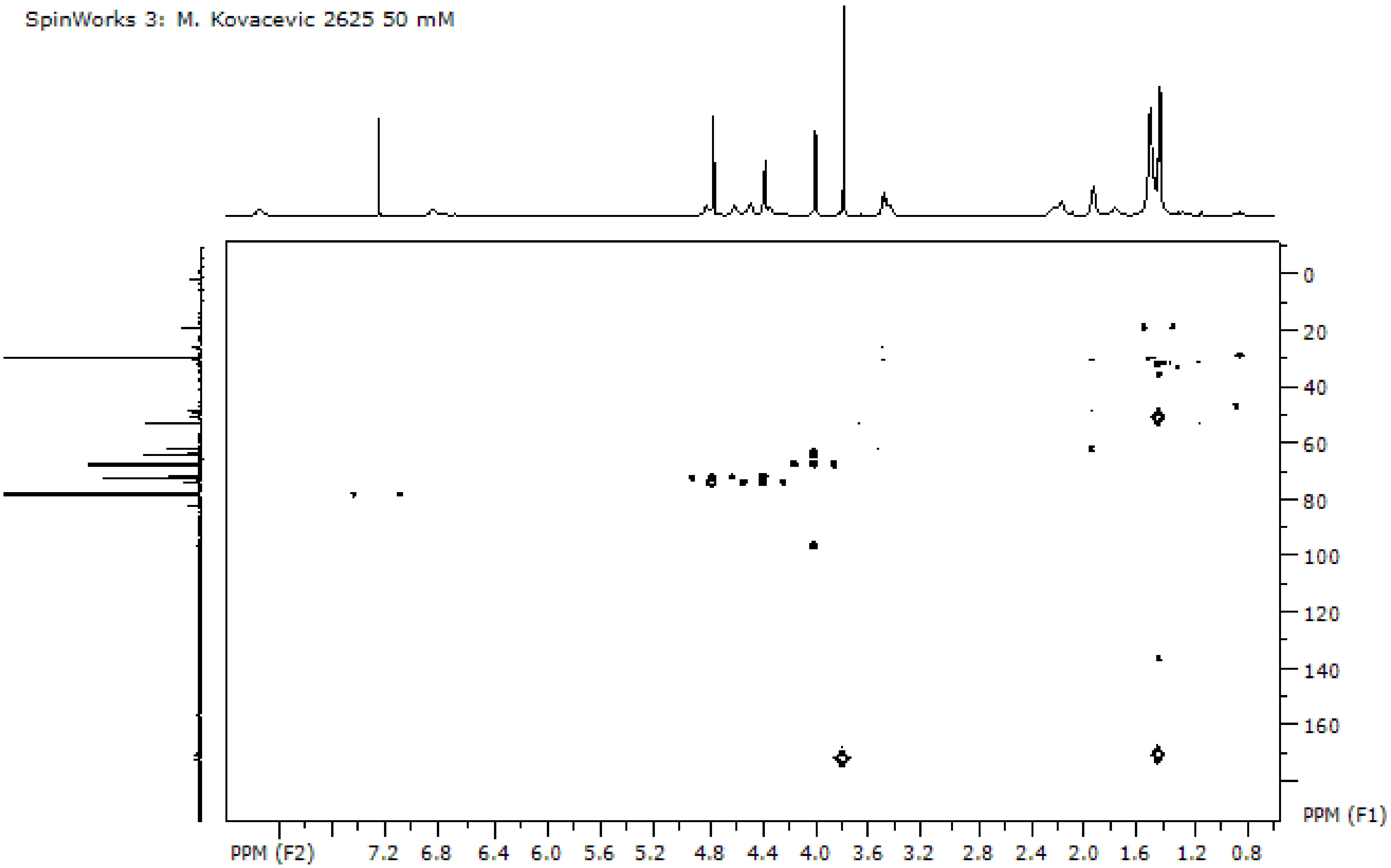

Figure S32. ^{1}H-^{13}C HMBC spectrum of compound 4 ($c = 5 \times 10^{-2}$ M).

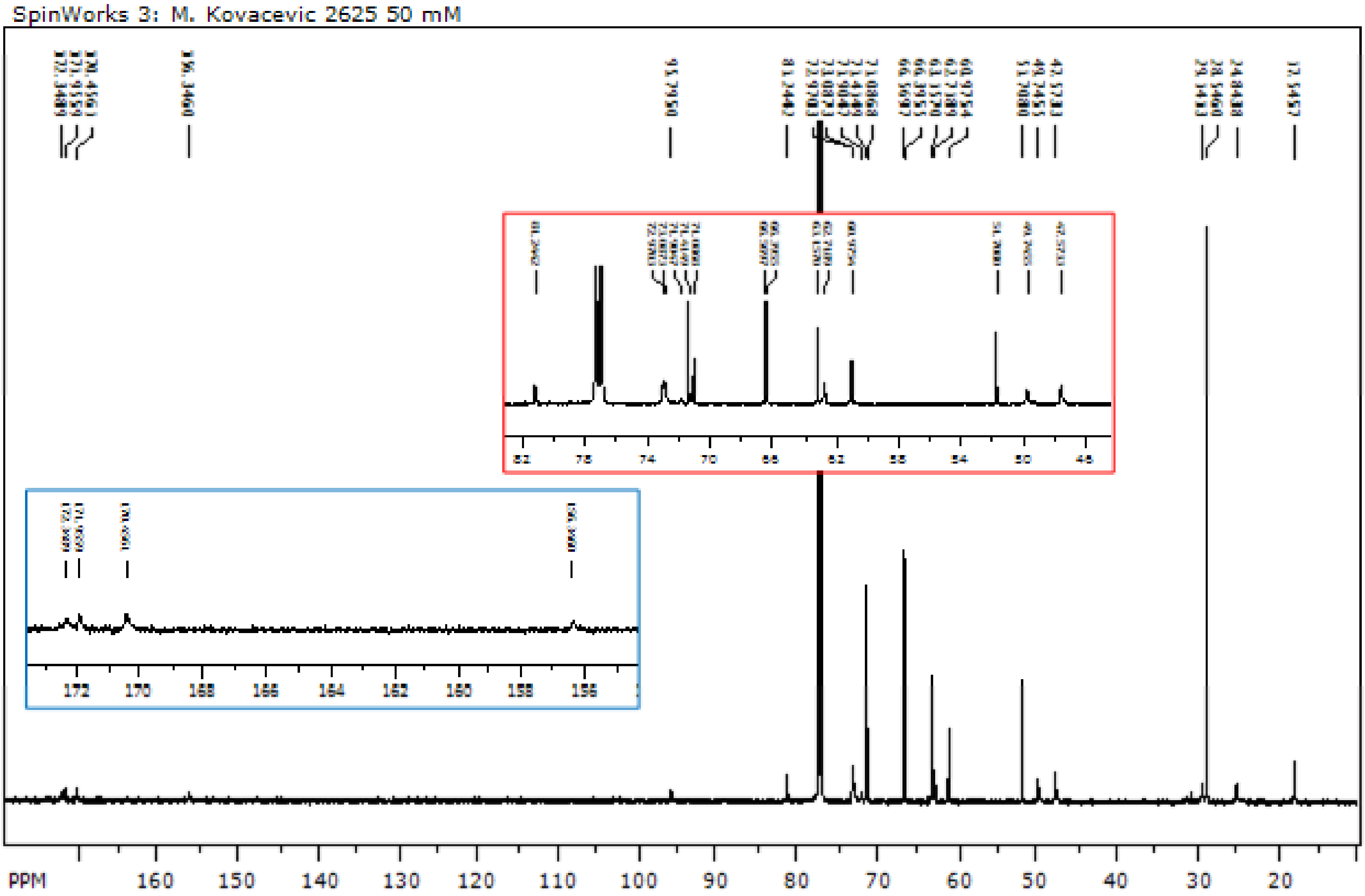

Figure S33. $^{13}C\{^1H\}$ NMR spectrum of compound **4** ($c = 5 \times 10^{-2}$ M).

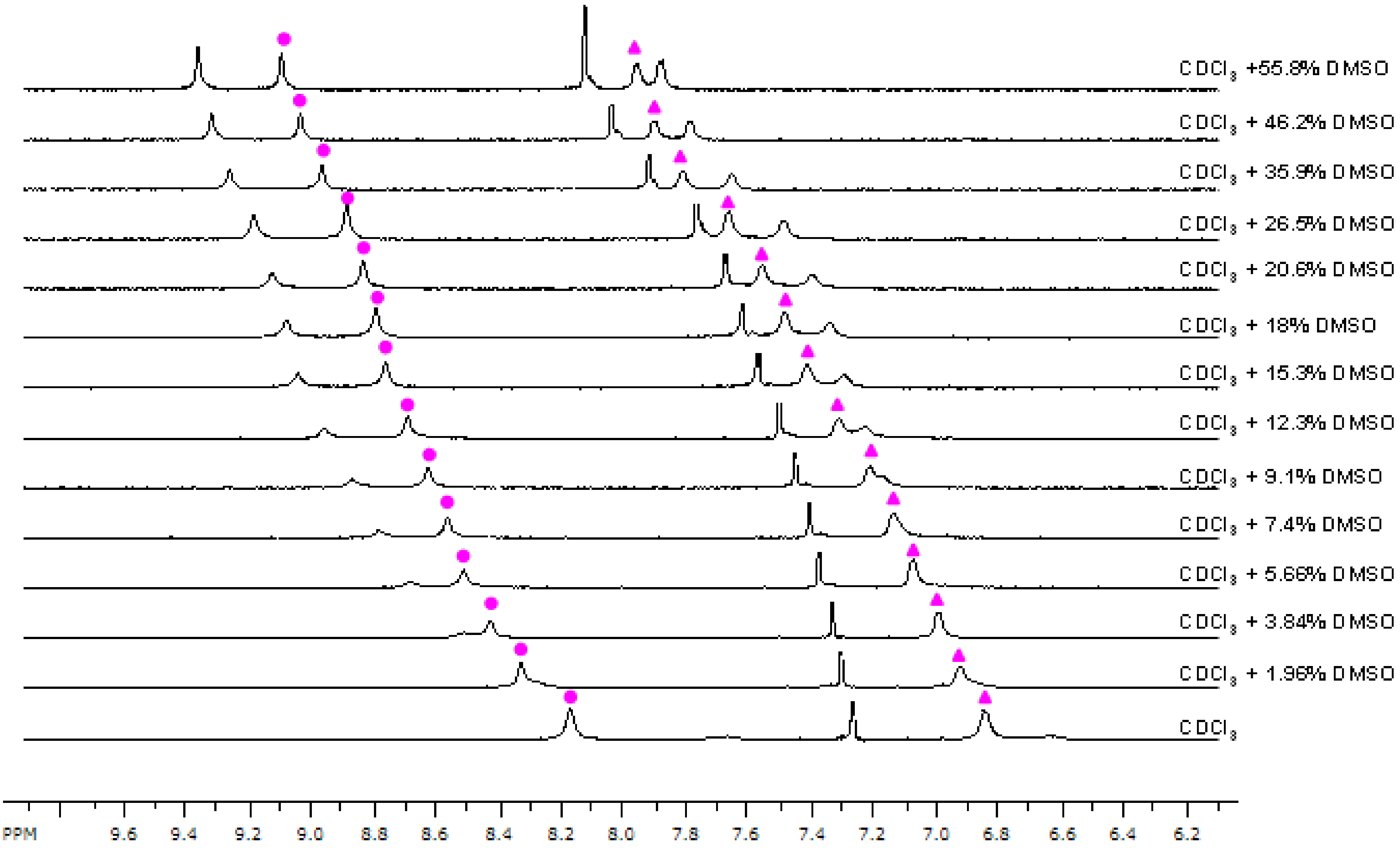

Figure S34. Solvent dependence of NH chemical shifts of compound 4 at varying concentrations of DMSO in $CDCl_3$ ($c = 2.5 \times 10^{-2}$ M).

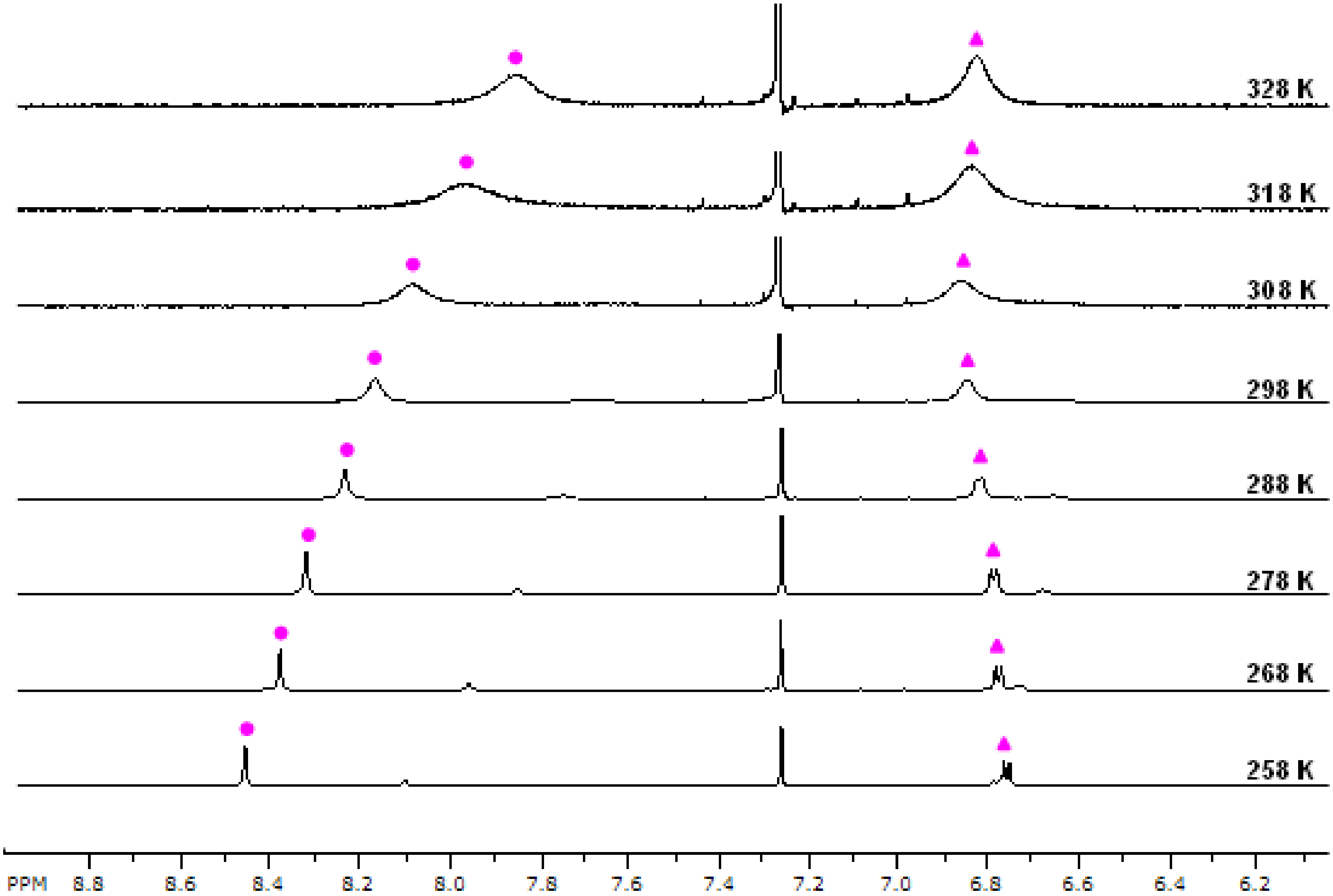

Figure S35. Temperature-dependent NH chemical shifts of compound 4 ($c = 1 \times 10^{-2}$ M).

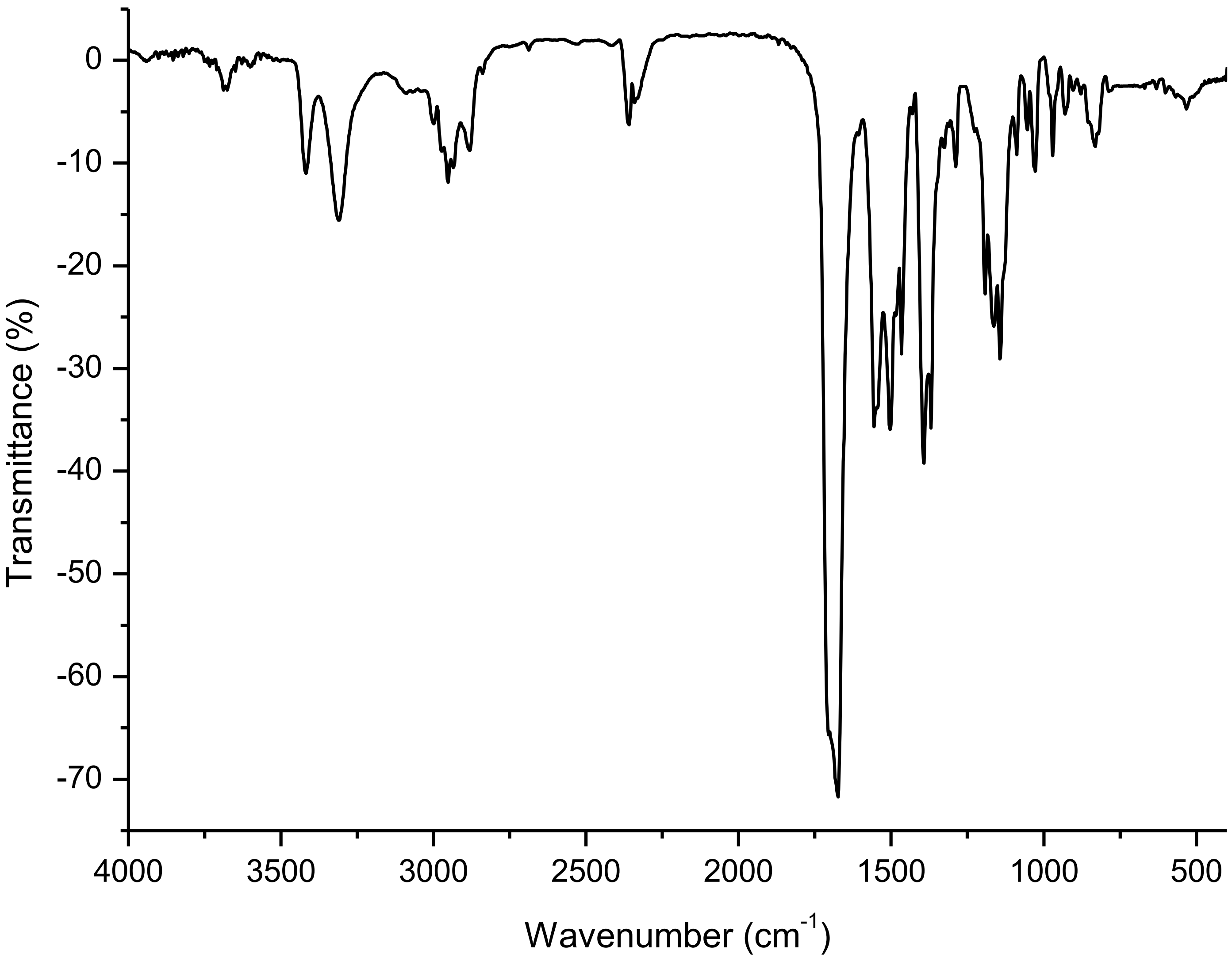

Figure S36. IR spectrum of compound **4** (c = 5 × 10^{-2} M) in DCM.

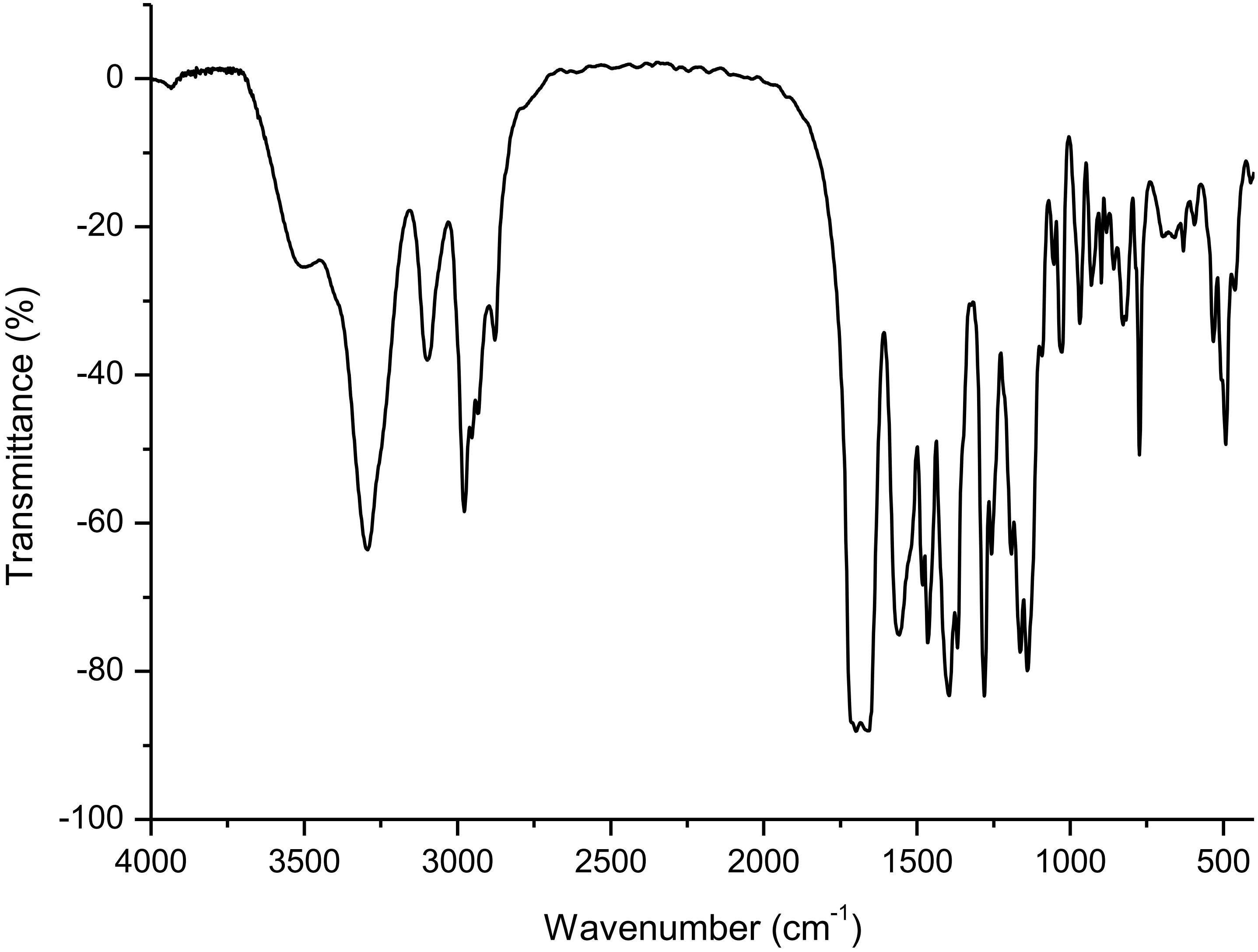

Figure S37. IR spectra of compound **4** (2 mg) in KBr (200 mg).

Ac-L-Pro-L-Ala-NH-Fn-COOMe (5)

Ion type	Calc. mass	Measured mass	Mass error / ppm	Mol. Formula	Int. CAL
M+	469.1300	469.1280	4.3	$C_{22}H_{27}N_3O5Fe$	azitromicin

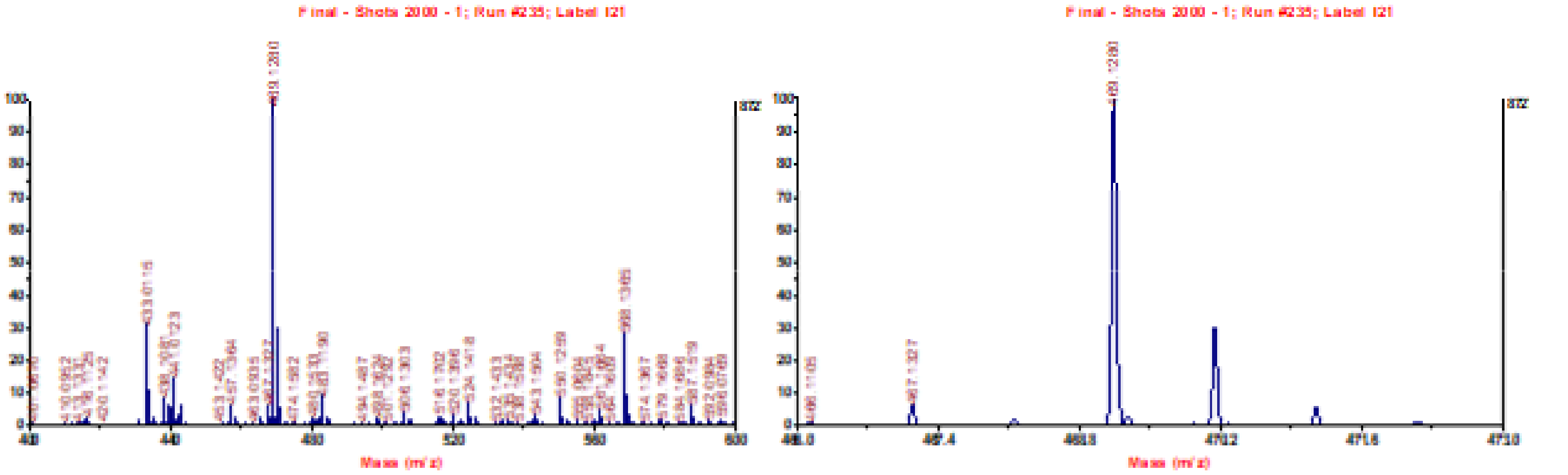

Figure S38. HRMS spectrum of compound 5.

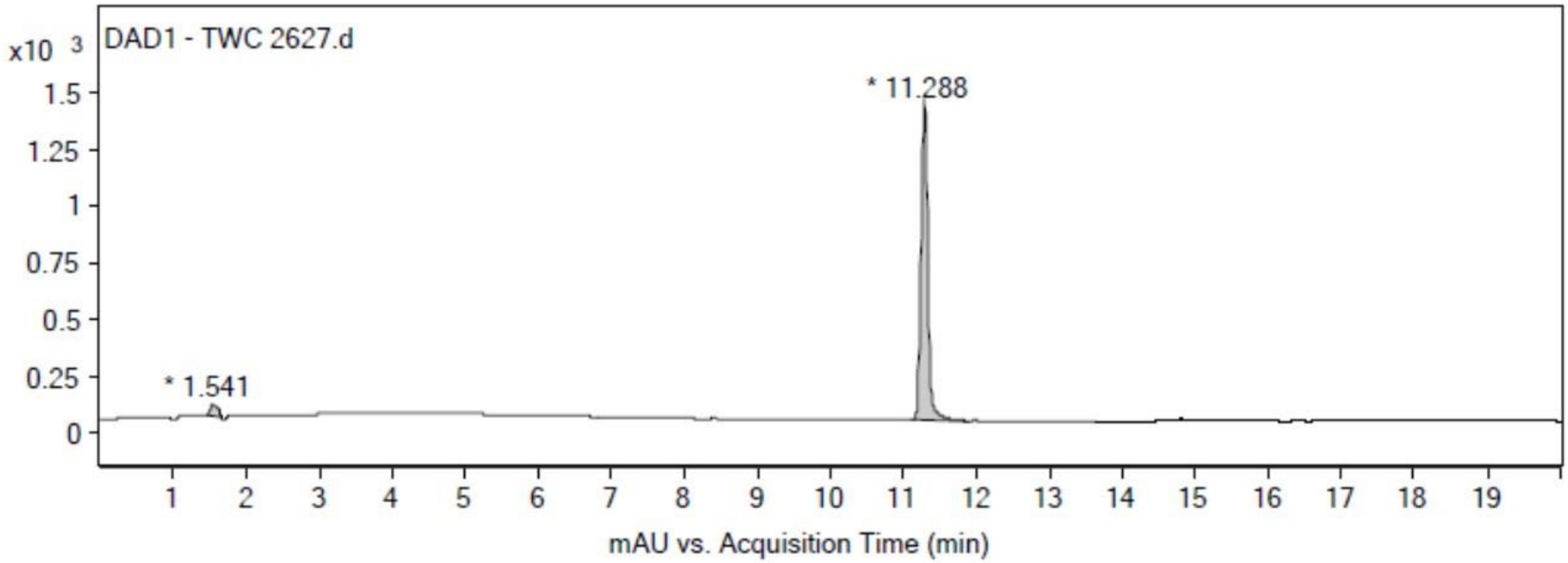

Integration Peak List

Peak	Start	RT	End	Height	Area	Area %
1	1.461	1.541	1.648	46.76	329.46	3.47
2	11.101	11.288	11.881	1411.81	9488.61	100

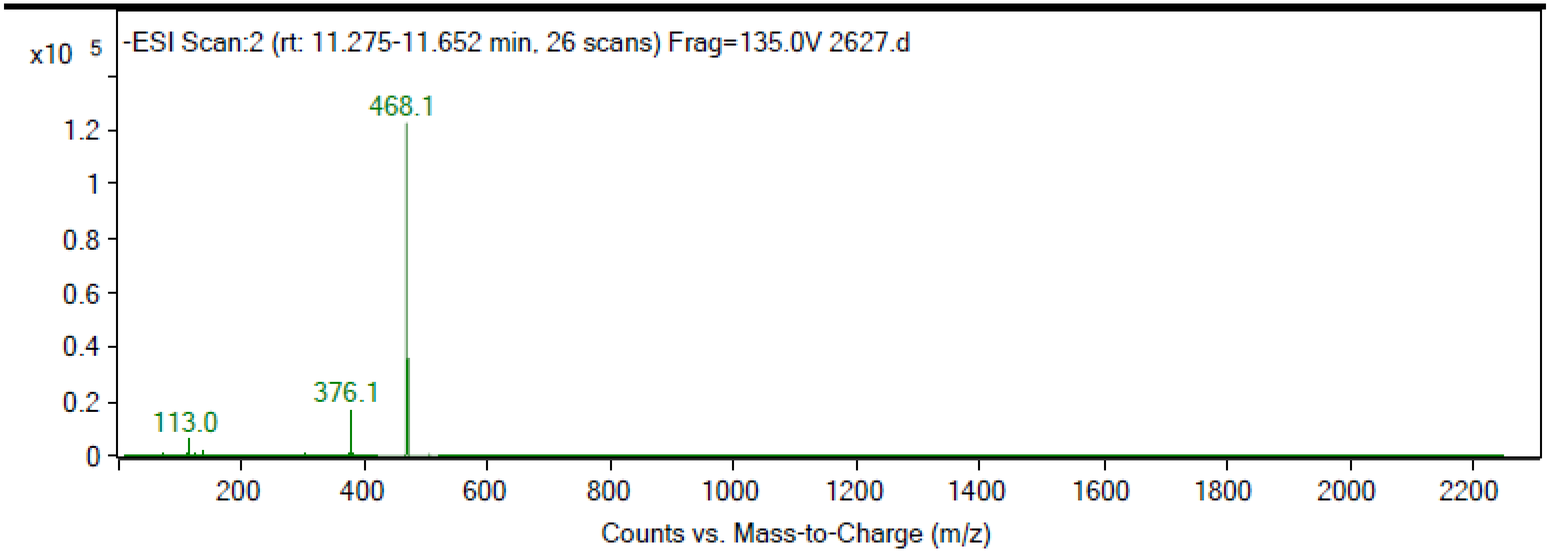

Peak List

m/z	z	Abund
113		6106.96
137		1766.12
376.1	1	16761.93
377	1	2996.78
466.1		8795.53
468.1	1	122427.03
469.1	1	35895.96
470.1	1	6283.23

Figure S39. HPLC-ESI spectra of compound **5.**

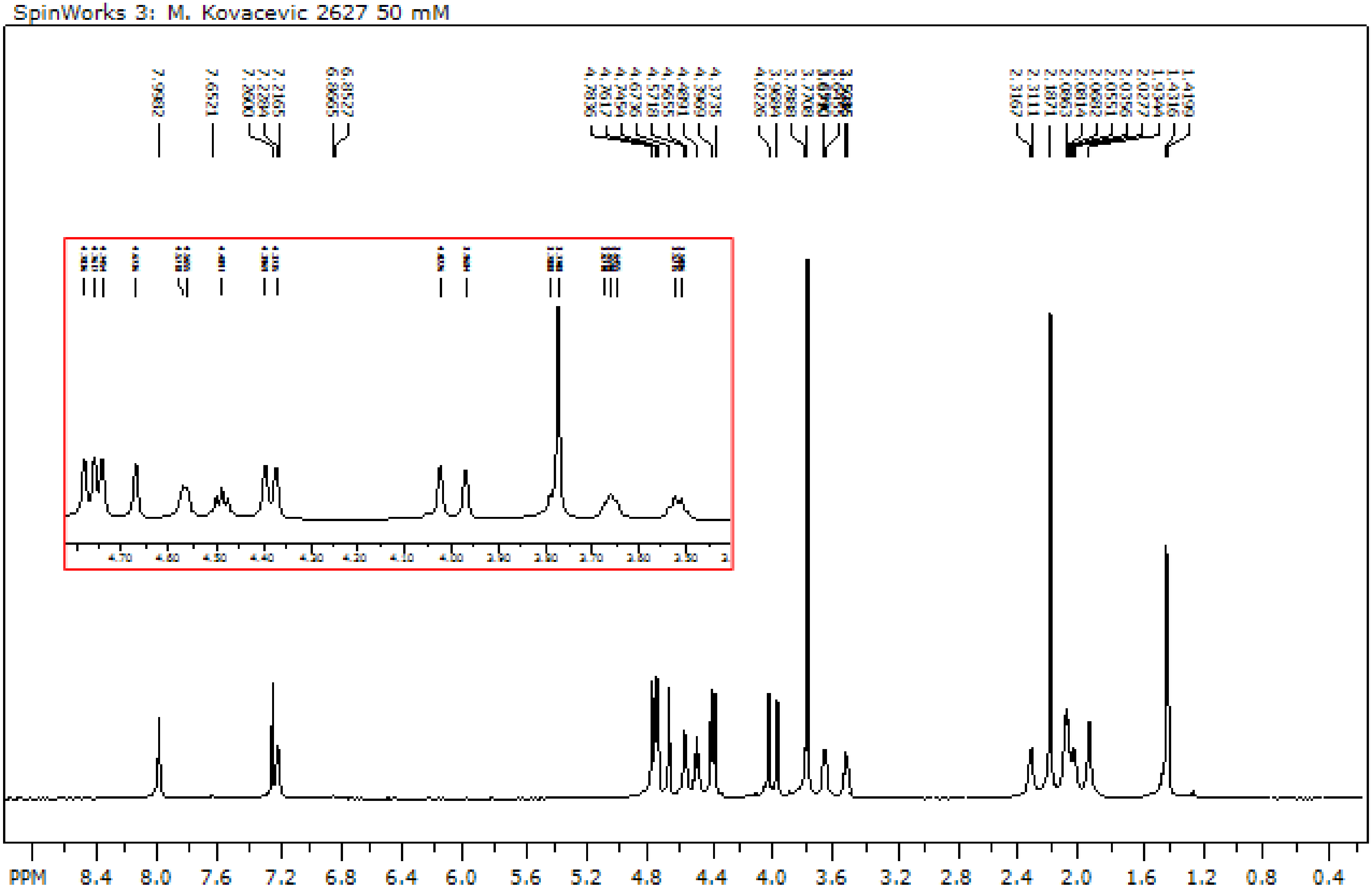

Figure S40. ¹H NMR spectrum of compound 5 ($c = 5 \times 10^{-2}$ M).

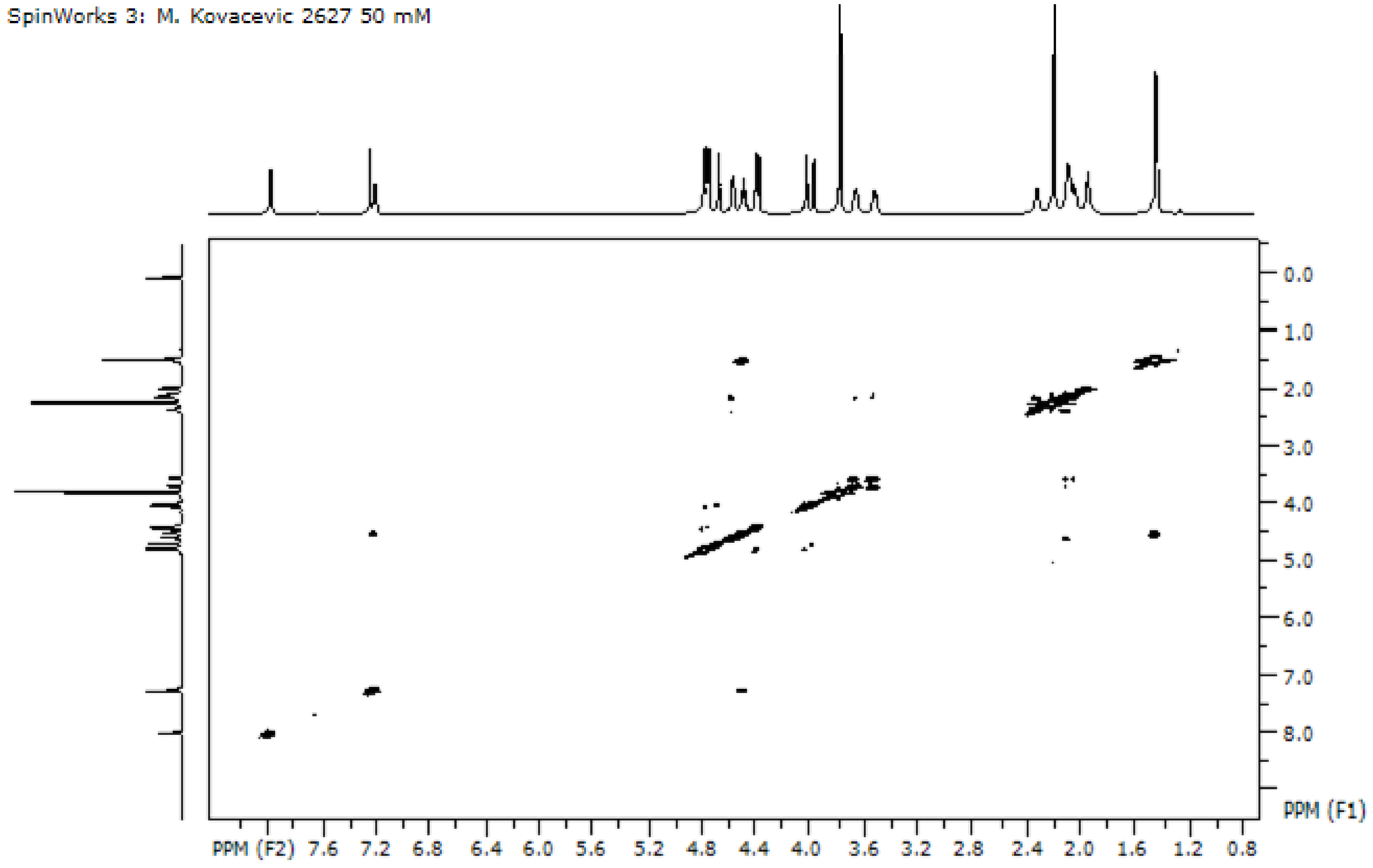

Figure S41. ¹H-¹H COSY NMR spectrum of compound 5 ($c = 5 \times 10^{-2}$ M).

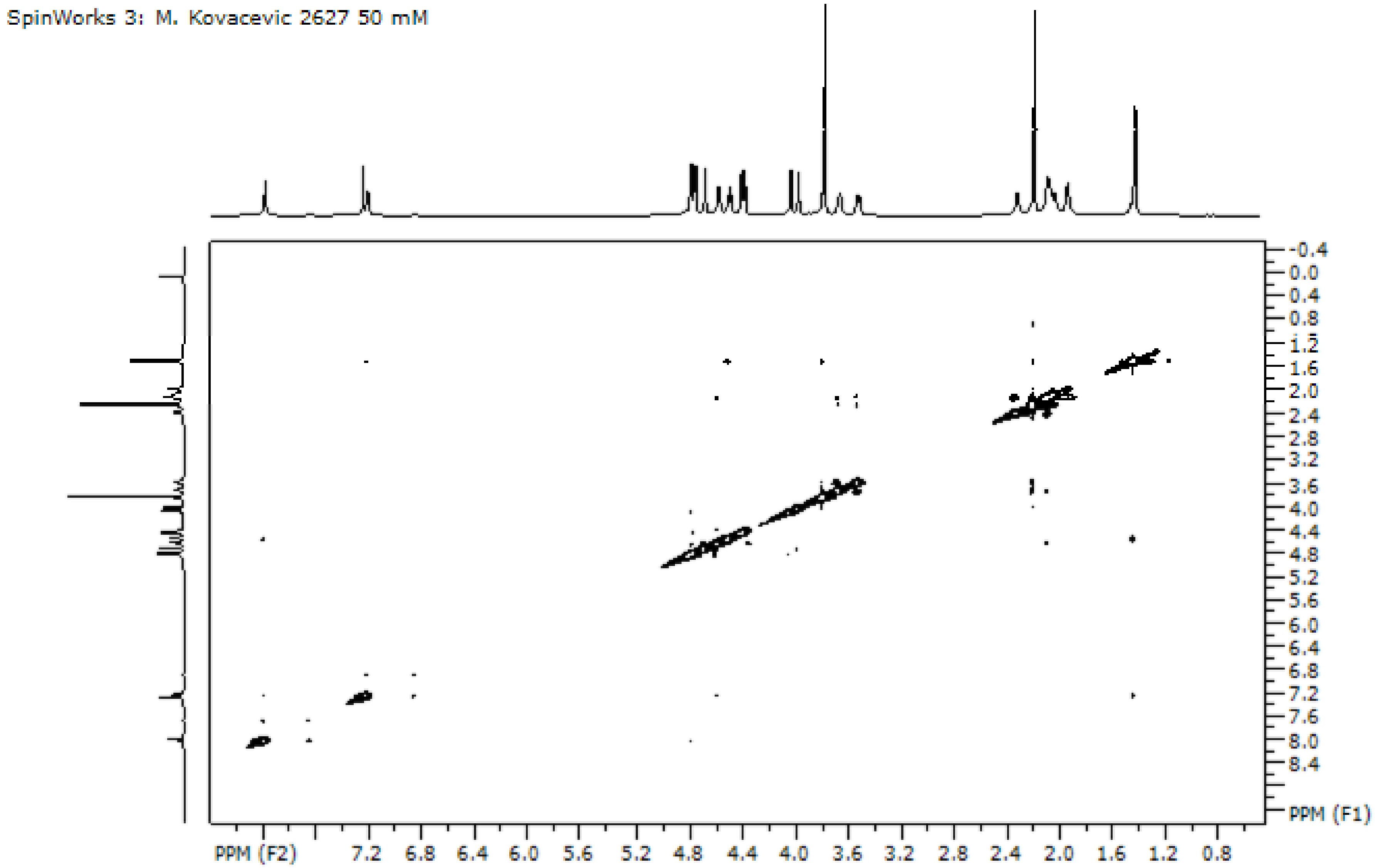

Figure S42. ^{1}H-^{1}H NOESY NMR spectrum of compound 5 ($c = 5 \times 10^{-2}$ M).

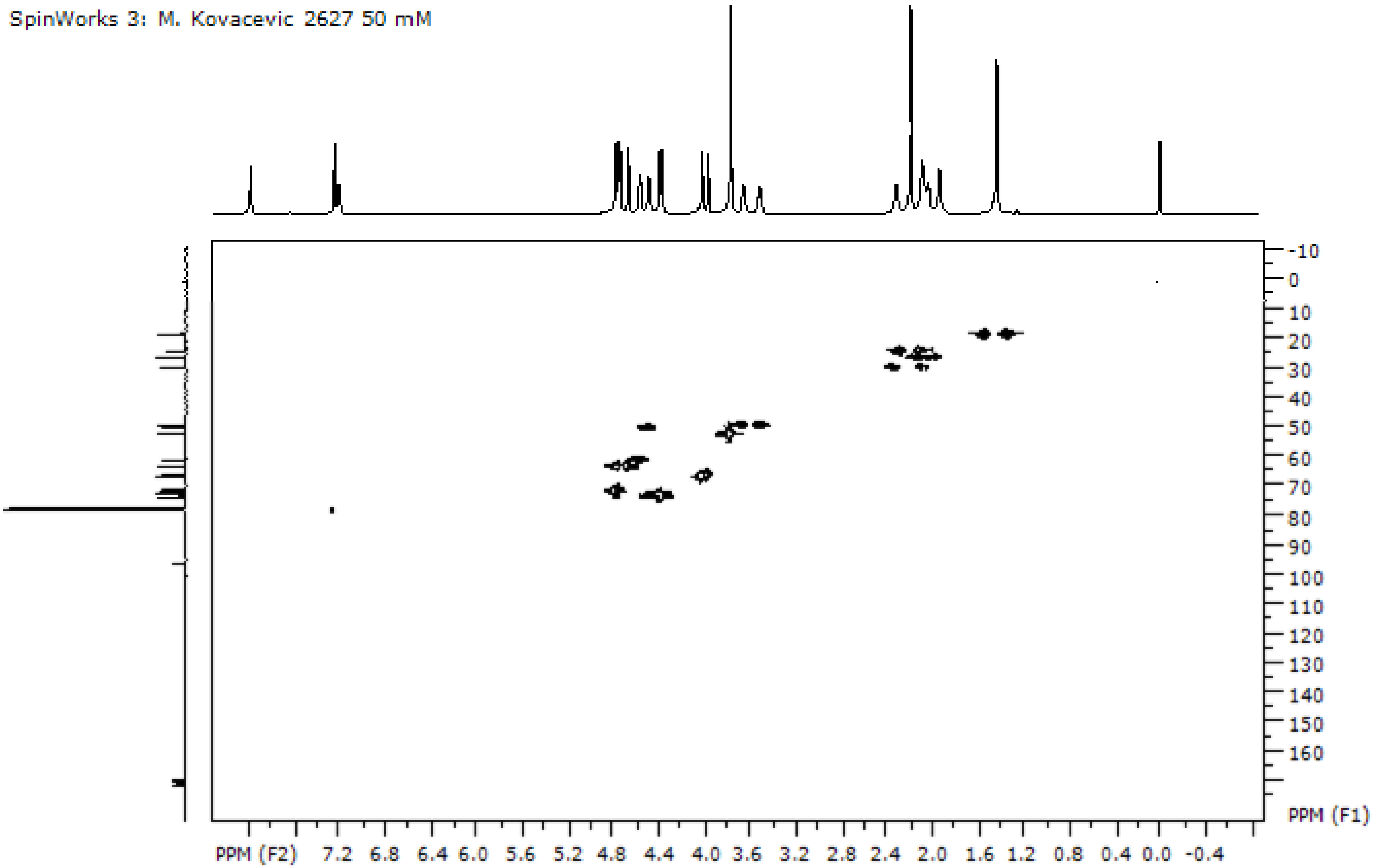

Figure S43. ^{1}H-^{13}C HMQC spectrum of compound 5 ($c = 5 \times 10^{-2}$ M).

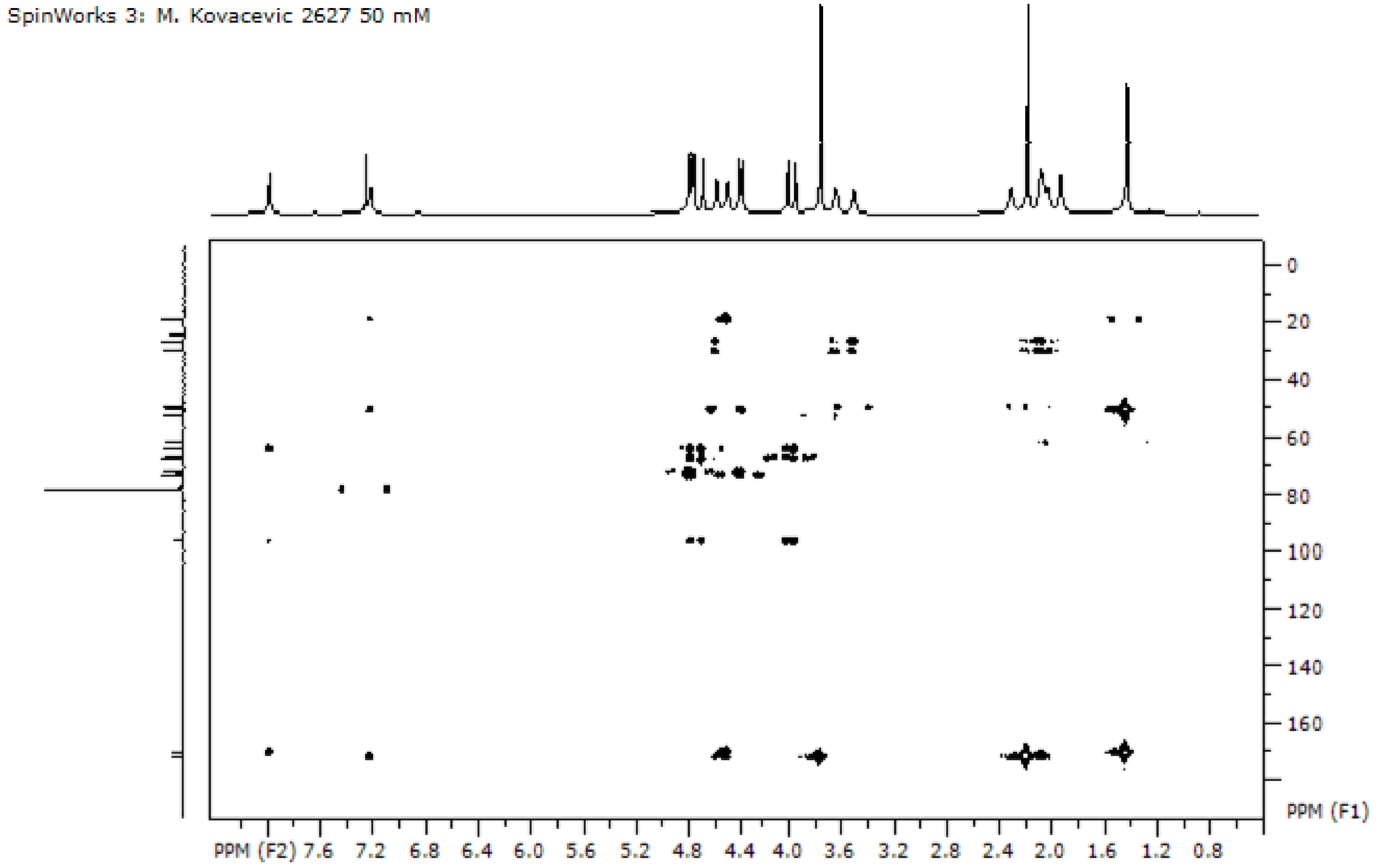

Figure S44. ^{1}H-^{13}C HMBC spectrum of compound 5 ($c = 5 \times 10^{-2}$ M).

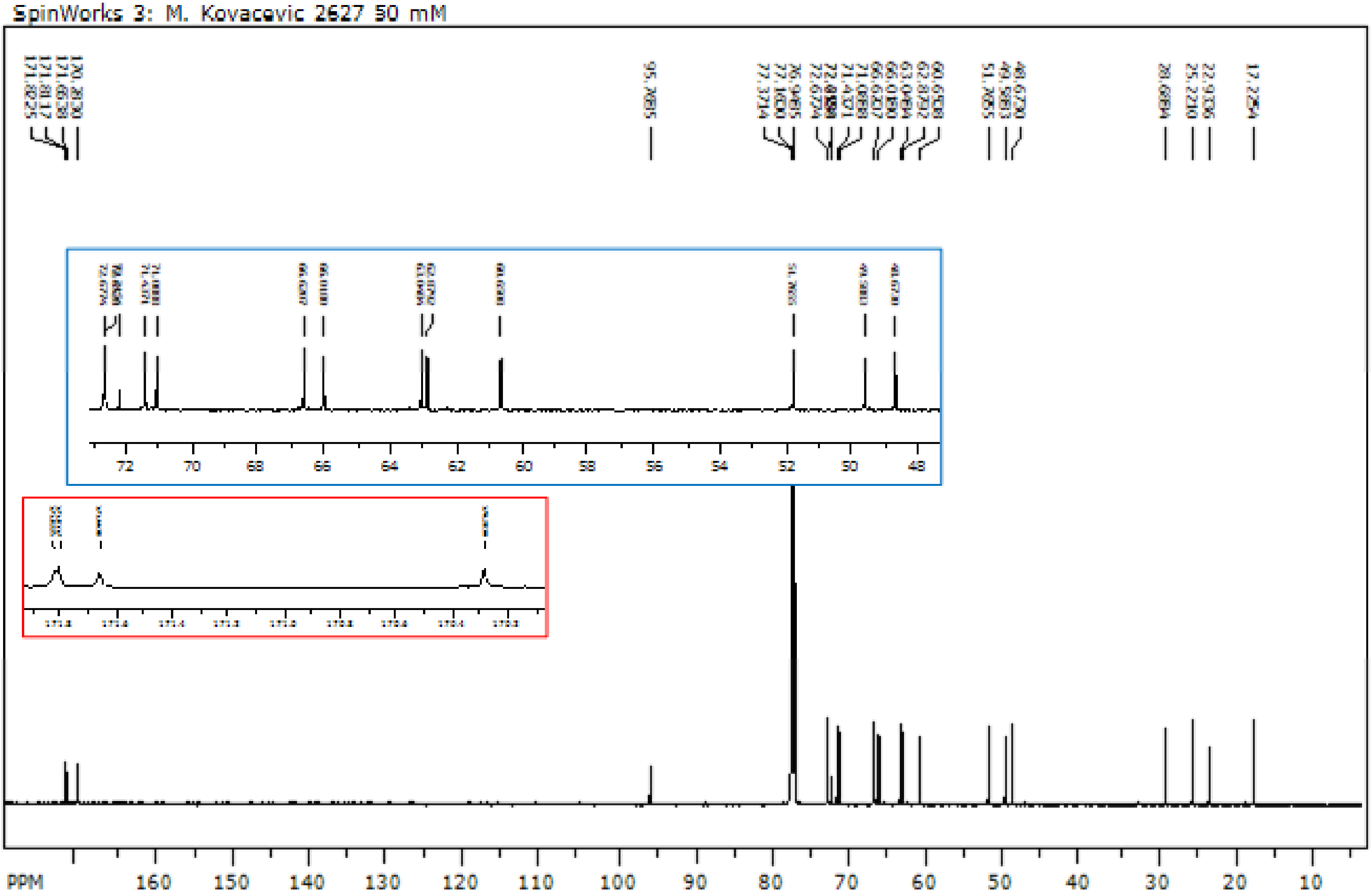

Figure S45. $^{13}C\{^{1}H\}$ NMR spectrum of compound 5 ($c = 5 \times 10^{-2}$ M).

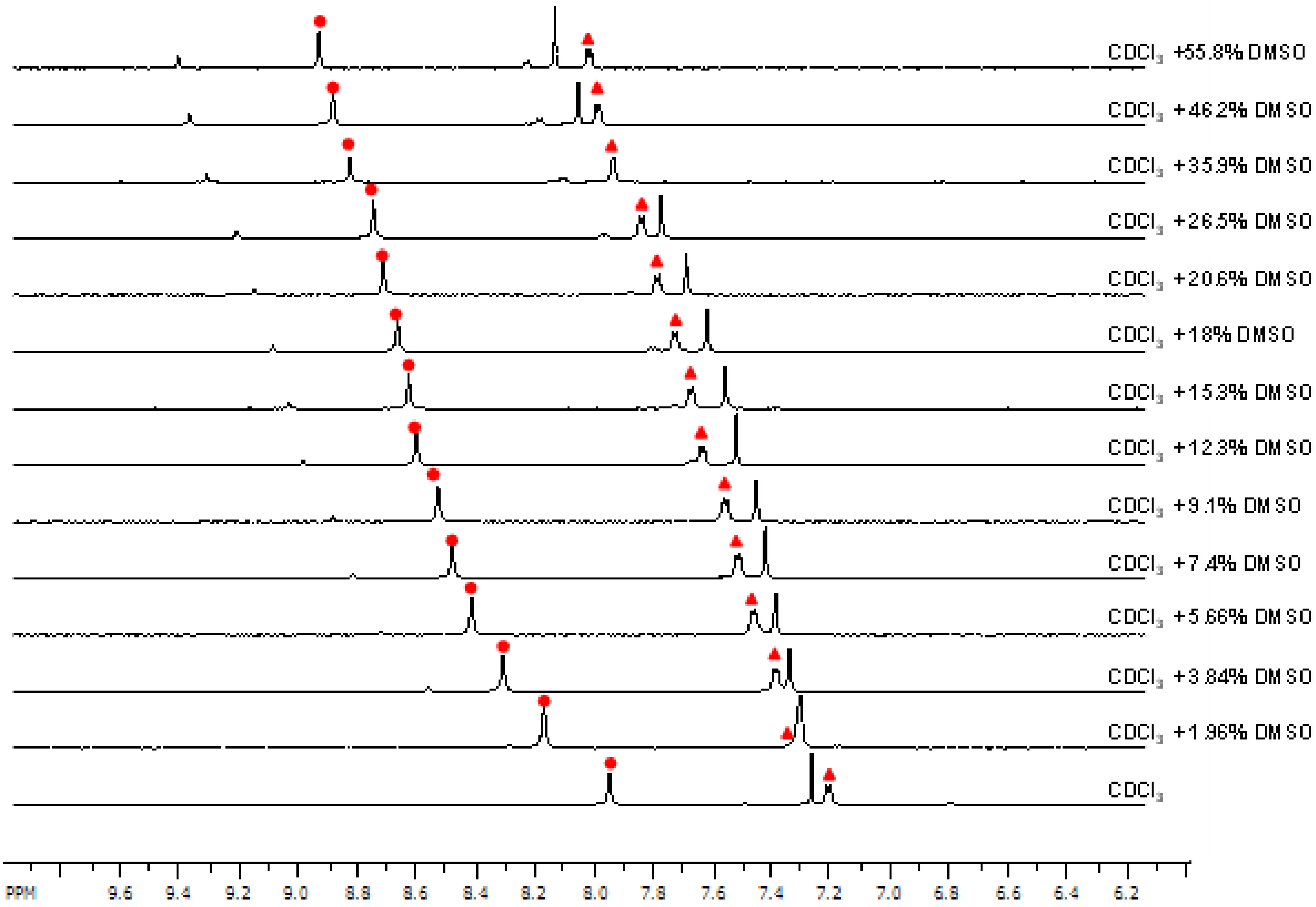

Figure S46. Solvent dependence of NH chemical shifts of compound 5 at varying concentrations of DMSO in $CDCl_3$ ($c = 2.5 \times 10^{-3}$ M).

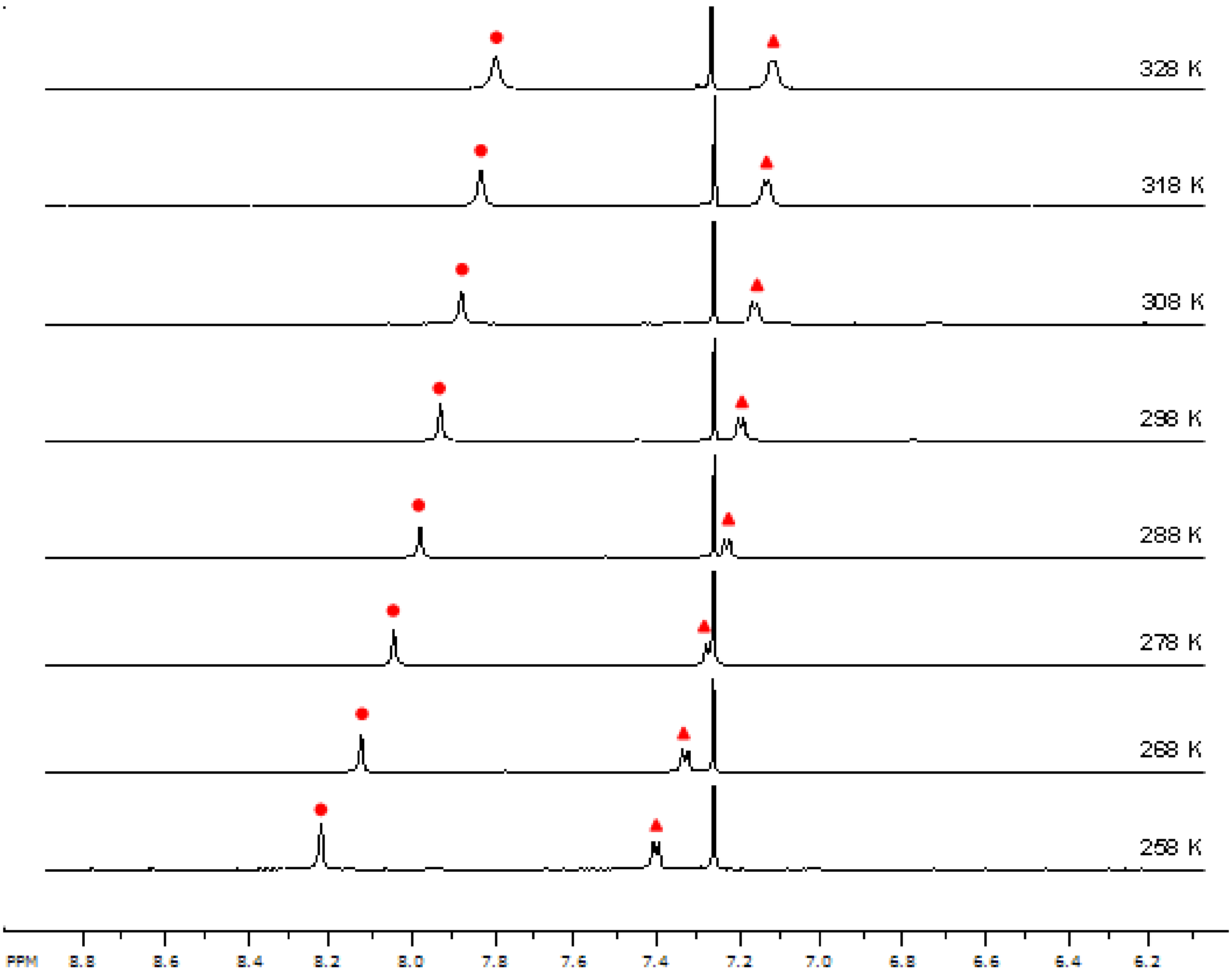

Figure S47. Temperature-dependent NH chemical shifts of compound 5 ($c = 1 \times 10^{-2}$ M).

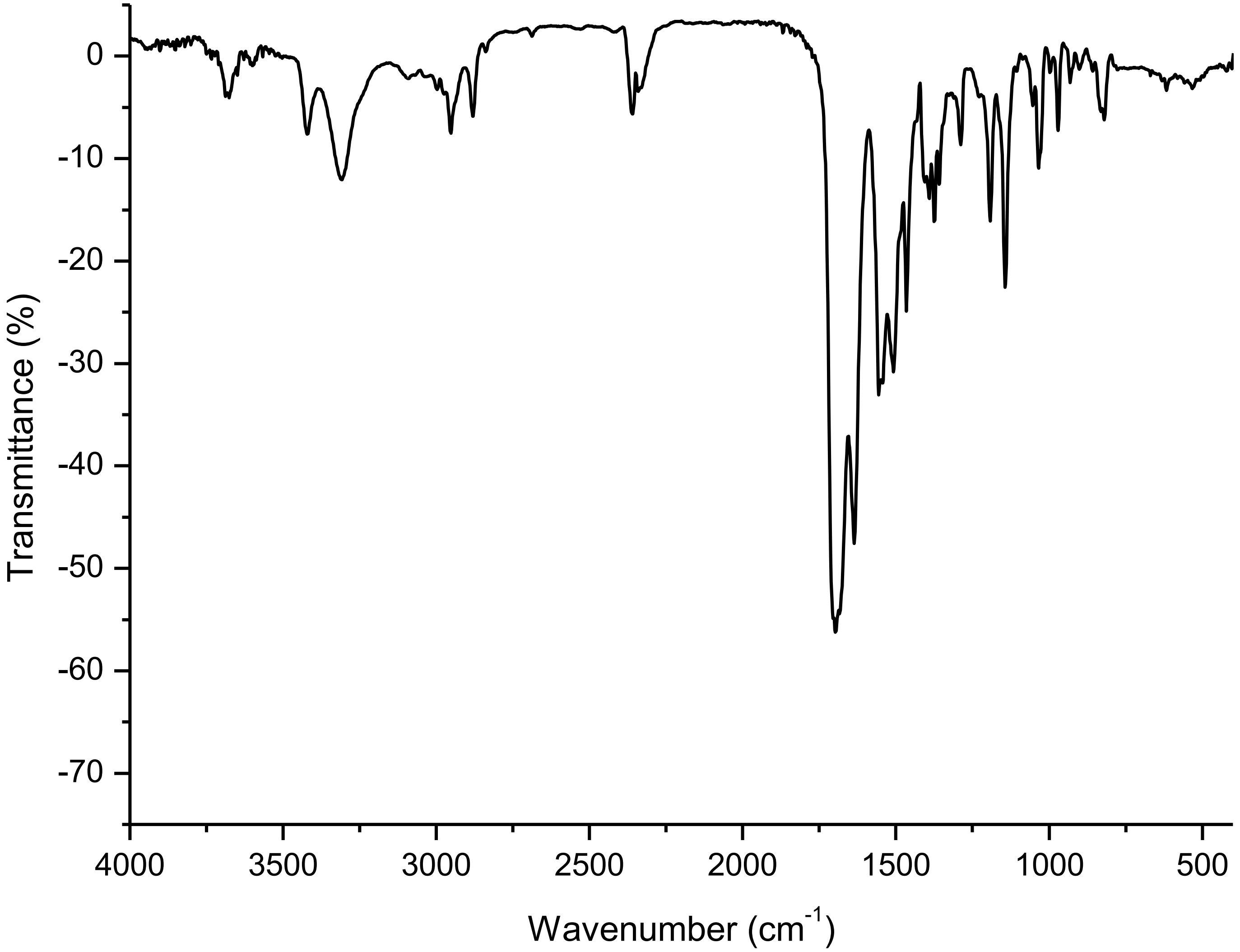

Figure S48. IR spectrum of compound **5** (c = 5 × 10^{-2} M) in DCM.

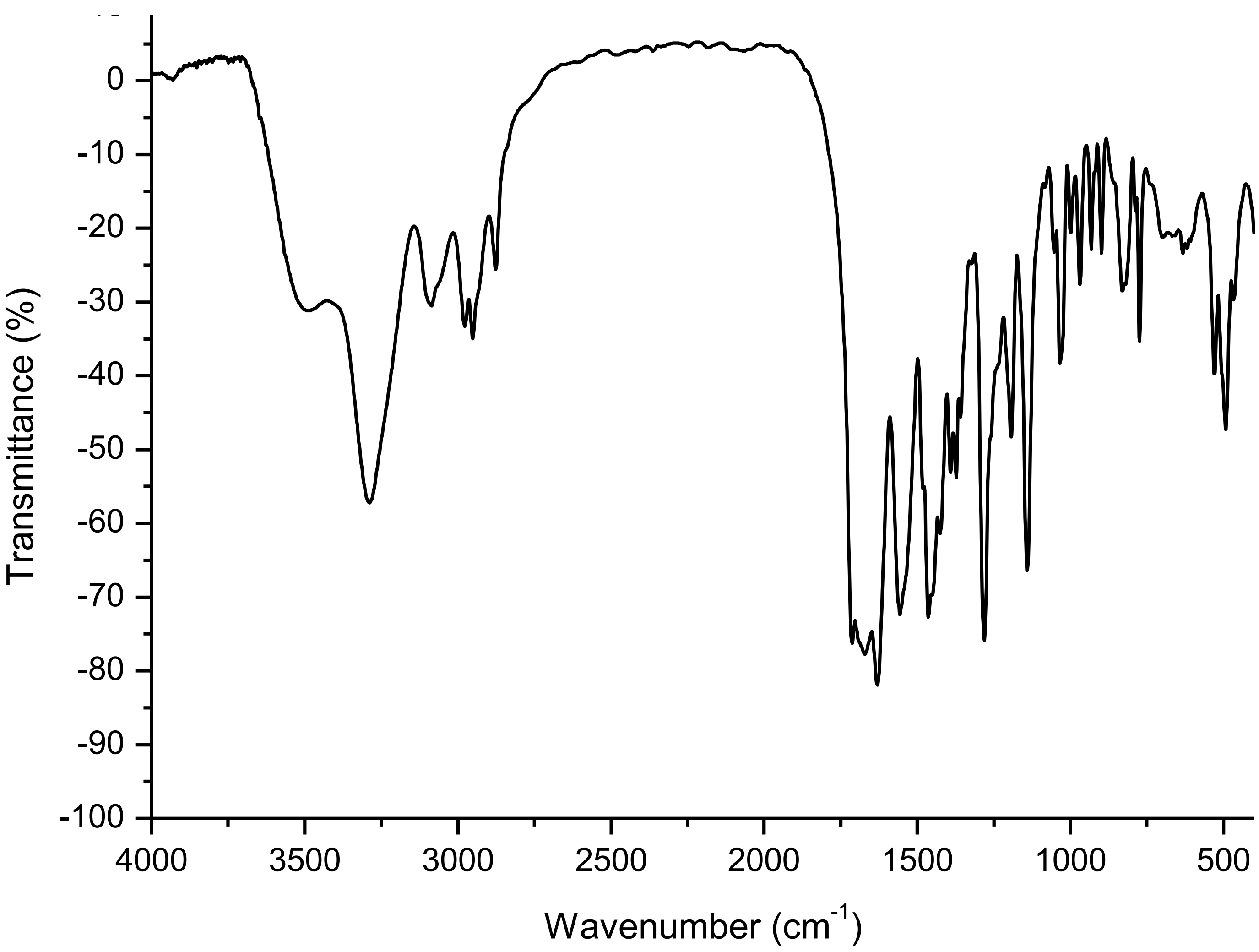

Figure S49. IR spectrum of compound **5** (2 mg) in KBr (200 mg).

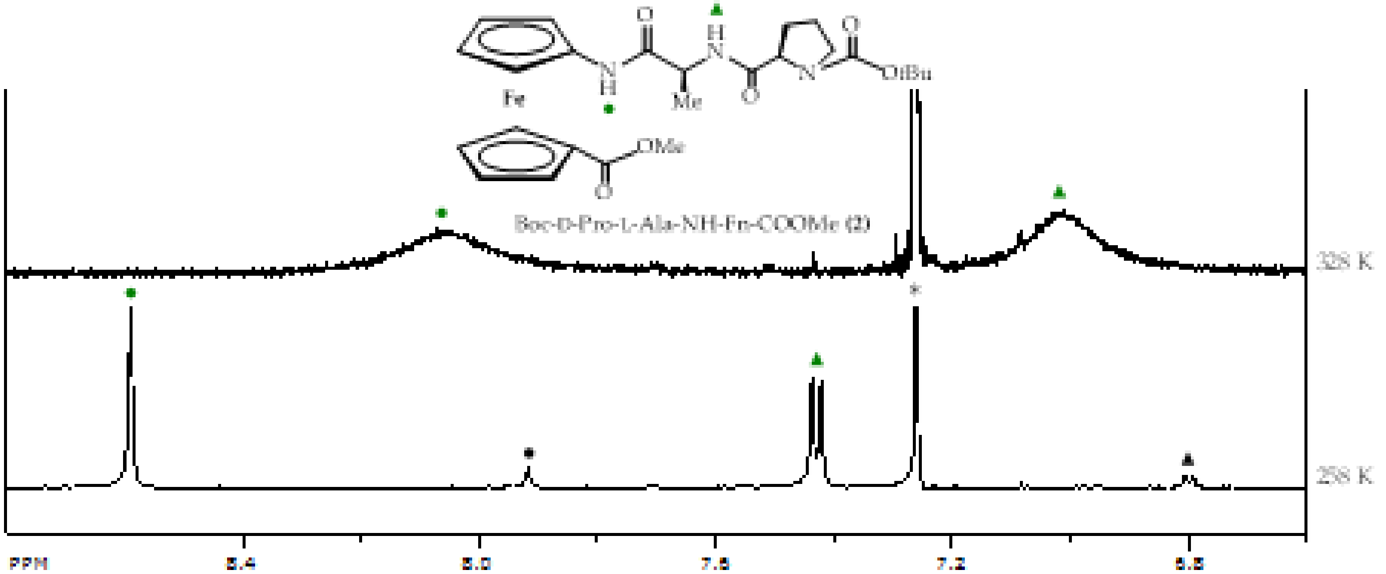
Fe
OMe
OtBu
Me
Boc-D-Pro-L-Ala-NH-Fn-COOMe (2)
328 K
258 K
PPM
8.4
8.0
7.6
7.2
6.8

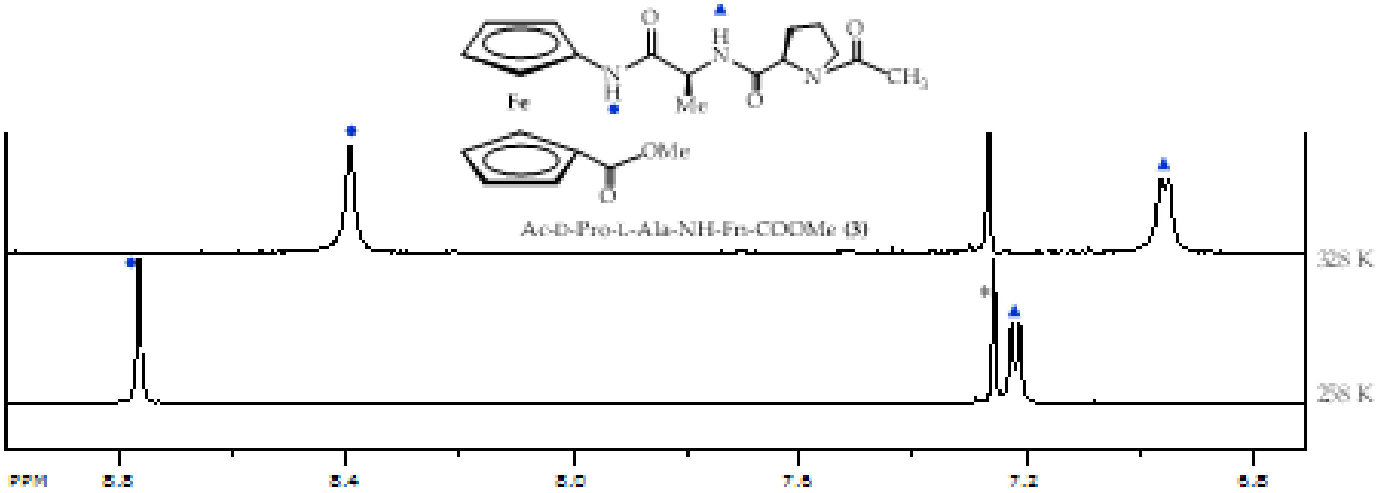
Fe
OMe
Me
Ac-D-Pro-L-Ala-NH-Fn-COOMe (3)
328 K
258 K
PPM
8.8
8.4
8.0
7.6
7.2
6.8

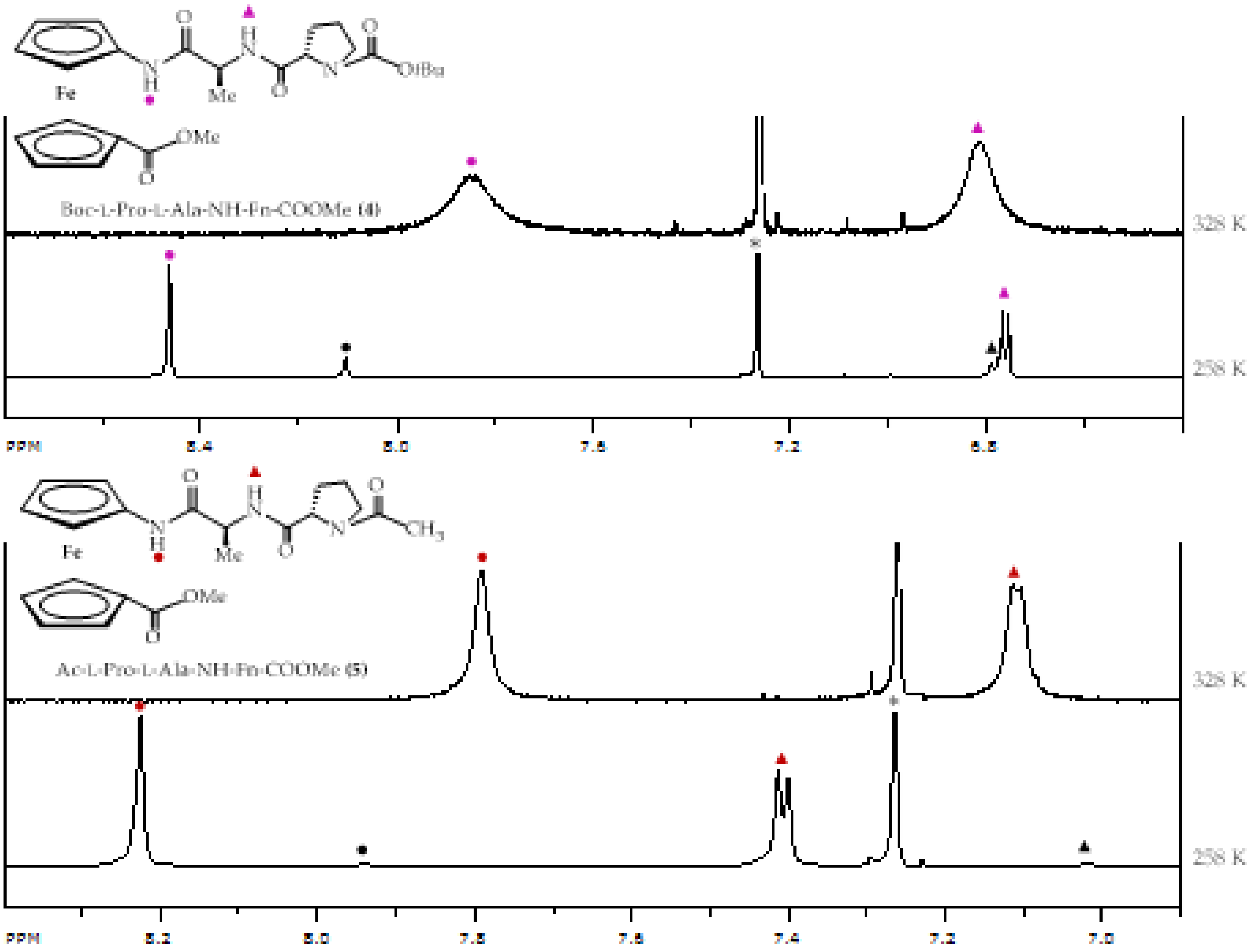

Figure S50. The influence of increased temperature on the *cis*/*trans* signals coalescence in peptides **2-5.**

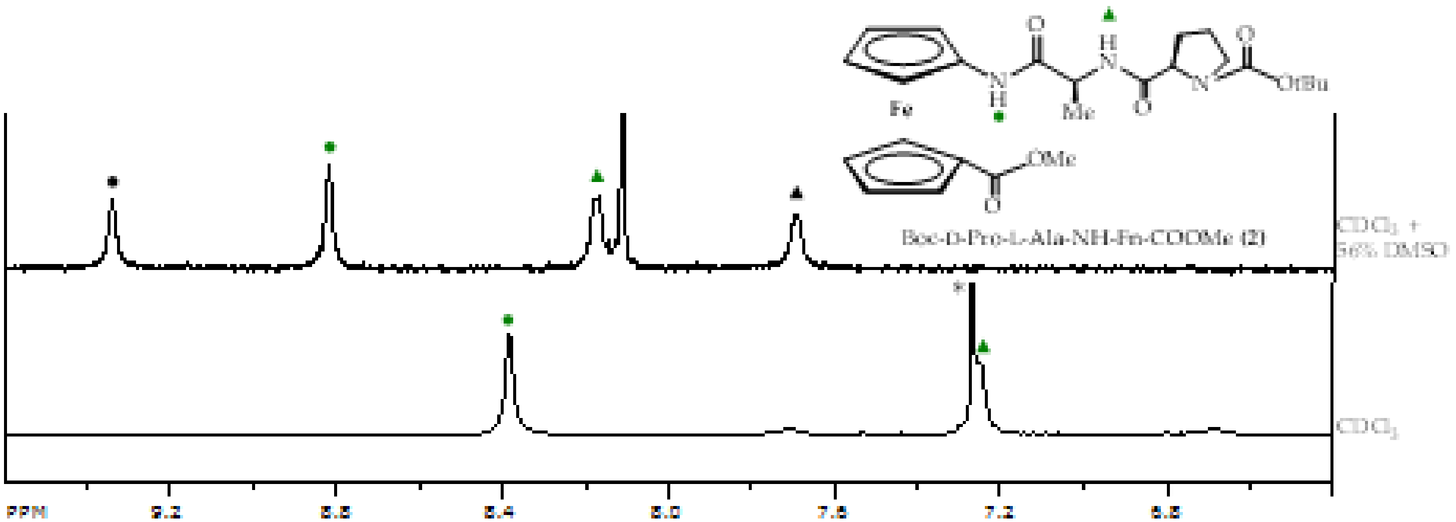
Fe
OMe
OtBu
Me
Boc-D-Pro-L-Ala-NH-Fn-COOMe (2)
CDCl$_3$ +
56% DMSO
CDCl$_3$
PPM
9.2
8.8
8.4
8.0
7.6
7.2
6.8

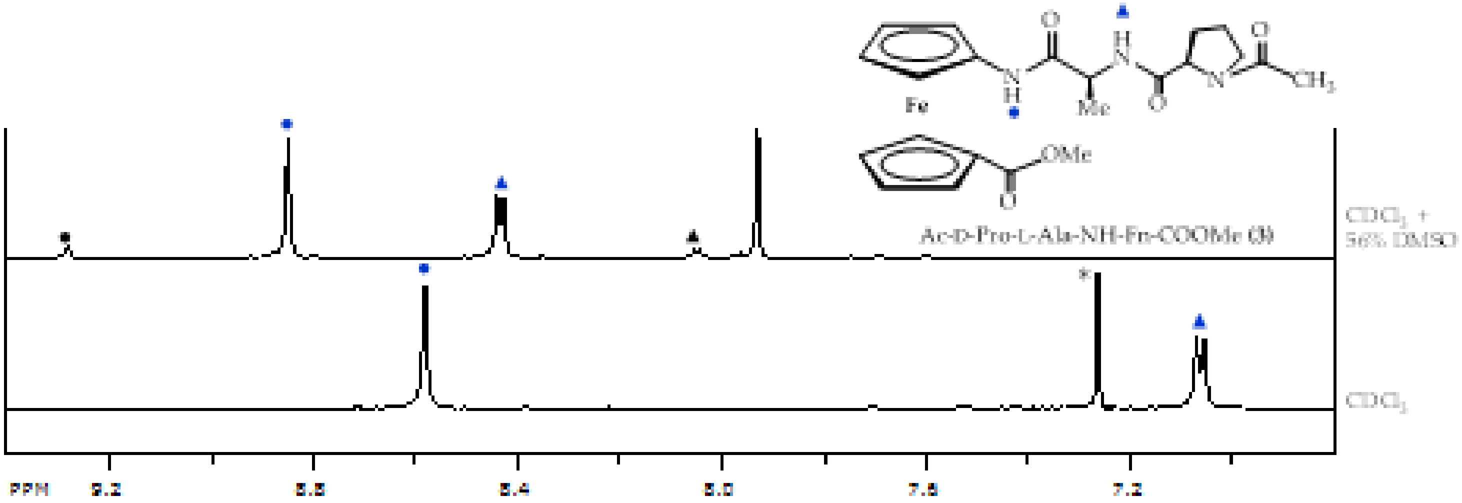
Fe
OMe
CH$_3$
Me
Ac-D-Pro-L-Ala-NH-Fn-COOMe (3)
CDCl$_3$ +
56% DMSO
CDCl$_3$
PPM
9.2
8.8
8.4
8.0
7.6
7.2

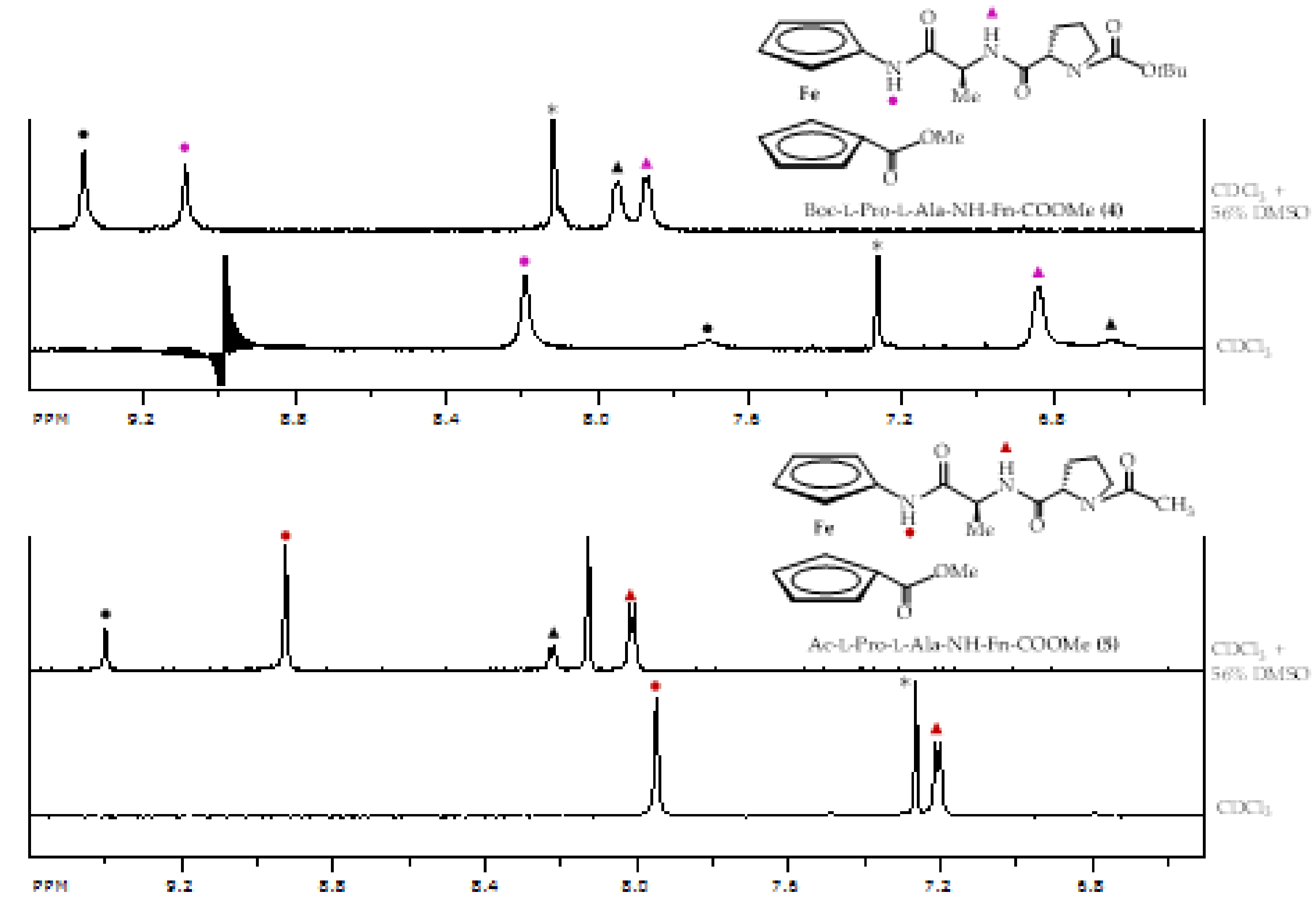

Figure S51. The influence of DMSO on *cis*-*trans* isomerization of a proline imide bond in peptides 2-5.

X-ray crystal structure analysis

Table S52. Crystallographic, data collection and refinement data.

Compound	**2**	**5**
Empirical formula	$C_{25}H_{33}FeN_3O_6$	$C_{22}H_{26}FeN_3O_6$
Formula wt. / g mol^{-1}	527.39	484.31
Colour	yellow	yellow
Crystal dimensions / mm	0.20 x 0.09 x 0.05	0.15 x 0.09 x 0.04
Space group	$P\ 2_12_12_1$	$C\ 2$
a / Å	5.88390(10)	22.3927(8)
b / Å	17.6280(2)	6.2677(3)
c / Å	24.6876(4)	16.5434(7)
α / °	90	90
β / °	90	104.007(4)
γ / °	90	90
Z	4	4
V / Å^3	2560.63(7)	2252.84(17)
D_{calc} / g cm^{-3}	1.368	1.475
λ / Å	1.54179 (Cu$K\alpha$)	1.54179 (Cu$K\alpha$)
μ / mm^{-1}	5.094	5.742

Compound	**2**	**5**
Θ range / °	3.58 – 79.66	2.75 – 76.27
T / K	293(2)	293(1)
Diffractometer type	Synergy S	Xcalibur Nova
Range of h, k, l	$-4 < h < 7$; $-20 < k < 22$; $-31 < l < 26$	$-28 < h < 25$; $-5 < k < 7$; $-20 < l < 15$
Reflections collected	18409	5290
Independent reflections	5486	3415
Observed reflections ($I \geq 2\sigma$)	5251	2765
Absorption correction	Multi-scan	Multi-scan
T_{min}, T_{max}	0.1844; 1.0000	0.2363; 1.0000
R_{int}	0.0314	0.0862
R (F)	0.0293	0.0702
R_w (F^2)	0.0791	0.1998
Goodness of fit	1.071	1.019
H atom treatment	Constrained	Constrained
No. of parameters	316	280
No. of restraints	0	41
$\Delta\rho_{max}$, $\Delta\rho_{min}$ (eÅ^{-3})	0.367; -0.371	0.535; -0.623

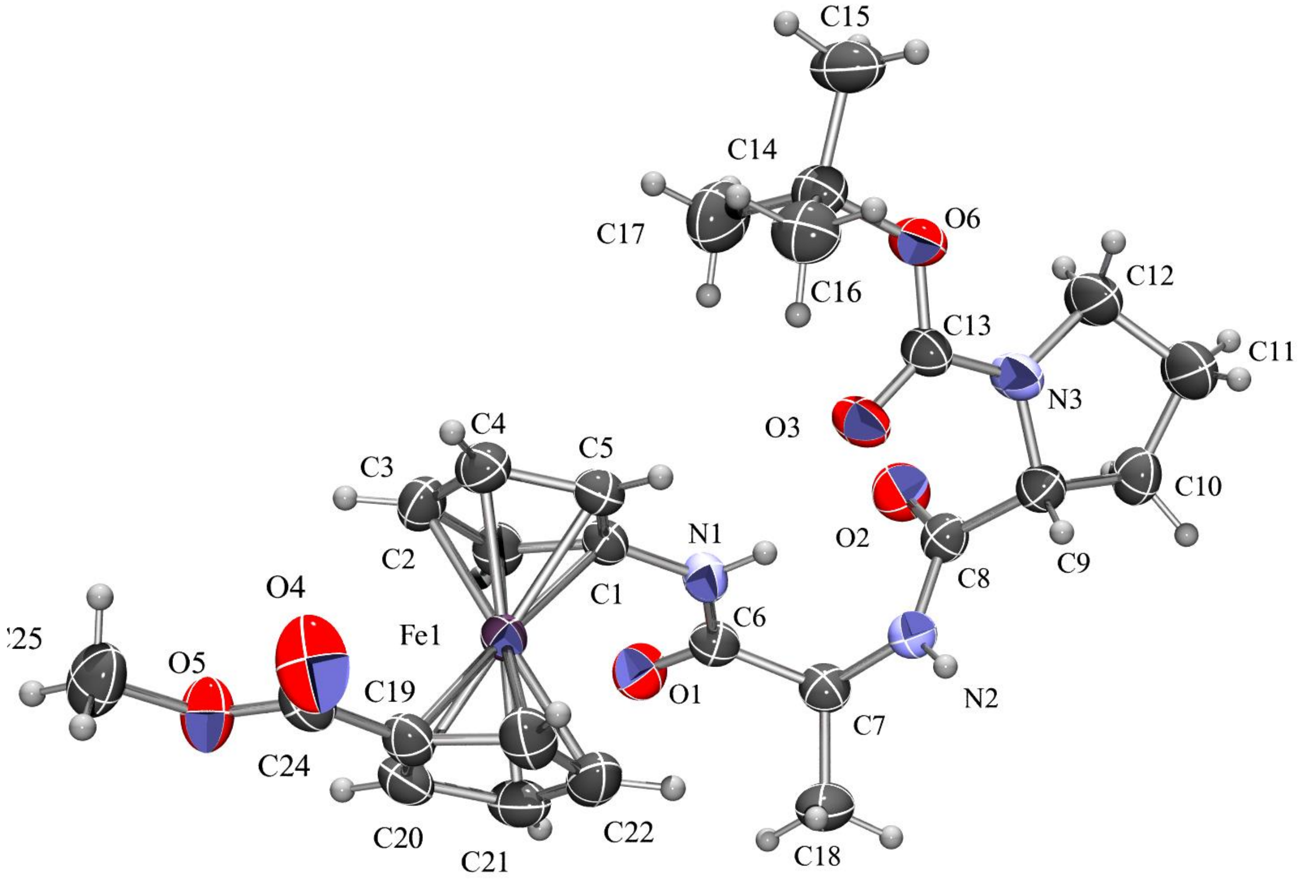

Figure S53. ORTEP-3 drawing of a molecule of **2**. Displacement ellipsoids are drawn for the probability of 50 % and hydrogen atoms are shown as spheres of arbitrary radii.

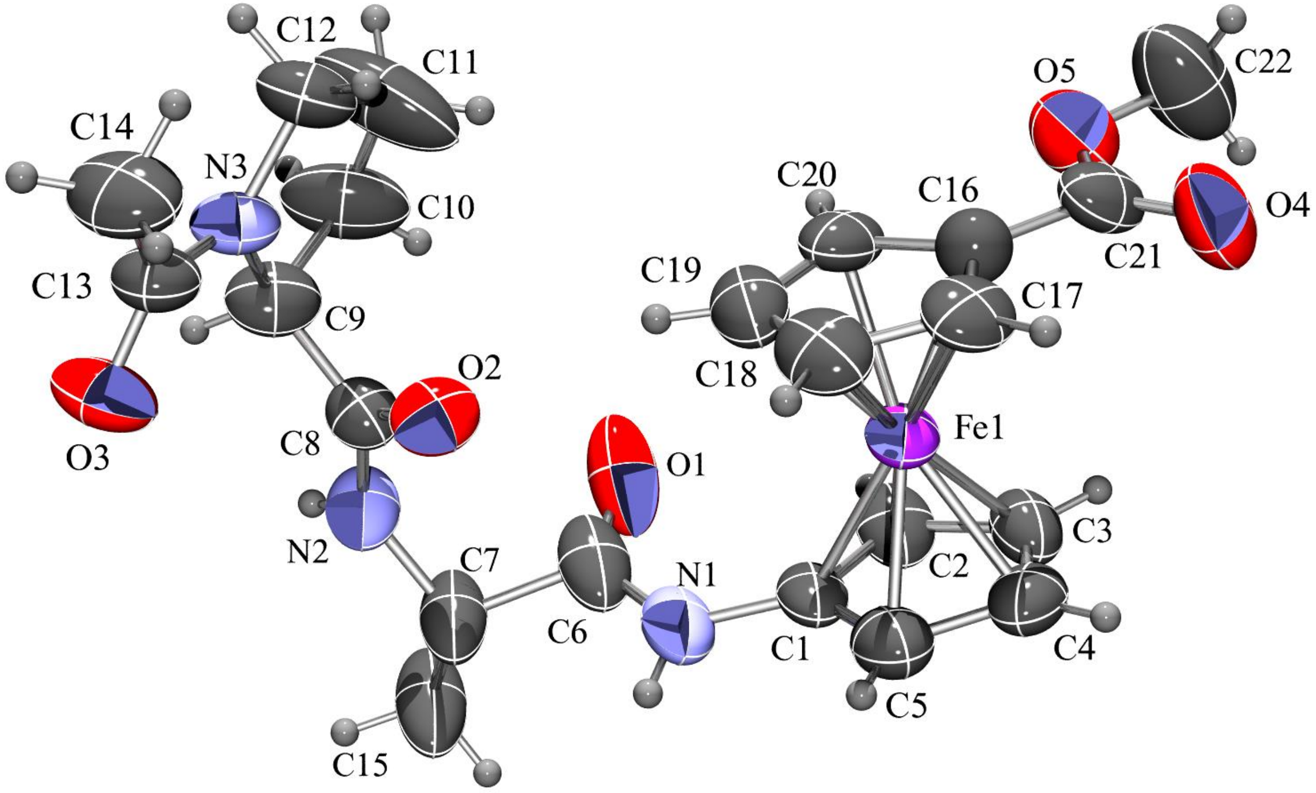

Figure S54. ORTEP-3 drawing of a molecule of **5**. Displacement ellipsoids are drawn for the probability of 50 % and hydrogen atoms are shown as spheres of arbitrary radii.

Table S55. Geometric parameters of hydrogen bonding (Å, °).

	D–H / Å	H···*A* / Å	*D*···*A* / Å	*D*–H···*A* / º	Symm. op. on *A*
			**2**		
N1–H1···O3	0.86	2.11	2.898(3)	151	*x*, *y*, *z*
N1–H1···N2	0.86	2.32	2.749(3)	111	*x*, *y*, *z*
N2–H2···O1	0.86	2.11	2.954(3)	168	–1+*x*, *y*, *z*
C2–H2A···O1	0.93	2.51	2.940(3)	108	*x*, *y*, *z*
C5–H5···O3	0.93	2.80	3.326(4)	117	*x*, *y*, *z*
C10–H10A···O2	0.97	2.55	3.226(3)	127	–1+*x*, *y*, *z*
C16–H16B···O3	0.96	2.34	2.961(3)	122	*x*, *y*, *z*
C17–H17C···O3	0.96	2.45	3.048(4)	120	*x*, *y*, *z*
C4–H4···O2	0.93	2.80	3.598(4)	144	*x*, –1/2+*y*, 3/2–*z*
C23–H23··O5	0.93	2.71	3.442(4)	136	–1+*x*, *y*, *z*
C15–H15C···O5	0.96	2.79	3.723(4)	165	1/2–*x*, –*y*, –1/2+*z*
			**5**		
N1–H1···O6	0.86	1.95	2.754(16)	156	*x*, *y*, *z*
N2–H2···O3	0.86	2.01	2.847(12)	163	1/2–*x*, –1/2+*y*, –*z*
C2–H2A···O1	0.93	2.59	2.980(11)	106	*x*, *y*, *z*
C10–H10B···O2	0.97	2.53	3.158(13)	122	*x*, –1+*y*, *z*
C15–H15C···O6	0.96	2.53	3.37(2)	145	*x*, –1+*y*, *z*
C19–H19···O2	0.93	2.42	3.325(10)	164	*x*, *y*, *z*
C12–H12A···O4	0.96	2.68	3.174(12)	112	1/2–*x*, –1/2+*y*, 1–*z*
C5–H5···O1	0.93	2.71	3.519(11)	147	*x*, 1+*y*, *z*

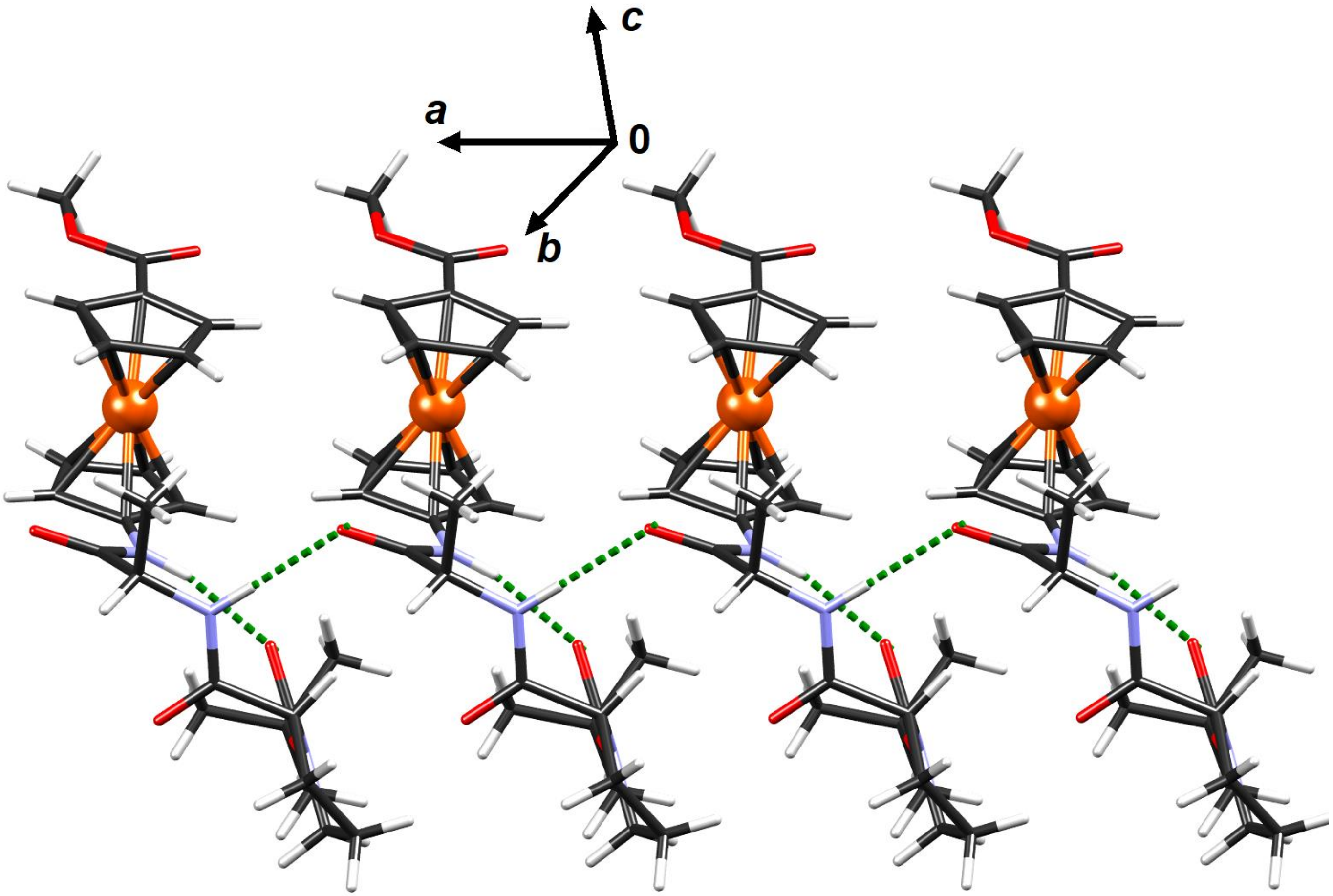

Figure S56. Hydrogen bonded chains in crystal packing of compound **2**. Hydrogen bonds are shown as dashed lines.

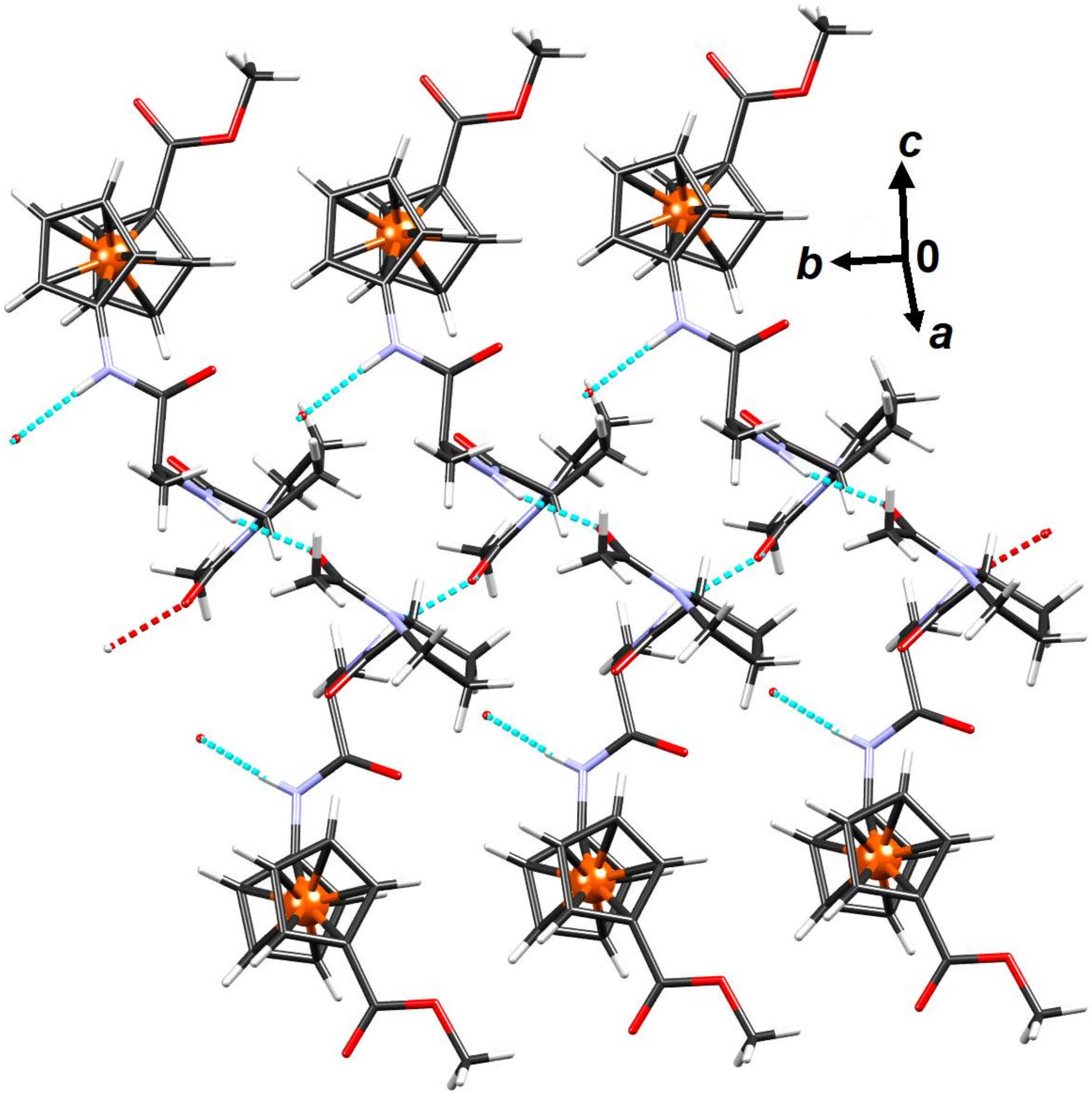

Figure S57. Hydrogen bonded chains in crystal packing of compound **5**. Hydrogen bonds are shown as dashed lines.

Biological evaluation

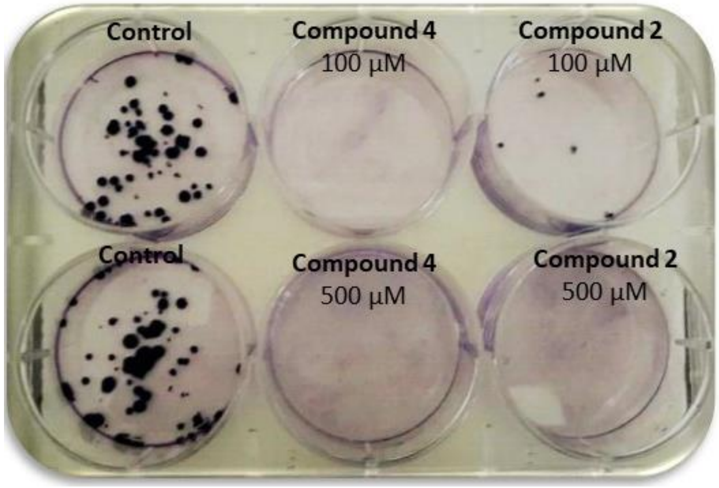

(**a**)

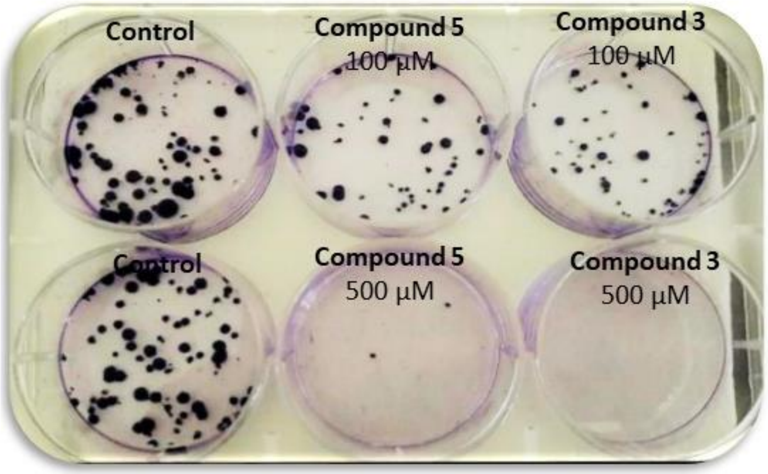

(**b**)

Figure S58. Results of clonogenic analysis after treatment with peptides **2-5** with two different concentrations [100 μM (**a**) and 500 μM (**b**)].
